# Catalyzing sustainable fisheries management through behavior change interventions

**DOI:** 10.1111/cobi.13475

**Published:** 2020-04-15

**Authors:** Gavin McDonald, Molly Wilson, Diogo Veríssimo, Rebecca Twohey, Michaela Clemence, Dean Apistar, Stephen Box, Paul Butler, Fel Cesar Cadiz, Stuart J. Campbell, Courtney Cox, Micah Effron, Steve Gaines, Raymond Jakub, Roquelito H. Mancao, Pablo T. Rojas, Rocky Sanchez Tirona, Gabriel Vianna

**Affiliations:** ^1^ Marine Science Institute, Santa Barbara – Marine Science Building University of California Santa Barbara CA 93106 U.S.A.; ^2^ Bren School of Environmental Science & Management University of California Santa Barbara – 2400 Bren Hall Santa Barbara CA 93106 U.S.A.; ^3^ Department of Zoology, Oxford Martin School University of Oxford 34 Broad St Oxford OX1 3BD U.K.; ^4^ Coral Reef Alliance 1330 Broadway #600 Oakland CA 94612 U.S.A.; ^5^ Rare Philippines 91–104 F. Ramos St Cebu City Cebu 6000 Philippines; ^6^ Rare – 1310 N Courthouse Rd Suite 110 Arlington VA 22201 U.S.A.; ^7^ Rare Indonesia – Jl. Gunung Gede I No.6 RT.3/RW.4, Bantarjati, Bogor Utara Kota Bogor Jawa Barat 16153 Indonesia; ^8^ Rua Visconde de Pirajá 177‐sala 801, Ipanema Rio de Janeiro RJ Brazil; ^9^ Current address: University of Western Australia 35 Stirling Hwy Crawley WA 6009 Australia

**Keywords:** behavior change campaigns, fisheries management, impact evaluation, monitoring and evaluation, perceptions data, small‐scale fisheries, social marketing, TURF reserve, campañas de cambio de comportamiento, datos de percepción, evaluación de impacto, manejo de pesquerías, mercadotecnia social, monitoreo y evaluación, pesquerías a pequeña escala, reserva TURF, 行为改变活动, 渔业管理, 影响评估, 监测和评估, 认知数据, 小型渔业, 社会营销, TURF 保护区

## Abstract

Small‐scale fisheries are an important livelihood and primary protein source for coastal communities in many of the poorest regions in the world, yet many are overfished and thus require effective and scalable management solutions. Positive ecological and socioeconomic responses to management typically lag behind immediate costs borne by fishers from fishing pressure reductions necessary for fisheries recovery. These short‐term costs challenge the long‐term success of these interventions. However, social marketing may increase perceptions of management benefits before ecological and socioeconomic benefits are fully realized, driving new social norms and ultimately long‐term sustainable behavior change. By conducting underwater visual surveys to quantify ecological conditions and by conducting household surveys with community members to quantify their perceptions of management support and socioeconomic conditions, we assessed the impact of a standardized small‐scale fisheries management intervention that was implemented across 41 sites in Brazil, Indonesia, and the Philippines. The intervention combines TURF reserves (community‐based territorial use rights for fishing coupled with no‐take marine reserves) with locally tailored social‐marketing behavior change campaigns. Leveraging data across 22 indicators and 4 survey types, along with data from 3 control sites, we found that ecological and socioeconomic impacts varied and that communities supported the intervention and were already changing their fishing practices. These results suggest that communities were developing new social norms and fishing more sustainably before long‐term ecological and socioeconomic benefits of fisheries management materialized.

## Abstract 2

*Article impact statement*: Moving from overfishing to sustainability, behavior change campaigns can be used to transition fisheries from near‐term losses to long‐term gains.

## Abstract (es)

Catalización del Manejo de Pesquerías Sustentables por medio de Intervenciones de Cambio de Comportamiento

**Resumen**

Las pesquerías a pequeña escala son un sustento importante y la principal fuente de proteína para las comunidades costeras en muchas de las regiones más pobres del mundo, sin embargo, muchas sufren de sobrepesca y por lo tanto requieren soluciones de manejo efectivas y escalables. Las respuestas ecológicas y socioeconómicas positivas al manejo generalmente se retrasan con respecto a los costos inmediatos asumidos por los pescadores a partir de las reducciones de la presión ocasionada por la pesca necesarias para la recuperación de la pesquería. Estos costos a corto plazo son un reto para el éxito a largo plazo de las intervenciones. Sin embargo, la mercadotecnia social puede incrementar las percepciones de los beneficios del manejo antes de que se conozcan por completo los beneficios ecológicos y socioeconómicos, lo que genera nuevas normas sociales y finalmente un cambio en el comportamiento a largo plazo. Realizamos censos submarinos visuales para cuantificar las condiciones ecológicas y censos domésticos a los miembros de la comunidad para cuantificar sus percepciones del apoyo al manejo y de las condiciones socioeconómicas. Con esto, evaluamos el impacto de la intervención de manejo para pesquerías estandarizadas a pequeña escala que fue implementada en 41 sitios de Brasil, Indonesia y las Filipinas. La intervención combina las reservas TURF (derechos de uso de pesca territorial basados en la comunidad acoplados con reservas marinas con restricción de pesca) con campañas de mercadotecnia social para el cambio de comportamiento ajustadas al contexto local. Con la movilización de datos a lo largo de 22 indicadores y cuatro tipos de censos, junto con los datos de tres sitios de control, encontramos que los impactos ecológicos y socioeconómicos variaron y que las comunidades apoyaban la intervención y ya se encontraban cambiando sus prácticas de pesca. Estos resultados sugieren que las comunidades ya estaban desarrollando nuevas normas sociales y pescando de manera más sustentable antes de que los beneficios ecológicos y socioeconómicos a largo plazo del manejo de las pesquerías se materializaran.

## 摘要

小型渔业是世界上许多最贫困地区沿海社区的重要生计和主要蛋白质来源, 但许多地区存在着过度捕捞的现象, 因此需要制定有效且灵活的管理方案以应对这一问题。管理措施产生的积极的生态及社会经济响应通常滞后于渔民为渔业恢复需要减少捕捞压力而承担的直接成本。这些短期成本压力给干预措施的长期成功带来挑战。然而, 在生态和社会经济效益完全实现之前, 社会营销或有助于增进对管理效益的认知, 从而推动建立新的社会规范, 并最终实现长期可持续的行为改变。我们通过水下观测量化了水体生态条件, 并通过社区成员家庭调查量化了他们对管理支持和社会经济条件的看法, 从而评估了在巴西、印度尼西亚和菲律宾的 41 个地点实施的标准化小型渔业管理措施的影响。这些管理措施结合了 TURF 保护区 (基于社区的渔业捕捞领土使用权与禁止捕捞的海洋保护区) 与适应当地情况的行为改变社会营销活动。我们利用 22 项指标和 4 种调查类型的数据, 加上来自 3 个对照位点的数据, 发现管理对生态和社会经济的影响各不相同, 此外, 社区成员支持干预措施并已经改变他们的捕捞方式。这些结果表明, 在渔业管理的长期生态及社会经济效益实现之前, 社区已开始发展新的社会规范, 并以更可持续性的方式开展渔业活动。**【翻译: 胡怡思; 审校: 聂永刚】**

## Introduction

Small‐scale fisheries provide livelihoods for more than 90% of the planet's fishers (Franz & Stamoulis 2015). However, overfishing has driven many fisheries to unsustainable conditions (Costello et al. 2012). Territorial use rights for fishing coupled with no‐take marine reserves (TURF reserves) have been touted as one solution for improving biological and socioeconomic conditions of small‐scale near‐shore fisheries (Afflerbach et al. 2014). There is theoretical support that TURF reserves can increase harvest and conservation when they are designed to encompass the species home range and when fishing pressure is otherwise poorly controlled (Lester et al. 2017). However, empirical evidence usually focuses separately on either TURFs (Quynh et al. 2017) or no‐take marine protected areas, also known as marine reserves or no‐take zones (NTZs) (Lester et al. 2009). Moreover, there have been no studies that empirically investigate the effectiveness of TURF reserves when coupled with social marketing. Social marketing aims to influence specific behavior change associated with sustainability and social good by using marketing techniques and is described as an important tool for successful adoption of conservation interventions (Salazar et al. 2018).

The aim of TURF reserves is to reduce fishing pressure by restricting access to the fishery and establishing management controls for those with fishing rights. Although theory predicts they will produce long‐term benefits when properly designed, ecological responses may be inherently slow due to life history traits of target species (Russ & Alcala 2004). Socioeconomic benefits may also be slow to materialize and fishers may even incur short‐term costs, especially when catches must be reduced to allow stocks to recover (Ovando et al. 2016). This temporary period presents a behavioral challenge because communities may slip back to overfishing when they fail to see dividends. Social norm‐based interventions could be uniquely situated to address this problem. Social sanctions for deviation that accompany social norms may create enabling conditions for compliance with that norm, even if that outcome is temporarily inferior to overfishing (Boyd & Richerson 1992). This stability could help a community comply until fish stocks are restored. Community support of the intervention is therefore critical for long‐term behavior change (Bennett 2016) and may build more quickly if the intervention includes a behavior change social‐marketing component (Veríssimo & McKinley 2016). A meta‐analysis of 84 conservation‐oriented social marketing campaigns showed that on average, all indicators of social‐norm and behavior change show positive changes following the intervention (Green et al. 2019). This suggests that behavior change campaigns may help address a ubiquitous small‐scale fisheries management challenge.

A recent initiative provided an opportunity for examining the efficacy of TURF reserves coupled with behavior change campaigns across a wide range of small‐scale fisheries contexts. Fish Forever is a global program aimed at improving the ecological, economic, and social conditions of small‐scale fishing communities. The program implemented local Pride campaigns, a unique style of social marketing pioneered by Rare (Jenks et al. 2010), and provided decision‐support tools to guide technical TURF‐reserve fisheries management. Implementation of the intervention was guided through a common theory of change (ToC) across all sites. Fundamentally, the intervention works through the creation of a new social norm of sustainable fishing, where fishers moderate their fishing because of the beliefs and behavior of those in their community (Bicchieri 2016).

We evaluated the effects of the program across 41 sites in Brazil, Indonesia, and the Philippines implemented from 2014 to 2017. First, we assessed biological and socioeconomic responses to the intervention across a diverse suite of indicators, including community support of the intervention, behavior change, and ecological and socioeconomic impact. Second, we supplemented a subset of these findings with 3 Philippines control sites to provide limited counterfactual evidence on what would have happened without the intervention at these 3 sites. Third, we used structural equation modeling to explore linkages between indicators across the ToC.

## Methods

### Study Areas

**Figure 1:**
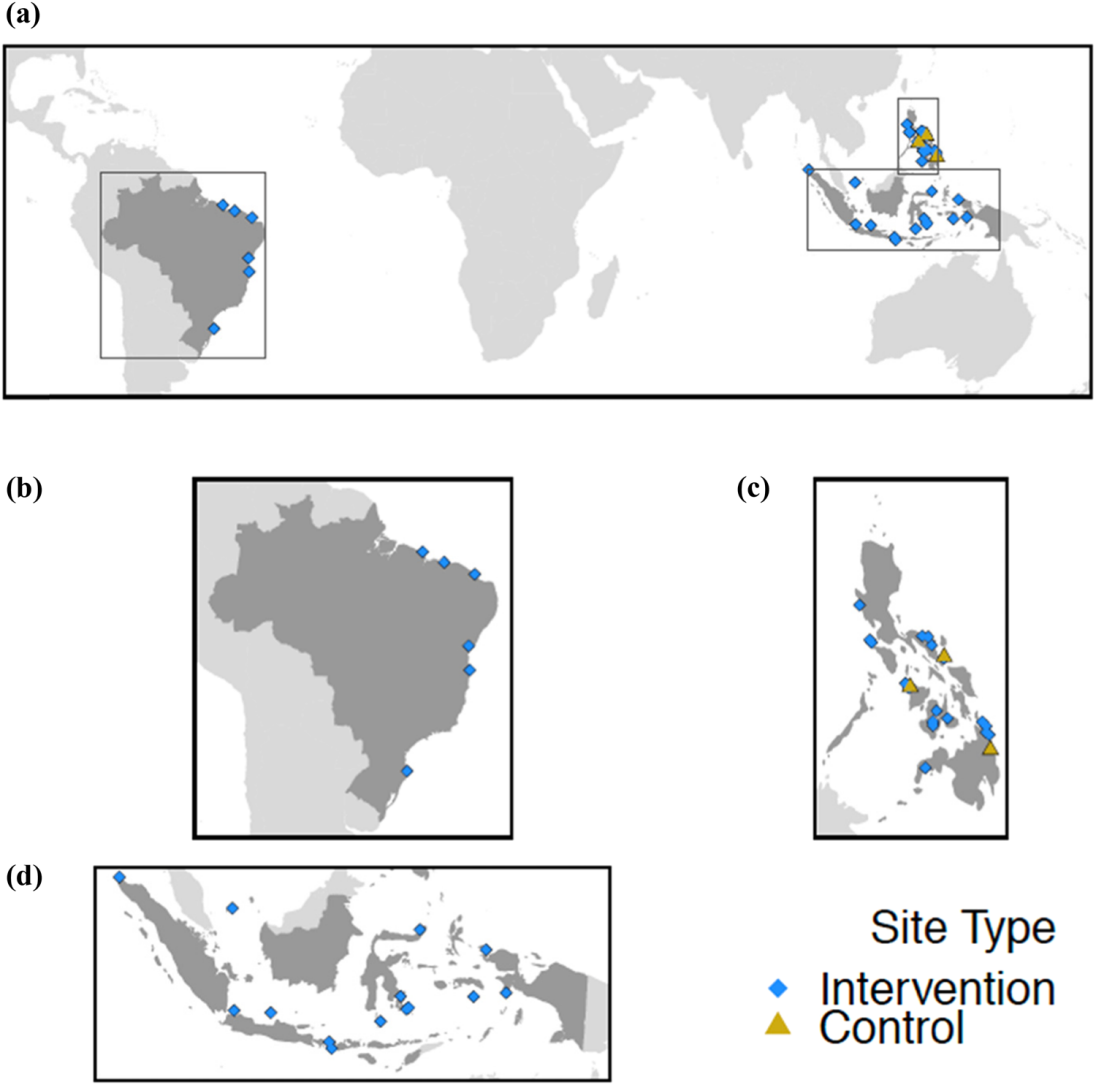
Locations of intervention sites where social marketing campaigns and TURF‐reserve implementation took place and control sites where no intervention took place for (a) all sites globally, (b) Brazil sites, (c) Philippines sites, and (d) Indonesia sites (TURF, territorial use rights for fishing).

The intervention and control sites were located across most of the major island groups in Indonesia and the Philippines and spread along the length of the Brazilian coastline (Fig. 1). Summary statistics of the intervention sites, including size of the TURF and NTZ, percentage of the TURF covered by NTZ, and number of affected fishers, are in the Supporting Information.

### Theory of Change

**Figure 2:**
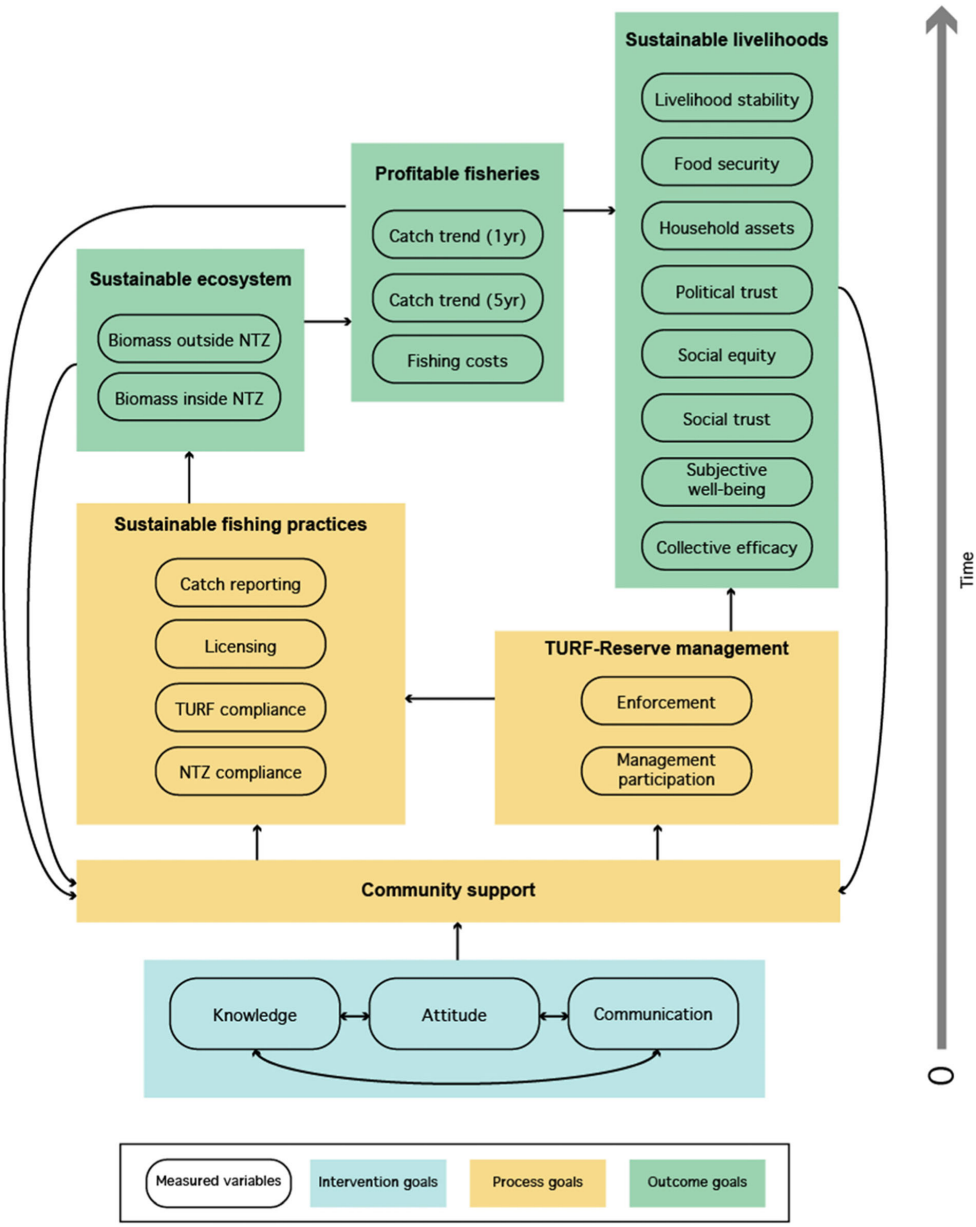
Simplified representation of Fish Forever's theory of change (ToC) with currently monitored indicators (rectangles, Fish Forever's overarching project goals; ovals, monitored indicators within each category; arrows, hypothesized relationships among larger project components; NTZ, no‐take marine reserve; TURF, territorial use rights for fishing).

Through a ToC, Fish Forever aimed to achieve a series of intervention and process goals (Fig. 2). Although more nuanced ToCs may be relevant at site‐ or country‐level scales, the following overarching Fish Forever ToC was kept as simple as possible to maintain relevance in different contexts while still reflecting key behavior change dynamics. The literature suggests that seeing a situation as competitive versus cooperative influences one's behavior (Engel & Rand 2014). Many fishers implicitly see fishing as a competitive interaction, where a fish taken by one person is a fish unavailable to everyone else. The intervention supplanted this belief with the knowledge that fisheries are in fact a cooperative dilemma: individuals have a selfish incentive to overfish, but everyone can do better by fishing sustainably. The intervention did so by using social marketing to promote knowledge, attitudes, and interpersonal communication related to sustainable fishing, which are keys to facilitating shifts in cooperation (Ostrom et al. 1992). This was done through the use of Pride campaigns, a type of social marketing led by a local campaign manager who is embedded in the community and a local implementing partner organization. Pride campaigns aim to drive behavior change around specific conservation actions and leverage audience research and segmented marketing through platforms including parades, banners, and radio (Jenks et al. 2010).

The intervention also provided essential technical knowledge needed to fish more sustainably through 2 decision‐support toolkits that helped communities delineate TURF‐reserve boundaries (Oyanedel et al. 2017) and establish new fishery management controls, such as gear restrictions or size limits, as part of an adaptive management framework (McDonald et al. 2018). These toolkits were provided to intervention sites but not control sites and were used by local campaign managers who facilitated participatory processes involving fishers, government, and other local stakeholders.

Once new regulations are established, the decision to comply is more complex than a simple monetary decision that weighs expected costs and benefits—it also involves nonmonetary factors including social influence and moral values (Hatcher et al. 2000). Social marketing influences pro‐environmental social norms, even during campaigns that last 3 years or less (Green et al. 2019). New norms can be established rapidly once a critical mass of individuals is reached (Centola et al. 2018). Further, along with the social norm that it is wrong for people to overfish comes a willingness to socially sanction those who do through self‐enforcement (Ovando et al. 2013).

By building shared community and government support and empowering fishers, we hypothesized that the intervention directly increases social and political trust, equity, collective efficacy, and subjective well‐being. As fish stocks increase through increased sustainable fishing practices, catches may also stabilize or increase. Ultimately, more stable catch levels may also have positive impacts on livelihood stability, food security, and household assets. However, theory predicts that benefits will not be realized if there is significant species movement outside the TURF reserve into heavily fished open‐access areas and that adding reserves to TURFs may have a neutral or even negative impact on harvest and income if the fishery is otherwise well managed (Lester et al. 2017). Social marketing alone cannot compensate for biological constraints or insufficient management design, which could eventually result in a loss of community support and ultimately intervention failure. Therefore, adaptive management and capacity building are important aspects of the program so that the community is equipped for long‐term sustainability (McDonald et al. 2018).

### Data Description and Survey Design

The ToC provided the framework for a global monitoring and evaluation plan to assess impact across a diversity of indicators (Table 1). Three socioeconomic household surveys assessing community support, sustainable fishing practices, and sustainable livelihoods were conducted across all sites in all countries and collected indicators via direct questioning to elicit self‐reported perceptions (Supporting Information). Meanwhile, a sustainable ecosystem survey was conducted at sites in Indonesia and the Philippines. Unlike the coral reefs at Indonesia and Philippines sites, Brazil sites feature mangrove and estuarine systems; thus, a comparable ecosystem survey could not be conducted in Brazil. All surveys at each site were conducted before and after the intervention. Additionally, sustainable ecosystem and sustainable livelihoods surveys were conducted in the Philippines at 3 matched control sites, both before and after the intervention period. In contrast to Philippines and Indonesia, access rights for Brazil TURFs were not finalized by 2017. Therefore, observed changes in Brazil would only be related to Pride campaigns and capacity building of existing fisheries management regimes (Supporting Information).

**Table 1:** Summary of characteristics of community support, sustainable fishing practices, sustainable ecosystems, and sustainable livelihoods surveys conducted in Brazil, Philippines, and Indonesia

Survey	Indicator	Type of capital^*^	Survey type	Administrator	Unit	Country (administration year)
Community Support (CS)	attitude; communication; knowledge relating to catch reporting; enforcement; NTZ (no take zone) compliance; TURF compliance; management participation	human	household	site campaign manager	0–100 (normalized using either binary responses or Likert‐type scale)	Brazil (2015 and 2017), Indonesia (2014 and 2017), and Philippines (2014 and 2017)
Sustainable fishing practices (SFP)	BC relating to catch reporting; enforcement; NTZ compliance; TURF compliance	human	household	site campaign manager	0–100 (normalized using either binary responses or Likert‐type scale)	Brazil (2015 and 2017), Indonesia (2014 and 2017), and Philippines (2014 and 2017)
	management participation	political				
Sustainable ecosystem (SE)	biomass inside NTZ; biomass outside NTZ	natural	underwater visual survey of target species	third‐party contractors	Kg/Ha	Indonesia (2014 and 2017) and Philippines (2014 and 2017)
Sustainable livelihoods (SL)	subjective well‐being	human	household	site campaign manager (Brazil); third‐party contractors (Indonesia and Philippines)	0–100 (normalized using either binary responses or Likert‐type scale)	Brazil (2015 and 2017), Indonesia (2014 and 2017), and Philippines (2014 and 2017)
	livelihood stability	human				
	food security	human				
	social trust	social				
	social equity	social				
	collective efficacy	social				
	household assets	financial				
	political trust	political				

* From Department for International Development.

To measure progress toward community support and the sustainable fishing practices community support is hypothesized to drive, we adapted the framework of Green et al. (2019), which integrates a transtheoretical behavior change model (Prochaska & Norcross 2018), a diffusion of innovation model (Rogers 2010), and a planned behavior model (Ajzen 1991). Respondents were asked about 6 behavior change components of the intervention and whether they knew what the behavior change was (knowledge), if they believed the behavior change would benefit themselves and the fishery (attitude), whether they had discussed the behavior change with other members of the community (communication), and whether they personally changed their behavior (BC) (Supporting Information).

To measure progress toward achieving sustainable livelihoods goals, we selected a suite of indicators by using the sustainable livelihoods framework (SLF) from the Department for International Development (DFID 1999). The SLF is based on the concept that people require certain capitals to achieve sustainable livelihoods: human, social, physical, financial, natural, and political. We therefore selected indicators from across these capitals that we hypothesized would be affected by the intervention according to our ToC (Table 1 and Supporting Information).

Household survey questions were kept as consistent as possible across sites and countries while still allowing for differences in local social context, language, and institutions. Question framing was highly related to the objectives and specific interventions implemented at each site and were defined in community‐based discussions during the pre‐implementation phase of each intervention. A global monitoring and evaluation team trained campaign managers in sampling protocols and worked with them to tailor survey questions to local contexts while maintaining global consistency. Households were selected randomly from households actively engaged in the intervention target fisheries and were geographically stratified across the community. Surveys were performed with heads of these households; the vast majority of these individuals were males. However, given the broader dependence of these communities on fisheries, socioeconomic changes perceived by these households were also likely noticed to some extent by other community members.

For household surveys, we calculated a normalized response score for each indicator scaled from 0 to 100 (0, worst possible; 100, best possible score). For binary questions, a positive response was a 100 and a negative response was a 0. For questions with multiple Likert‐type response options along a gradient (e.g., from strongly agree to strongly disagree), the most positive response was a 100, the most negative response was a 0, and responses in between were given a score scaled from 0 to 100 and proportional to the number of response options. At some sites, multiple questions relating to the same indicator were asked, in which case we averaged values across the normalized indicator scores (Supporting Information). Combining or comparing normalized scores from binary and Likert‐type questions may introduce bias because respondents to Likert‐type responses may chose extreme options less frequently than more central options (Guilford 1954). In future studies, we recommend asking all survey questions on the same scale.

Although direct questioning may result in biased responses, deliberate survey design and interview training can reduce some biases (Catania 1999), and direct questioning remains a predominant technique for eliciting community support and socioeconomic indicators where objective techniques are not possible (Steg & Vlek 2009). The on‐site campaign manager conducted most community support, sustainable fishing practices, and sustainable livelihoods surveys due to logistical and financial constraints, whereas third‐party contractors conducted the Philippines and Indonesia sustainable livelihood surveys. Use of impartial third‐party interviewers may reduce social desirability bias, which is a systematic self‐reporting error that may occur when survey respondents try to project a positive image of themselves (Krumpal 2013). However, we acknowledge this bias may still result in inflated estimates of positive change. Objective measures of socioeconomic indicators (e.g., from government economic data or enforcement reports) could not be obtained for this study, but these could triangulate self‐reported data in future impact evaluations. Additionally, although household surveys of individuals cannot directly measure changes in social norms that occur at the community level, they can measure individual behavior changes that require social buy‐in.

The sustainable ecosystem survey in Indonesia and the Philippines is a standard coral reef monitoring underwater visual survey that measures biomass of target fished families within the TURF, both inside and outside the NTZ (English et al. 1997). Monitored target families included snappers, groupers, and parrotfish (complete list in the Supporting Information).

The participation of a community in Fish Forever is a voluntary process that requires a democratic representative of a community to complete an application, followed by a selection process. This was the case for both intervention sites and control sites. Fish Forever is implemented at the local level by a representative of the local government or of a local NGO, and local institutions were active in the design and the implementation of the intervention design. Data collection for this study was conducted with permission of the national and local governments. Free, prior, and informed consent was obtained verbally from village leaders and participants before conducting household surveys. Participants were informed about the survey, its purpose, and how the data would be used prior to consent. Personal identifiers were anonymized. All data remained strictly confidential throughout the analysis process and reporting.

### Before–After Analyses

For each indicator and intervention site, we performed a separate linear regression to determine changes from before to after the intervention for that indicator and site. In each separate linear regression survey, respondents were the unit of observation for community support, sustainable fishing practices, and sustainable livelihoods surveys and underwater visual survey location was the unit of observation for sustainable ecosystem surveys. The before–after estimator $\beta_{1}$ is the standardized effect size. Standard errors are robust to heteroscedasticity.

(1) $$\mathrm{Outcome}{\;}_{i}=\beta_{0}\;+\beta_{1}\;\mathrm{Before}\;\mathrm{After}{}_{i}+u_{i},$$

where *u* is the error term and *i* is unit of observation. For each indicator and country, we also calculated the precision‐weighted average effect size across all sites. This uses the inverse variance weight of each individual model result to give higher weighting to site‐level models with lower standard errors (Hartung et al. 2008). Because the before–after analysis does not include a counterfactual, results from this approach cannot imply causality; rather, they simply establish whether changes occurred.

### Difference‐in‐Difference Impact Analysis

Impact evaluation aims to compare real‐world observations to an inferred counterfactual scenario, with the objective being to rule out alternative explanations for the observed outcomes (Ferraro 2009). Control sites should therefore be as similar as possible to the intervention sites regarding the characteristics that are expected to influence the intervention outcomes. For the Philippines sustainable ecosystems and sustainable livelihoods surveys, a formal process was used to select 3 matched control sites where no Fish Forever interventions were implemented. First, several communities self‐selected by expressing interest in becoming a Fish Forever site. For each community, 30 ecological, socioeconomic, and governance baseline attributes were scored on a scale of 1 to 5. These included species richness, extent of illegal fishing, and social capacity for conservation efforts (attributes in the Supporting Information). Next, an aggregate coarse matching score was calculated for each site by summing the individual attribute scores (Supporting Information). After intervention sites were selected following a multiround process that considered matching scores, strength of candidate campaign manager and implementing partner, and logistical constraints, control sites were selected from the pool of sites that made it to the final round of this process. Self‐selection biases were therefore avoided because all control and intervention sites had applied to participate in Fish Forever and had already made it through initial screenings. Control sites were selected that were within 10 points of the intervention site based on the aggregate matching score and within 3 points of the intervention site based on each individual baseline attribute score. To assess how well the control and intervention sites were matched, we calculated the standardized mean difference between the intervention site and control site aggregate matching scores; maximum cutoff was 0.25, which represents reasonably similar sites (Stuart et al. 2013). Intervention and control sites were well matched (standardized mean difference of 0.225).

We performed a difference‐in‐difference analysis with linear regression for the 3 intervention sites and matched control sites. For each indicator and intervention site, we performed a separate linear regression as follows, where MatchingScore is the coarse aggregate matching score and therefore addresses selection bias. The difference‐in‐difference estimator $\beta_{3}$ is the standardized effect size. Standard errors are robust to heteroscedasticity.

(2) $$\begin{array}{ccc} \mathrm{Outcom}\;e_{i} & = & \beta_{0}\;+\beta_{1}\mathrm{Before}\;{\mathrm{After}}_{i}+\beta_{2}{\mathrm{Control}\;\mathrm{Impact}}_{i}\\ & & +\;\beta_{3}\mathrm{Before}\;\mathrm{After}\times {\mathrm{Control}\;\mathrm{Impact}}_{i}\\ & & +\;\beta_{4}\mathrm{Matching}\;\mathrm{Score}+u_{i} \end{array}$$

For each indicator, we again present the precision‐weighted average effect size across all sites.

### Structural Equation Model

To examine relationships among our ToC indicators and to better understand the potential causal pathways between intervention and outcomes, we developed a structural equation model (SEM). Structural equation modeling integrates several multivariate analyses to test hypothesized relationships among both manifest indicators (i.e., directly observed) and latent indicators (i.e., hypothesized constructs to explain behavior) (Grace 2006). Because the interventions were 3 years or less in duration, we did not include hypothesized future sustainable livelihood benefits indicators in our main SEM. We instead limited our main SEM to relationships between community support and sustainable fishing practices and tested an additional iteration that included sustainable ecosystem benefits (Supporting Information). Because of its high degree of hypothesized links to sustainable fishing practices, we defined *community support* as a latent indicator predicted by the manifest indicators of knowledge, attitude, and communication.

We ran the model in the lavaan package in R (Rosseel 2012) with a pooled data set from intervention sites across all 3 countries and used before–after differences in site level means. Sample sizes for some indicators precluded country‐specific SEM analyses at this point, although each indicator is represented by comparable survey questions across countries. We evaluated performance of model iterations with comparative fit index (CFI) values. The CFI compares *χ* ^2^ values of the model with a null model in which all indicators are uncorrelated.

## Results

### Before–After Analysis

**Figure 3:**
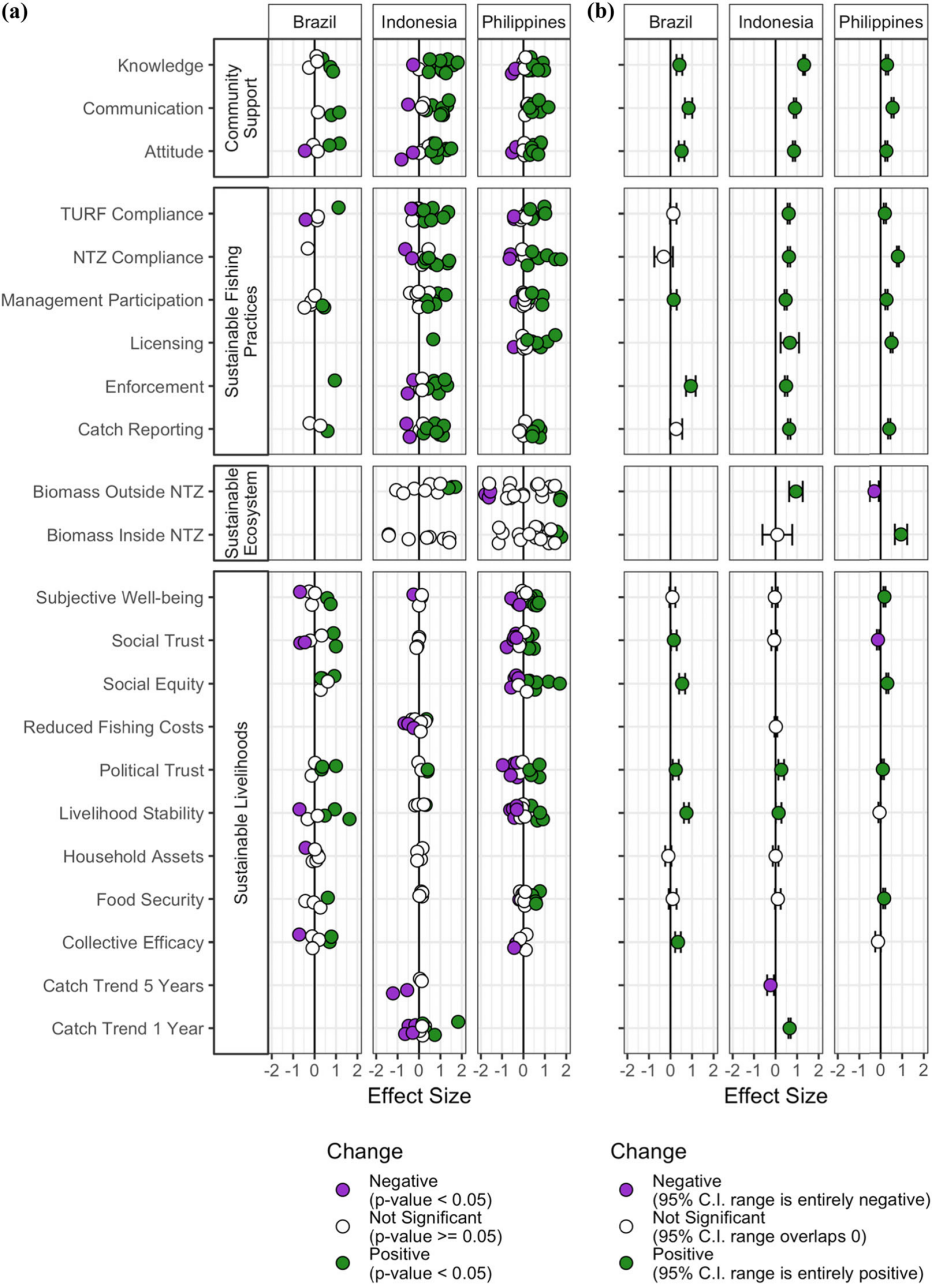
Before–after standardized effect sizes for all indicators from community support, sustainable fishing practices, sustainable ecosystems, and sustainable livelihoods surveys conducted in Brazil, Philippines, and Indonesia: (a) model results for each individual site‐indicator combination (points, regression result from individual site‐indicator combination), where linear regressions leverage survey respondents as the unit of observation for community support and sustainable fishing practices and sustainable livelihoods surveys and underwater visual survey locations are the units of observation for sustainable ecosystem surveys (vertical jitter is shown to allow visual differentiation between sites) and (b) precision‐weighted estimate across sites for each country‐indicator combination in (a) with 95% CIs (NTZ, no‐take zone marine reserve; TURF, territorial use rights for fishing).

Although there was heterogeneity across sites, some patterns emerged when looking at the precision‐weighted estimates at the country level (Fig. 3). All community‐support indicators describing knowledge, attitudes, and interpersonal communication related to the intervention showed statistically significant increases in all countries from before to after the intervention; precision‐weighted estimates ranged from 0.27 to 1.32. Most sustainable fishing‐practice indicators showed statistically significant positive changes, with the exception of 3 indicators in Brazil that showed no change (TURF compliance, NTZ compliance, and catch reporting). In Indonesia, targeted fish biomass increased outside the NTZ and did not change inside the NTZ, whereas Philippines biomass decreased outside the NTZ and increased inside the NTZ. Many sustainable‐livelihood indicators increased, although there was variability across countries regarding which indicators increased. Although livelihood stability increased across all 3 countries, social trust decreased in the Philippines and the perceived 5‐year catch trend decreased in Indonesia.

### Difference‐in‐Difference Impact Analysis

**Figure 4:**
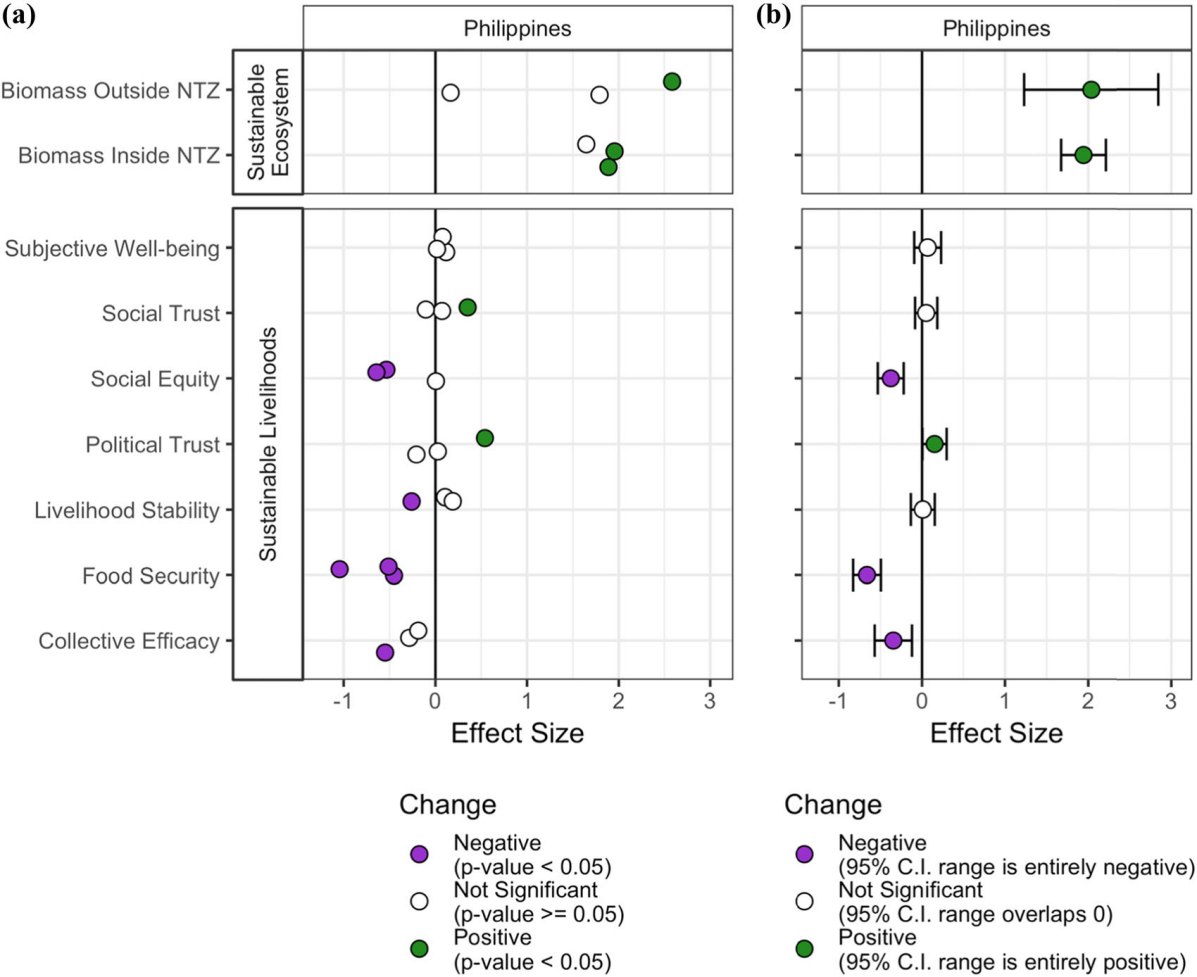
Difference‐in‐difference standardized effect sizes of indicators from the sustainable ecosystem and sustainable livelihoods surveys as they relate to observed changes at 3 Philippines intervention sites, where social marketing campaigns and TURF (territorial use rights for fishing)‐reserve implementation took place, relative to 3 matched Philippines control sites, where no intervention took place: (a) model results for each individual site‐indicator combination, where each point is the regression result from individual site‐indicator combination and linear regressions leverage survey respondents as the unit of observation for community support and sustainable fishing practices and sustainable livelihoods surveys and underwater visual survey locations are the units of observation for sustainable ecosystem surveys (vertical jitter is shown to allow visual differentiation between sites) and (b) precision‐weighted estimate across sites for each country‐indicator combination in (a) with 95% CIs.

There was again variability across individual sites, but certain patterns emerge when looking at country‐level precision‐weighted estimates (Fig. 4). Biomass increased inside and outside the NTZ relative to the control sites. Political trust also increased relative to the control sites, whereas social equity, food security, and collective efficacy decreased relative to control sites.

### Structural Equation Model

**Figure 5:**
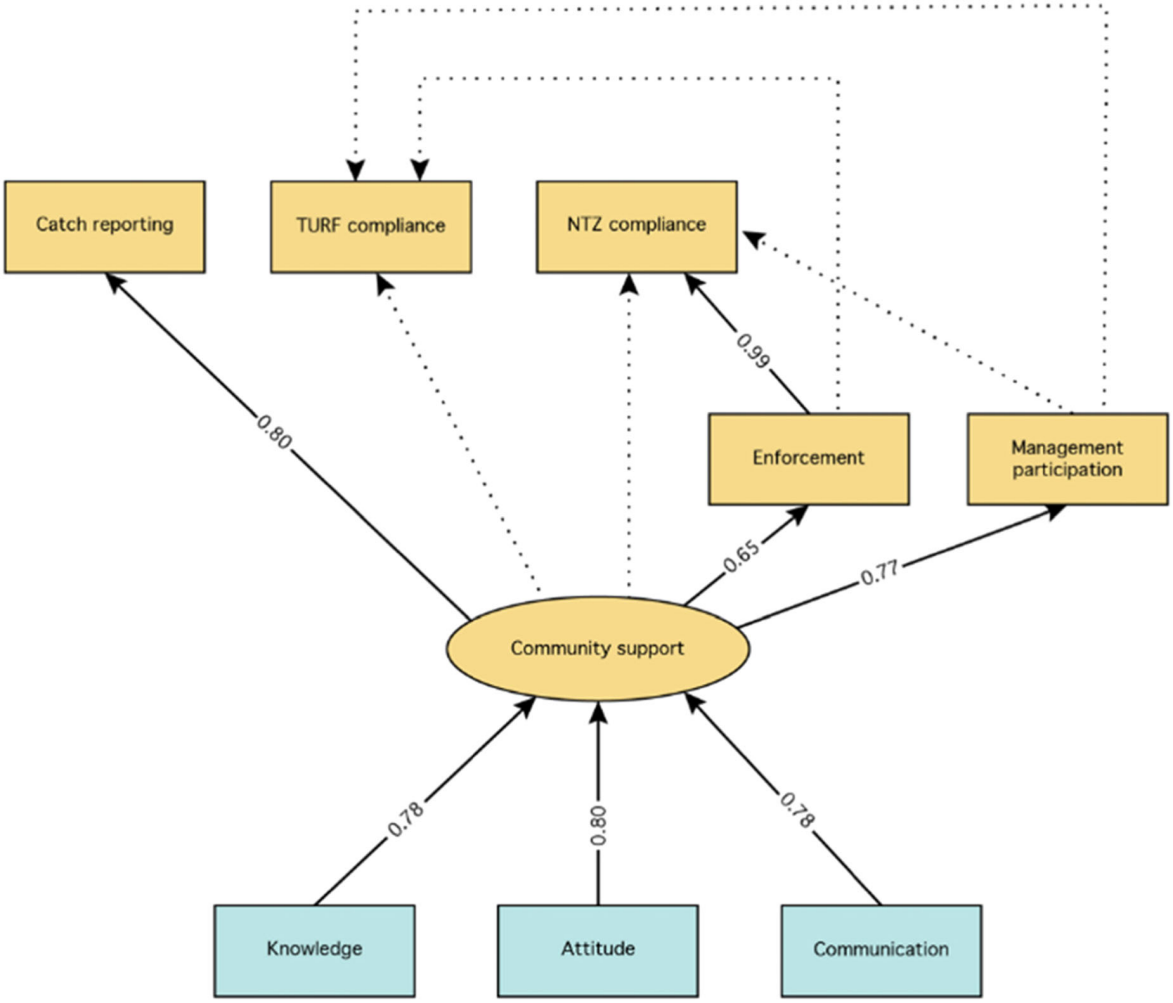
Structural equation model of relationships among monitored intervention (blue) and process (orange) indicators (boxes, manifest variables; ovals, our single latent variable; solid arrows, significant (*p* < 0.05) relationships [shown with factor loadings, β]; dotted arrows, tested but nonsignificant relationships). Comparative fit index is 0.908 (measure of model performance).

Overall the SEM performance (CFI = 0.908) met the desired threshold of 0.9. The model suggested that community support is a strong predictor of management participation, enforcement, and catch reporting, but not of NTZ compliance or TURF compliance (Fig. 5). Enforcement strongly predicted NTZ compliance but not TURF compliance. Management participation predicted neither NTZ nor TURF compliance. Measured ecological outcomes were not significantly predicted and their inclusion greatly reduced the performance of the model (CFI = 0.570) (Supporting Information).

## Discussion

For all 3 countries, positive impacts on all community‐support indicators suggested that the Fish Forever intervention created the social conditions needed for shifting behavior toward more sustainable fishing practices. We saw this result despite neutral or negative trends in several sustainable‐ecosystem and sustainable‐livelihood indicators, suggesting that behavior change interventions can build community support and social norms even while communities incur short‐term costs and before tangible results materialize. Before and after impact trends of the intervention were generally positive or neutral for ecosystem and sustainable‐livelihoods indicators, with the exception of social trust in the Philippines and perceived 5‐year catch trends in Indonesia, which both decreased. Limited counterfactual analyses in the Philippines showed that biomass improved inside and outside the NTZ relative to the control sites, whereas political trust was the only sustainable livelihoods indicator that improved relative to control sites. The intervention sites in the Philippines did not perform as well as the control sites in terms of food security, collective efficacy, and social equity. This provides complementary information to the results of the before–after analysis, which showed positive or neutral responses for these indicators. These negative results may be partially driven by near‐term socioeconomic costs and disruptions from marine reserves and more restrictive fisheries management at intervention sites (Christie 2004). Continued long‐term socioeconomic and ecological monitoring is needed to address temporal lags and to better understand the long‐term relationships between behavior changes, ecological responses, and livelihoods responses. Although our difference‐in‐difference conclusions are limited by the small number of control sites, these results provided a valuable complement to our before–after results.

### Community support

Positive changes were seen across knowledge, attitudes, and interpersonal communication indicators. Changes across this spectrum are illuminating and point toward changing social norms because knowledge alone is not enough to drive behavior change (Steg & Vlek 2009). There may also be heterogeneity in changes in attitudes based on resource dependency and livelihood diversification (Gelcich et al. 2005). Therefore, behavior change campaigns that aim to increase knowledge while also increasing interpersonal communication can positively influences attitudes, which in turn leads to positive behavior change (Green et al. 2019).

### Sustainable Fishing Practices

The structural equation model indicated the importance of community support in driving TURF‐reserve self‐enforcement, management participation, and catch reporting. We further found that self‐enforcement and reserve compliance were strongly associated. Although management participation increased across all countries and TURF and reserve compliance increased in Indonesia and Philippines, Brazil did not see increases in catch reporting or TURF or reserve compliance. These neutral results in Brazil could reflect the shorter intervention duration in Brazil, which was 2 years instead of 3, during which time TURF access rights had not yet been finalized. These results were reflected in the SEM, which showed that self‐enforcement did not significantly correlate with TURF compliance and that neither community support nor management participation significantly correlated with TURF compliance or reserve compliance. This contradicts previous findings that stakeholders who participate in the decision‐making process are more likely to comply and self‐enforce (Epstein 2017). Follow‐up monitoring should be conducted to determine whether sustainable fishing practices materialized and if they were maintained over time.

### Sustainable Ecosystems

Some positive before–after trends in target species biomass in Indonesia and Philippines suggest that compliance with reserves and adoption of new fisheries management controls may have led to biomass gains, as has been shown elsewhere (Campbell et al. 2018). Fish biomass in the Philippines increased relative to control sites inside and outside the NTZ. Increases in fish biomass within 3 years may be driven by species that are fast growing and spawn frequently, such as herbivorous fishes, including parrotfishes and rabbitfishes that are primary targets in many of the Indonesian and Philippines sites. It is unsurprising that in several sites no change in fish biomass was detected, most likely due to slower growing species and temporal lags that occur among behavior change, management adoption, and ecological response (Russ & Alcala 2004). The SEM results reflected these mixed ecological impacts because significant links from sustainable fishing practices to ecological indicators could not be established (Supporting Information).

### Sustainable Livelihoods

Across all 3 countries, the before–after improvement in most of the sustainable‐livelihoods indicators is consistent with findings that well‐being, social equity, and trust are indicative of successful co‐management approaches (Cinner et al. 2012). Political trust showed positive trends across all 3 countries, most likely because the strengthening of community institutions was well supported by fishing communities and not hindered by a lack of local government interest (Christie 2004). Political trust in the Philippines intervention sites also showed an increase relative to the control sites. However, we did observe several indicators with negative changes, perhaps indicative of the short‐term costs fishers may incur when moving to more restrictive sustainable management. Although data limitations prevented us from including livelihood impacts in our SEM, increased data availability across sites and time would allow us to more fully investigate predicted links within Fish Forever's ToC.

### Opportunities, Challenges, and Enabling Conditions

Effective impact evaluation is crucial to evidence‐based decision making and has been called for to improve conservation interventions and outcomes (Ferraro 2009). This is especially important when resources are limited, implementation costs are high, and intervention efficacy is uncertain (Pullin & Knight 2001). Fortunately, the field of monitoring and evaluation in marine conservation and small‐scale fisheries management is growing, and researchers are adopting innovative approaches to assess performance (Ahmadia et al. 2015).

From a practical perspective, impact evaluation has both challenges and opportunities. Data collection is expensive, and there may not be budget for collecting data at control sites or for continued long‐term monitoring. Whenever possible, objective measures of social change should be used to complement direct questioning methods to mitigate social desirability bias. When assessing social impact across a wide range of contexts, a global monitoring and evaluation team can work with local survey administrators across sites to ensure question intention is consistent while allowing for contextualized framing. Finally, certain data types are useful not only for impact evaluation, but also for adaptive fisheries management, a positive benefit exchange for stakeholders.

Behavior change campaigns can play a critical role in building and sustaining positive perceptions and behavior change in small‐scale fisheries management interventions. Although theory predicts that managed access interventions such as TURF reserves may eventually lead to long‐term benefits, they may also necessitate short‐term reductions in catch due to sustainable yet more restrictive management. Despite our results that intervention sites sometimes experienced short‐term neutral or negative changes across several sustainable‐livelihoods indicators, these same communities experienced increases in intervention support and shifts toward sustainable fishing behaviors. This highlights the role of social marketing behavior change campaigns as tools that can help managers overcome the short‐term challenges faced when implementing new management regimes.

## Data and Code Availability

All data and code necessary for reproducing this analysis can be found at: https://github.com/emlab-ucsb/fisheries-behavior-change. The DOI for this code and data repository is managed through Zenodo with DOI number https://doi.org/10.5281/zenodo.3635980.

## Supporting information

Supplementary figures and tables, including intervention‐site summary statistics and alternative results of the structural equation model (including ecological response indicators, control‐site aggregate coarse matching scores, representative socioeconomic survey questions, sample sizes for all indicators and sites, and target fished families in the Philippines and Indonesia underwater visual ecological surveys) (Appendix S1), a summary of all questions used across surveys (Appendix S2), a summary of which questions were asked multiple ways (Appendix S3), the actual survey instruments (Appendix S4‐S20), and a complete list of control‐site matching attributes and scores for each attribute at matched control and intervention sites (Appendix S21) are available online. The authors are solely responsible for the content and functionality of these materials. Queries (other than absence of the material) should be directed to the corresponding author. All data and the R code used in these analyses are available from https://github.com/emlab‐ucsb/fisheries‐behavior‐change.

Supplementary Material

Supplementary Material

Supplementary Material

Supplementary Material

Supplementary Material

Supplementary Material

Supplementary Material

Supplementary Material

Supplementary Material

Supplementary Material

Supplementary Material

Supplementary Material

Supplementary Material

Supplementary Material

Supplementary Material

Supplementary Material

Supplementary Material

Supplementary Material

Supplementary Material

Supplementary Material

## References

[cobi13475-bib-0001] Afflerbach JC , Lester SE , Dougherty DT , Poon SE . 2014 A global survey of “TURF‐reserves”, territorial use rights for fisheries coupled with marine reserves. Global Ecology and Conservation 2:97–106.

[cobi13475-bib-0002] Ahmadia GN , Glew L , Provost M , Gill D , Hidayat NI , Mangubhai S , Purwanto , Fox HE . 2015 Integrating impact evaluation in the design and implementation of monitoring marine protected areas. Philosophical Transactions of the Royal Society of London. Series B, Biological Sciences 370:20140275. 26460128 10.1098/rstb.2014.0275 PMC4614732

[cobi13475-bib-0003] Ajzen I . 1991 The theory of planned behavior. Organizational Behavior and Human Decision Processes 50:179–211.

[cobi13475-bib-0004] Bennett NJ . 2016 Using perceptions as evidence to improve conservation and environmental management. Conservation Biology 30:582–592. 26801337 10.1111/cobi.12681

[cobi13475-bib-0005] Bicchieri C . 2016 Norms in the wild: how to diagnose, measure, and change social norms. Oxford University Press, Oxford, United Kingdom.

[cobi13475-bib-0006] Boyd R , Richerson PJ . 1992 Punishment allows the evolution of cooperation (or anything else) in sizable groups. Ethology and Sociobiology 13:171–195.

[cobi13475-bib-0008] Campbell SJ , Edgar GJ , Stuart‐Smith RD , Soler G , Bates AE . 2018 Fishing‐gear restrictions and biomass gains for coral reef fishes in marine protected areas. Conservation Biology 32:401–410. 28776761 10.1111/cobi.12996

[cobi13475-bib-0009] Catania JA . 1999 A framework for conceptualizing reporting bias and its antecedents in interviews assessing human sexuality. Journal of Sex Research 36:25–38.

[cobi13475-bib-0010] Centola D , Becker J , Brackbill D , Baronchelli A . 2018 Experimental evidence for tipping points in social convention. Science 360:1116–1119. 29880688 10.1126/science.aas8827

[cobi13475-bib-0011] Christie P . 2004 Marine protected areas as biological successes and social failures in Southeast Asia. American Fisheries Society Symposium 42:155–164.

[cobi13475-bib-0012] Cinner JE , et al. 2012 Comanagement of coral reef social‐ecological systems. Proceedings of the National Academy of Sciences 109:5219–5222. 10.1073/pnas.1121215109 PMC3325732 22431631

[cobi13475-bib-0013] Costello C , Ovando D , Hilborn R , Gaines SD , Deschenes O , Lester SE . 2012 Status and solutions for the world's unassessed fisheries. Science 338:517–520. 23019613 10.1126/science.1223389

[cobi13475-bib-0014] Department for International Development (DFID) . 1999 Sustainable livelihoods guidance sheets. DFID, London Available from https://www.livelihoodscentre.org/documents/114097690/114438878/Sustainable+livelihoods+guidance+sheets.pdf/594e5ea6-99a9-2a4e-f288-cbb4ae4bea8b?t=1569512091877 (accessed on April 6, 2020).

[cobi13475-bib-0015] Engel C , Rand DG . 2014 What does “clean” really mean? The implicit framing of decontextualized experiments. Economics Letters 122:386–389.

[cobi13475-bib-0016] English SA , Susan A , Baker VJ , Wilkinson CR . 1997 Survey manual for tropical marine resources. Australian Institute of Marine Science, Townsville, Australia Available from http://epubs.aims.gov.au/handle/11068/13062 (accessed on April 6, 2020).

[cobi13475-bib-0017] Epstein G . 2017 Local rulemaking, enforcement and compliance in state‐owned forest commons. Ecological Economics 131:312–321.

[cobi13475-bib-0018] Ferraro PJ . 2009 Counterfactual thinking and impact evaluation in environmental policy. New Directions for Evaluation 2009:75–84.

[cobi13475-bib-0019] Franz N , Stamoulis K . 2015 Small‐scale fisheries. Food and Agriculture Organisation, Rome, Italy Available from http://www.fao.org/3/a-au832e.pdf (accessed on April 6, 2020).

[cobi13475-bib-0020] Gelcich S , Edwards‐Jones G , Kaiser MJ . 2005 Importance of attitudinal differences among artisanal fishers toward co‐management and conservation of marine resources. Conservation Biology 19:865–875.

[cobi13475-bib-0021] Grace JB . 2006 Structural equation modeling and natural systems. Cambridge University Press, Cambridge, United Kingdom.

[cobi13475-bib-0022] Green KM , Crawford BA , Williamson KA , DeWan AA . 2019 A meta‐analysis of social marketing campaigns to improve global conservation outcomes. Social Marketing Quarterly 25:69–87.

[cobi13475-bib-0023] Guilford JP . 1954 Psychometric methods. 2nd ed. McGraw‐Hill, New York.

[cobi13475-bib-0024] Hartung J , Knapp G , Sinha BK . 2008 Statistical meta‐analysis with applications. Wiley, Hoboken, New Jersey.

[cobi13475-bib-0025] Hatcher A , Jaffry S , Thébaud O , Bennett E . 2000 Normative and social influences affecting compliance with fishery regulations. Land Economics 76:448–461.

[cobi13475-bib-0026] Jenks B , Vaughan PW , Butler PJ . 2010 The evolution of Rare Pride: using evaluation to drive adaptive management in a biodiversity conservation organization. Evaluation and Program Planning 33:186–190. 19733908 10.1016/j.evalprogplan.2009.07.010

[cobi13475-bib-0027] Krumpal I . 2013 Determinants of social desirability bias in sensitive surveys: a literature review. Quality & Quantity 47:2025–2047.

[cobi13475-bib-0028] Lester SE , McDonald G , Clemence M , Dougherty DT , Szuwalski CS . 2017 Impacts of TURFs and marine reserves on fisheries and conservation goals: theory, empirical evidence, and modeling. Bulletin of Marine Science 93:173–198.

[cobi13475-bib-0029] Lester S , Halpern B , Grorud‐Colvert K , Lubchenco J , Ruttenberg B , Gaines S , Airamé S , Warner R . 2009 Biological effects within no‐take marine reserves: a global synthesis. Marine Ecology Progress Series 384:33–46.

[cobi13475-bib-0030] McDonald G , et al. 2018 An adaptive assessment and management toolkit for data‐limited fisheries. Ocean & Coastal Management 152:100–119.

[cobi13475-bib-0031] Nguyen Thi Quynh C , Schilizzi S , Hailu A , Iftekhar S . 2017 Territorial use rights for fisheries (TURFs): state of the art and the road ahead. Marine Policy 75:41–52.

[cobi13475-bib-0032] Ostrom E , Walker J , Gardner R . 1992 Covenants with and without a sword: self‐governance is possible. American Political Science Review 86:404–417.

[cobi13475-bib-0033] Ovando DA , et al. 2013 Conservation incentives and collective choices in cooperative fisheries. Marine Policy 37:132–140.

[cobi13475-bib-0034] Ovando D , Dougherty D , Wilson JR . 2016 Market and design solutions to the short‐term economic impacts of marine reserves. Fish and Fisheries 17:939–954.

[cobi13475-bib-0035] Oyanedel R , et al. 2017 A decision support tool for designing TURF‐reserves. Bulletin of Marine Science 93:155–172.

[cobi13475-bib-0036] Prochaska JO , Norcross JC . 2018 Systems of psychotherapy: a transtheoretical analysis. Oxford University Press Oxford, United Kingdom.

[cobi13475-bib-0037] Pullin AS , Knight TM . 2001 Effectiveness in conservation practice: pointers from medicine and public health. Conservation Biology 15:50–54.

[cobi13475-bib-0038] Rogers EM . 2010 Diffusion of innovations. 4th ed Simon and Schuster, New York.

[cobi13475-bib-0039] Rosseel Y . 2012 lavaan: an R package for structural equation modeling. Journal of Statistical Software 48:1–36.

[cobi13475-bib-0040] Russ GR , Alcala AC . 2004 Marine reserves: long‐term protection is required for full recovery of predatory fish populations. Oecologia 138:622–627. 14716555 10.1007/s00442-003-1456-4

[cobi13475-bib-0041] Salazar G , Mills M , Veríssimo D . 2018 Qualitative impact evaluation of a social marketing campaign for conservation. Conservation Biology 33:634–644. 30178894 10.1111/cobi.13218

[cobi13475-bib-0042] Steg L , Vlek C . 2009 Encouraging pro‐environmental behaviour: an integrative review and research agenda. Journal of Environmental Psychology 29:309–317.

[cobi13475-bib-0043] Stuart EA , Lee BK , Leacy FP . 2013 Prognostic score‐based balance measures can be a useful diagnostic for propensity score methods in comparative effectiveness research. Journal of Clinical Epidemiology 66:S84–S90e1. 23849158 10.1016/j.jclinepi.2013.01.013 PMC3713509

[cobi13475-bib-0044] Veríssimo D , McKinley E . 2016 Introducing conservation marketing: Why should the devil have all the best tunes? Oryx 50:13–17.

