# Supplementary material for: Catalyzing sustainable fisheries management through behavior change interventions
Source: Conserv Biol. 2020 Apr 15;34(5):1176–89. doi: 10.1111/cobi.13475 (PMC7540413; doi:10.1111/cobi.13475)
Supplement: Supplementary file 1 — Supplementary figures and tables, including intervention‐site summary statistics and alternative results of the structural equation model (including ecological response indicators, control‐site aggregate coarse matching scores, representative socioeconomic survey questions, sample sizes for all indicators and sites, and target fished families in the Philippines and Indonesia underwater visual ecological surveys) (Appendix S1), a summary of all questions used across surveys (Appendix S2), a summary of which questions were asked multiple ways (Appendix S3), the actual survey instruments (Appendix S4‐S20), and a complete list of control‐site matching attributes and scores for each attribute at matched control and intervention sites (Appendix S21) are available online. The authors are solely responsible for the content and functionality of these materials. Queries (other than absence of the material) should be directed to the corresponding author. All data and the R code used in these analyses are available from https://github.com/emlab‐ucsb/fisheries‐behavior‐change. [file COBI-34-1176-s001.docx]

**Supplementary Information**


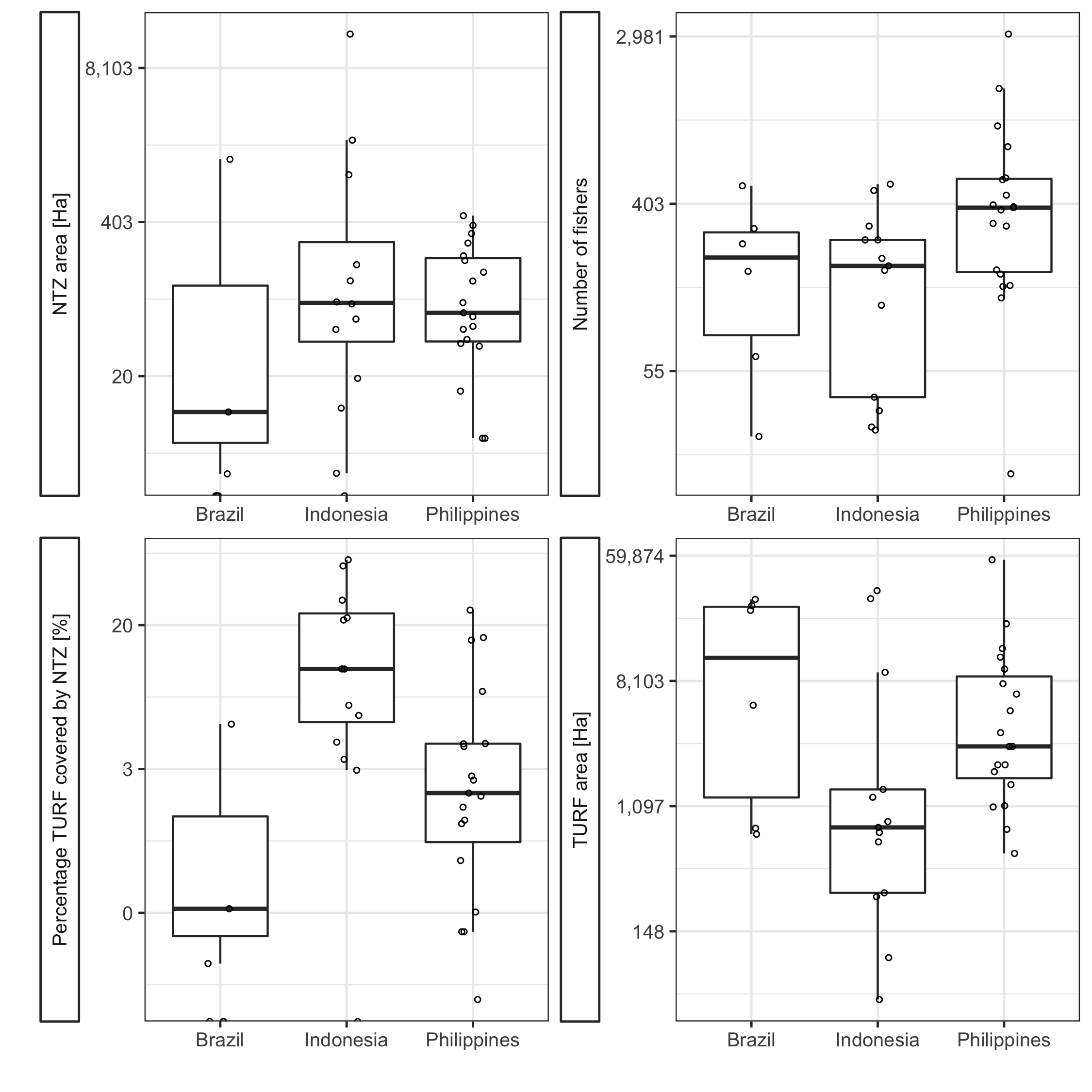


Figure S1: NTZ area [Ha], number of fishers, percentage TURF covered by NTZ [%], and TURF area [Ha] for all intervention sites across Brazil, Indonesia, and Philippines. Each point represents a single site; the middle line of the boxplot represents the median value, the bottom and top of the boxes are the 25^th^ and 75^th^ percentiles values respectively, the bottom whiskers are the 25^th^ percentile minus 1.5 times the interquartile range, and the top whiskers are the 75^th^ percentile plus 1.5 times the interquartile range. The y-axis is on a natural logarithmic scale.


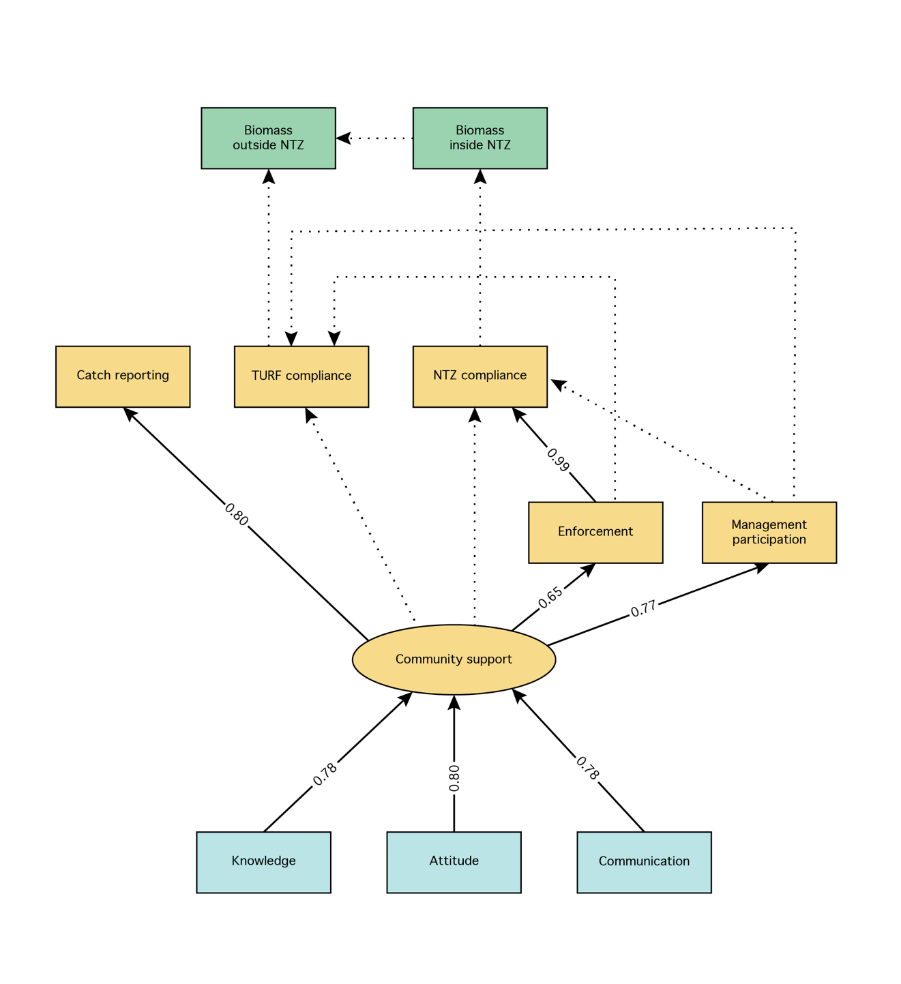


**Figure S2: Alternative structural equation model including ecological response indicators. Tested links from management and fishing practice indicators to ecological response indicators were not significant, and the inclusion of ecological indicators greatly reduced the performance of this model (CFI= 0.571).**

**Table S1:**  **Coarse matching scores for Philippines control and intervention sites for the sustainable livelihoods and sustainable ecosystem surveys**

| **Site** | **Intervention Site Score** | **Control Site Score** |
| --- | --- | --- |
| Culasi | 106 | 97.5 |
| Dapa | 96 | 102 |
| Gubat | 97.5 | 96.5 |

**Sample size Summary**

Table 2: Number of Fish Forever (FF) intervention sites and control sites that collected data by survey, indicator, and country. Survey acronyms are CS (Community Support), SFP (Sustainable Fishing Practices), SE (Sustainable Ecosystems), and SL (Sustainable Livelihoods).

| **Survey** | **Indicator** | **Brazil n FF** | **Indonesia n FF** | **Philippines n Control** | **Philippines n FF** |
| --- | --- | --- | --- | --- | --- |
| CS | Attitude | 6 | 13 | 0 | 13 |
| CS | Communication | 3 | 13 | 0 | 12 |
| CS | Knowledge | 6 | 13 | 0 | 13 |
| SFP | Catch Reporting | 3 | 12 | 0 | 13 |
| SFP | Enforcement | 1 | 12 | 0 | 0 |
| SFP | Licensing | 0 | 1 | 0 | 13 |
| SFP | Management Participation | 6 | 13 | 0 | 13 |
| SFP | NTZ Compliance | 1 | 12 | 0 | 12 |
| SFP | TURF Compliance | 4 | 13 | 0 | 12 |
| SE | Biomass Inside NTZ | 0 | 8 | 3 | 19 |
| SE | Biomass Outside NTZ | 0 | 10 | 3 | 19 |
| SL | Catch Trend 1 Year | 0 | 4 | 0 | 0 |
| SL | Catch Trend 5 Years | 0 | 4 | 0 | 0 |
| SL | Collective Efficacy | 6 | 0 | 3 | 5 |
| SL | Food Security | 4 | 4 | 3 | 6 |
| SL | Household Assets | 6 | 4 | 0 | 0 |
| SL | Livelihood Stability | 6 | 4 | 3 | 6 |
| SL | Political Trust | 6 | 4 | 3 | 6 |
| SL | Social Equity | 5 | 0 | 3 | 6 |
| SL | Social Trust | 6 | 4 | 3 | 6 |
| SL | Subjective Well-being | 6 | 4 | 3 | 6 |

Table S3: Sample size for each indicator collected at Brazil sites. Sample size is the number of individual survey respondents, with text denoted as “Sample size before intervention, Sample size after intervention.” Survey acronyms are CS (Community Support), SFP (Sustainable Fishing Practices), SE (Sustainable Ecosystems), and SL (Sustainable Livelihoods).

| **Survey** | **Indicator** | **BIGUAPE** | **CANAVIE** | **CURURUP** | **PARNAIB** | **PCVERDE** | **PIRAJUB** |
| --- | --- | --- | --- | --- | --- | --- | --- |
| CS | Attitude | 52, 42 | 98, 48 | 30, 49 | 129, 110 | 110, 67 | 17, 6 |
| CS | Communication | NA | 98, 48 | 27, 46 | NA | 110, 67 | NA |
| CS | Knowledge | 52, 42 | 98, 48 | 30, 49 | 129, 110 | 110, 67 | 17, 6 |
| SFP | Catch Reporting | NA | 98, 48 | 29, 49 | NA | NA | 17, 6 |
| SFP | Enforcement | NA | NA | NA | NA | 110, 67 | NA |
| SFP | Management Participation | 59, 49 | 98, 48 | 29, 49 | 117, 108 | 110, 67 | 17, 6 |
| SFP | NTZ Compliance | NA | NA | 30, 49 | NA | NA | NA |
| SFP | TURF Compliance | NA | 98, 48 | 29, 49 | 128, 110 | 110, 67 | NA |
| SL | Collective Efficacy | 59, 49 | 98, 48 | 30, 49 | 129, 110 | 110, 67 | 17, 6 |
| SL | Food Security | 58, 37 | 96, 48 | 28, 49 | NA | 107, 65 | 17, 3 |
| SL | Household Assets | 59, 49 | 98, 48 | 30, 49 | 129, 110 | 110, 67 | 17, 6 |
| SL | Livelihood Stability | 59, 49 | 98, 48 | 30, 49 | 129, 110 | 110, 67 | 17, 6 |
| SL | Political Trust | 59, 49 | 98, 48 | 30, 49 | 129, 110 | 110, 67 | 17, 6 |
| SL | Social Equity | NA | 98, 48 | 30, 49 | 129, 110 | 110, 67 | 17, 6 |
| SL | Social Trust | 59, 49 | 98, 48 | 30, 49 | 129, 110 | 110, 67 | 17, 6 |
| SL | Subjective Well-being | 59, 49 | 98, 48 | 30, 49 | 129, 110 | 110, 67 | 17, 6 |

Table S4: Sample size for each indicator collected at the first seven Indonesia sites. Sample size is the number of individual survey respondents or the number of underwater visual survey sampling locations, with text denoted as “Sample size before intervention at intervention site, Sample size after intervention at intervention site; Sample size before intervention at control site, Sample size after intervention at control site.” Survey acronyms are CS (Community Support), SFP (Sustainable Fishing Practices), SE (Sustainable Ecosystems), and SL (Sustainable Livelihoods).

| **Survey** | **Indicator** | **ANAMBAS** | **BANDAMP** | **BUMBANG** | **BUNAKEN** | **GILIMAT** | **KAIMANA** | **KARIMUN** |
| --- | --- | --- | --- | --- | --- | --- | --- | --- |
| CS | Attitude | 50, 49 | 193, 184 | 366, 372 | 38, 38 | 29, 25 | 27, 34 | 20, 54 |
| CS | Communication | 50, 47 | 193, 184 | 366, 372 | 38, 38 | 29, 25 | 27, 34 | 20, 54 |
| CS | Knowledge | 50, 44 | 193, 184 | 365, 372 | 38, 38 | 29, 25 | 27, 34 | 20, 54 |
| SFP | Catch Reporting | 50, 47 | 193, 184 | 366, 371 | 38, 38 | 29, 25 | 27, 34 | NA |
| SFP | Enforcement | 50, 47 | 193, 184 | 366, 371 | 38, 38 | 29, 25 | 27, 34 | NA |
| SFP | Licensing | NA | NA | NA | 38, 38 | NA | NA | NA |
| SFP | Management Participation | 50, 47 | 193, 184 | 366, 372 | 38, 38 | 29, 25 | 27, 33 | 20, 53 |
| SFP | NTZ Compliance | 50, 47 | 193, 184 | 366, 371 | 38, 38 | 29, 25 | 27, 34 | NA |
| SFP | TURF Compliance | 50, 47 | 193, 184 | 366, 371 | 38, 38 | 29, 25 | 27, 34 | 20, 54 |
| SE | Biomass Inside NTZ | 3, 3 | NA | NA | NA | 1, 1 | 3, 1 | 4, 4 |
| SE | Biomass Outside NTZ | 5, 4 | 3, 3 | NA | 2, 2 | 2, 2 | 7, 4 | 1, 1 |
| SL | Catch Trend 1 Year | NA | NA | NA | 38, 39 | NA | NA | NA |
| SL | Catch Trend 5 Years | NA | NA | NA | 20, 22 | NA | NA | NA |
| SL | Food Security | NA | NA | NA | 38, 39 | NA | NA | NA |
| SL | Household Assets | NA | NA | NA | 38, 39 | NA | NA | NA |
| SL | Livelihood Stability | NA | NA | NA | 38, 39 | NA | NA | NA |
| SL | Political Trust | NA | NA | NA | 38, 39 | NA | NA | NA |
| SL | Social Trust | NA | NA | NA | 38, 39 | NA | NA | NA |
| SL | Subjective Well-being | NA | NA | NA | 38, 39 | NA | NA | NA |

Table S5: Sample size for each indicator collected at the next seven Indonesia sites. Sample size is the number of individual survey respondents or the number of underwater visual survey sampling locations, with text denoted as “Sample size before intervention at intervention site, Sample size after intervention at intervention site; Sample size before intervention at control site, Sample size after intervention at control site.” Survey acronyms are CS (Community Support), SFP (Sustainable Fishing Practices), SE (Sustainable Ecosystems), and SL (Sustainable Livelihoods).

| **Survey** | **Indicator** | **KOLONOB** | **MAYALIB** | **SABANG_** | **SERIBU_** | **TAKABON** | **WAKA_DT** | **WAKA_NP** |
| --- | --- | --- | --- | --- | --- | --- | --- | --- |
| CS | Attitude | 309, 299 | NA | 67, 66 | 210, 210 | 185, 181 | 150, 150 | 262, 262 |
| CS | Communication | 309, 299 | NA | 67, 66 | 210, 210 | 185, 181 | 150, 150 | 262, 262 |
| CS | Knowledge | 309, 299 | NA | 67, 66 | 210, 210 | 185, 181 | 150, 150 | 262, 262 |
| SFP | Catch Reporting | 309, 299 | NA | 67, 66 | 210, 210 | 185, 181 | 150, 150 | 262, 262 |
| SFP | Enforcement | 309, 299 | NA | 67, 66 | 210, 210 | 185, 181 | 150, 150 | 262, 262 |
| SFP | Licensing | NA | NA | NA | NA | NA | NA | NA |
| SFP | Management Participation | 309, 299 | NA | 67, 66 | 210, 210 | 185, 181 | 150, 150 | 262, 262 |
| SFP | NTZ Compliance | 309, 299 | NA | 67, 66 | 210, 210 | 185, 181 | 150, 150 | 262, 262 |
| SFP | TURF Compliance | 309, 299 | NA | 67, 66 | 210, 210 | 185, 181 | 150, 150 | 262, 262 |
| SE | Biomass Inside NTZ | 2, 2 | 8, 8 | 2 | NA | 1, 1 | 1, 1 | 1, 1 |
| SE | Biomass Outside NTZ | 2, 2 | 6, 6 | 2 | 4, 4 | 3 | 2, 2 | 5, 5 |
| SL | Catch Trend 1 Year | 129, 129 | NA | NA | NA | NA | 153, 156 | 117, 119 |
| SL | Catch Trend 5 Years | 57, 90 | NA | NA | NA | NA | 100, 112 | 87, 95 |
| SL | Food Security | 129, 129 | NA | NA | NA | NA | 153, 156 | 117, 119 |
| SL | Household Assets | 129, 129 | NA | NA | NA | NA | 153, 156 | 117, 119 |
| SL | Livelihood Stability | 129, 129 | NA | NA | NA | NA | 153, 156 | 117, 119 |
| SL | Political Trust | 129, 129 | NA | NA | NA | NA | 153, 156 | 117, 119 |
| SL | Social Trust | 129, 129 | NA | NA | NA | NA | 153, 156 | 117, 119 |
| SL | Subjective Well-being | 129, 129 | NA | NA | NA | NA | 153, 156 | 117, 119 |

Table S6: Sample size for each indicator collected at the first seven Philippines sites. Sample size is the number of individual survey respondents or the number of underwater visual survey sampling locations, with text denoted as “Sample size before intervention at intervention site, Sample size after intervention at intervention site; Sample size before intervention at control site, Sample size after intervention at control site.” Survey acronyms are CS (Community Support), SFP (Sustainable Fishing Practices), SE (Sustainable Ecosystems), and SL (Sustainable Livelihoods).

| **Survey** | **Indicator** | **AYUNGON** | **BINDOY_** | **CANTILA** | **CORTES_** | **CULASI_** | **DAPACOR** | **DCARMEN** |
| --- | --- | --- | --- | --- | --- | --- | --- | --- |
| CS | Attitude | 410, 125 | NA | NA | NA | 220, 221 | 100, 99 | 283, 281 |
| CS | Communication | NA | NA | NA | NA | 219, 220 | 98, 97 | 280, 277 |
| CS | Knowledge | 410, 125 | NA | NA | NA | 220, 221 | 99, 99 | 282, 280 |
| SFP | Catch Reporting | 392, 124 | NA | NA | NA | 220, 221 | 100, 100 | 279, 278 |
| SFP | Licensing | 390, 125 | NA | NA | NA | 220, 220 | 96, 98 | 277, 280 |
| SFP | Management Participation | 403, 125 | NA | NA | NA | 220, 221 | 100, 100 | 283, 281 |
| SFP | NTZ Compliance | NA | NA | NA | NA | 219, 220 | 95, 97 | 263, 275 |
| SFP | TURF Compliance | NA | NA | NA | NA | 201, 213 | 95, 95 | 267, 252 |
| SE | Biomass Inside NTZ | 3, 3 | 3, 2 | 3, 3 | 7, 7 | 2, 1; 1, 1 | 3, 3; 3, 3 | 3, 4 |
| SE | Biomass Outside NTZ | 3, 2 | 3, 2 | 6, 6 | 4, 3 | 1, 1; 2, 2 | 3, 3; 3, 3 | 3, 2 |
| SL | Collective Efficacy | NA | 100, 100 | NA | NA | 100, 100; 97, 100 | 100, 100; 100, 100 | NA |
| SL | Food Security | 100, 100 | 100, 100 | NA | NA | 100, 100; 97, 100 | 100, 100; 100, 100 | NA |
| SL | Livelihood Stability | 100, 100 | 100, 100 | NA | NA | 100, 100; 97, 100 | 100, 100; 100, 100 | NA |
| SL | Political Trust | 100, 100 | 100, 100 | NA | NA | 100, 100; 97, 100 | 100, 100; 100, 100 | NA |
| SL | Social Equity | 100, 100 | 100, 100 | NA | NA | 100, 100; 97, 100 | 100, 100; 100, 100 | NA |
| SL | Social Trust | 100, 100 | 100, 100 | NA | NA | 100, 100; 97, 100 | 100, 100; 100, 100 | NA |
| SL | Subjective Well-being | 100, 100 | 100, 100 | NA | NA | 100, 100; 97, 100 | 100, 100; 100, 100 | NA |

Table S7: Sample size for each indicator collected at the next seven Philippines sites. Sample size is the number of individual survey respondents or the number of underwater visual survey sampling locations, with text denoted as “Sample size before intervention at intervention site, Sample size after intervention at intervention site; Sample size before intervention at control site, Sample size after intervention at control site.” Survey acronyms are CS (Community Support), SFP (Sustainable Fishing Practices), SE (Sustainable Ecosystems), and SL (Sustainable Livelihoods).

| **Survey** | **Indicator** | **GUBATRA** | **INABANG** | **IPILBUL** | **LIBERTA** | **LOOCBAH** | **LUBANG** | **LUBANG_** |
| --- | --- | --- | --- | --- | --- | --- | --- | --- |
| CS | Attitude | 295, 295 | NA | NA | 100, 100 | 167, 168 | NA | 194, 132 |
| CS | Communication | 275, 294 | NA | NA | 100, 100 | 166, 165 | NA | 193, 131 |
| CS | Knowledge | 293, 295 | NA | NA | 100, 100 | 167, 168 | NA | 192, 130 |
| SFP | Catch Reporting | 295, 294 | NA | NA | 92, 100 | 165, 166 | NA | 194, 132 |
| SFP | Licensing | 293, 289 | NA | NA | 98, 100 | 165, 165 | NA | 193, 132 |
| SFP | Management Participation | 295, 295 | NA | NA | 100, 100 | 167, 169 | NA | 194, 132 |
| SFP | NTZ Compliance | 290, 290 | NA | NA | 100, 99 | 166, 165 | NA | 192, 128 |
| SFP | TURF Compliance | 279, 288 | NA | NA | 88, 96 | 140, 162 | NA | 158, 127 |
| SE | Biomass Inside NTZ | 3, 3; 3, 3 | 3 | 3, 3 | 3, 3 | 3, 3 | 3, 3 | NA |
| SE | Biomass Outside NTZ | 2, 2; 3, 3 | 6 | 3, 3 | 3, 3 | 3, 3 | 3, 3 | NA |
| SL | Collective Efficacy | 100, 100; 99, 100 | NA | NA | NA | NA | NA | NA |
| SL | Food Security | 100, 100; 99, 100 | NA | NA | NA | NA | NA | NA |
| SL | Livelihood Stability | 100, 100; 99, 100 | NA | NA | NA | NA | NA | NA |
| SL | Political Trust | 100, 100; 99, 100 | NA | NA | NA | NA | NA | NA |
| SL | Social Equity | 100, 100; 99, 100 | NA | NA | NA | NA | NA | NA |
| SL | Social Trust | 100, 100; 99, 100 | NA | NA | NA | NA | NA | NA |
| SL | Subjective Well-being | 100, 100; 99, 100 | NA | NA | NA | NA | NA | NA |

Table S8: Sample size for each indicator collected at the final seven Philippines sites. Sample size is the number of individual survey respondents or the number of underwater visual survey sampling locations, with text denoted as “Sample size before intervention at intervention site, Sample size after intervention at intervention site; Sample size before intervention at control site, Sample size after intervention at control site.” Survey acronyms are CS (Community Support), SFP (Sustainable Fishing Practices), SE (Sustainable Ecosystems), and SL (Sustainable Livelihoods).

| **Survey** | **Indicator** | **MANJUYO** | **MASINLO** | **MERCEDE** | **SAGNAY_** | **SCARLOS** | **TAYASAN** | **TINAMBAC** |
| --- | --- | --- | --- | --- | --- | --- | --- | --- |
| CS | Attitude | 251, 249 | 104, 94 | 252, 252 | NA | 101, 100 | 307, 309 | NA |
| CS | Communication | 233, 248 | 103, 91 | 252, 251 | NA | 99, 100 | 305, 307 | NA |
| CS | Knowledge | 219, 231 | 104, 94 | 252, 252 | NA | 101, 100 | 306, 309 | NA |
| SFP | Catch Reporting | 248, 245 | 104, 90 | 252, 250 | NA | 101, 100 | 306, 308 | NA |
| SFP | Licensing | 247, 243 | 104, 89 | 252, 250 | NA | 98, 100 | 305, 307 | NA |
| SFP | Management Participation | 250, 249 | 104, 94 | 252, 252 | NA | 101, 100 | 307, 309 | NA |
| SFP | NTZ Compliance | 247, 238 | 103, 85 | 252, 250 | NA | 101, 100 | 304, 304 | NA |
| SFP | TURF Compliance | 227, 245 | 97, 86 | 231, 247 | NA | 92, 100 | 277, 285 | NA |
| SE | Biomass Inside NTZ | 3, 3 | 3, 3 | 3, 3 | 3, 3 | 3, 3 | 2, 2 | 5, 5 |
| SE | Biomass Outside NTZ | 3, 2 | 3, 2 | 3, 3 | 3, 2 | 2, 2 | 2, 2 | 6, 6 |
| SL | Collective Efficacy | 52, 100 | NA | NA | NA | NA | NA | NA |
| SL | Food Security | 52, 100 | NA | NA | NA | NA | NA | NA |
| SL | Livelihood Stability | 52, 100 | NA | NA | NA | NA | NA | NA |
| SL | Political Trust | 52, 100 | NA | NA | NA | NA | NA | NA |
| SL | Social Equity | 52, 100 | NA | NA | NA | NA | NA | NA |
| SL | Social Trust | 52, 100 | NA | NA | NA | NA | NA | NA |
| SL | Subjective Well-being | 52, 100 | NA | NA | NA | NA | NA | NA |

Table S10: Target fished families included in the Philippines and Indonesia underwater visual ecological surveys used to determine target family biomass inside and outside a site’s no-take zone.

| Philippines | | | | |
| --- | --- | --- | --- | --- |
| Acanthuridae | Diodontidae | Labridae | Pomacanthidae | Sphyraenidae |
| Balistidae | Ephippidae | Lethrinidae | Priacanthidae | Synodontidae |
| Caesionidae | Fistulariidae | Lutjanidae | Scaridae |  |
| Carangidae | Haemulidae | Mullidae | Scombridae |  |
| Chanidae | Holocentridae | Nemipteridae | Serranidae |  |
| Clupeidae | Kyphosidae | Plotosidae | Siganidae |  |
| Indonesia | | | | |
| Acanthuridae | Carangidae | Labridae | Lutjanidae | Serranidae |
| Caesionidae | Haemulidae | Lethrinidae | Scaridae | Siganidae |
