# Supplementary material for: Catalyzing sustainable fisheries management through behavior change interventions
Source: Conserv Biol. 2020 Apr 15;34(5):1176–89. doi: 10.1111/cobi.13475 (PMC7540413; doi:10.1111/cobi.13475)
Supplement: Supplementary file 4 — Supplementary Material [file COBI-34-1176-s004.docx]

|  |  |  |  |  |  |  |  |  |  |  |
| --- | --- | --- | --- | --- | --- | --- | --- | --- | --- | --- |
|  | 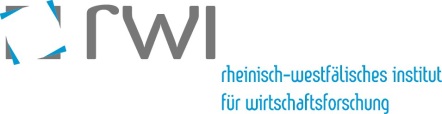**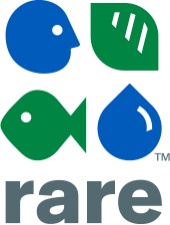** | |  |  |  | Number of Questionnaire | | |  |  |
|  |  |  |  |  |  |  |  |  |  |  |
|  |  |  |  |  |  | Name of the region | | |  |  |
|  |  |  |  |  |  |  | | |  |  |
|  | **Household Questionnaire**  Impact Evaluation 2017, Indonesia | | | |  | Name of the Village | | |  |  |
|  |  |  |  |  |  |  |  |  |  |  |
|  |  |  |  |  |  |  | | |  |  |

|  |  |  |  |  |  |  |  |  |  | |  |  |  |  |
| --- | --- | --- | --- | --- | --- | --- | --- | --- | --- | --- | --- | --- | --- | --- |
|  |  | Name of Enumerator | | |  | | | | | Respondent name | | |  |  |
|  |  |  |  |  |  |  |  |  |  |  |  |  |  |  |
|  |  |  | | |  | | | | | | | |  |  |
|  |  | Group Identifier | | | 1. Treatment | a.original | |  | Date of the interview | | | |  |  |
|  |  |  |  |  |  |  |  |  |  |  |  |  |  |  |
|  |  |  | | | 1. Control | b.replacement | |  | Time of interview | | | |  |  |

1. **Household (Composition)**

|  | |  | | | |  | | | | |  | |  | | | | |  | |  |  |  |  | | | | | | | |  | |  | |  | |  | | |  | | | | | |
| --- | --- | --- | --- | --- | --- | --- | --- | --- | --- | --- | --- | --- | --- | --- | --- | --- | --- | --- | --- | --- | --- | --- | --- | --- | --- | --- | --- | --- | --- | --- | --- | --- | --- | --- | --- | --- | --- | --- | --- | --- | --- | --- | --- | --- | --- |
|  | |  | | | | The main building **[of head of household]** has walls made out of | | | | | | | | | | | |  | |  | The roof is made out of … | | | | | | | | | |  |  | | The floor is made of… | | | | | | | | | | |  |
|  | |  | | | |  |  |  |  |  |  |  |  |  |  |  |  |  | |  |  | | | | | | | | | |  |  | |  | | | | | | | | | | |  |
|  | |  | |  | | |  | | |  | | | | | | | | | | | | | | | | | | | | | | | | | | | | | | | | | |  | |
|  | |  | | | | 1 | | | | | 🞎 | | Bamboo | | | | |  | |  | 1 | 🞎 | Thatch / leaves | | | | | | | |  | |  | | 1 | | 🞎 | | | Dirt / Soil | | | | | |
|  | |  | | | | 2 | | | | | 🞎 | | Wood | | | | |  | |  | 2 | 🞎 | Tile | | | | | | | |  | |  | | 2 | | 🞎 | | | Bamboo / Palm | | | | | |
|  | |  | | | | 3 | | | | | 🞎 | | Brick / Cement | | | | |  | |  | 3 | 🞎 | CorrugatedIron | | | | | | | |  | |  | | 3 | | 🞎 | | | Plank Wood | | | | | |
|  | |  | | | | 4 | | | | | 🞎 | | CorrugatedIron | | | | |  | |  | 4 | 🞎 | Concrete | | | | | | | |  | |  | | 4 | | 🞎 | | | Cement | | | | | |
|  | |  | | | | 5 | | | | | 🞎 | | Other: | | | | |  | |  | 5 | 🞎 | Asbestos | | | | | | | |  | |  | | 5 | | 🞎 | | | Finished (tiles, etc.) | | | | | |
|  | |  | | | |  | | | | |  | |  | | | | |  | |  | Other: | | | | | | | | | |  | |  | | 6 | | 🞎 | | | Other: | | | | | |
|  | |  | | | |  | | | | |  | |  |  |  |  |  |  | |  |  |  |  |  |  |  |  |  |  |  |  | |  | |  | |  | | |  | | | | | |
|  | |  | | | |  | | | | |  | |  | | | | |  | |  |  |  |  | | | | | | | |  | |  | |  | |  | | |  | | | | | |
|  | |  | | |  | | | |  | | | | | |  |  |  | | |  | | | |  | |  | | |  |  | | | | | | |  | |  | |  |  |  | | |
|  | |  | | | | |  | | | |  | | | | | | |  | | | | |  |  | |  |  | | | | | | | |  | |  | | | | | | | |  |
|  | | **10.** | | | | | **Who is the interviewed person?** | | | | | | | | | | |  | | | | |  |  | |  | **CODE Q. 20**  0. None  1. Elementary School  2. Intermediary School  3. High School  4. University  5. Reading / Writing  6. Koran School  **CODE Q.12**  1. head of household  2. spouse of head of household  3. father / mother  4. brother / sister  5. son / daughter  6. grandson / granddaughter  7. other parent  8. maid / boy  9. no family ties  10. son/daughter in law | | | | | | | |  | | **CODE Q.16 and 17**  1. Fishermen (**independent**)  2. Farmer (**independent**)  3. Government Employee  4. Other independent  activity [**specify**]  5. Fishermen **(dependent)**  6. Farmer **(dependent)**  7. Other dependent activity **[specify]**  8. Family help  9. Pupil, student  10. Household chores, retired, invalid  11. Unemployed | | | | | | | |  |
|  | |  | | | | |  |  |  |  |  |  |  |  |  |  |  | **Huruf di Letter of Q. 12** | | | | |  |  | |  |  |  |  |  |  |  |  |  |  | |  |  |  |  |  |  |  |  |  |
|  |  |  | | | | |  | | | | | | | | | | |  | | | | |  |  |  |  |  |  |  |  |  |  |  |  |  |  |  |  |  |  |  |  |  |  |  |
|  | | **11.** | | | | | How many children 5 years or younger live in the household? | | | | | | | | | | | **__________** | | | | |  |  |  |  |  |  |  |  |  |  |  |  |  |  |  |  |  |  |  |  |  |  |  |
|  | |  | | | | |  |  |  |  |  |  |  |  |  |  |  |  |  |  |  |  |  |  |  |  |  |  |  |  |  |  |  |  |  |  |  |  |  |  |  |  |  |  |  |
|  | |  | | | | | | | | | | |  | | | | |  | | | | |  |  |  |  |  |  |  |  |  |  |  |  |  | |  |  |  |  |  |  |  |  |  |
|  |  | **[COMMENT]** | | | | | | | | | | | | | | | | | | | | |  |  |  |  |  |  |  |  |  |  |  |  |  |  |  |  |  |  |  |  |  |  |  |
|  |  |  | | | | | | | | | | | | | | | | | | | | |  |  |  |  |  |  |  |  |  |  |  |  |  |  |  |  |  |  |  |  |  |  |  |

| **12.** | |  | | | **13.** |  | | **14.** | |  | **15.** |  | **16...** |  | **17..** |  | **18.** |  | **19.** |  | **20.** |  | |  |  |  |  |  |  |  |  |  |  |
| --- | --- | --- | --- | --- | --- | --- | --- | --- | --- | --- | --- | --- | --- | --- | --- | --- | --- | --- | --- | --- | --- | --- | --- | --- | --- | --- | --- | --- | --- | --- | --- | --- | --- |
|  | | | | |  | |  | |  | | | |  | |  | |  | |  | |  | |  |  |  |  |  |  |  |  |  |  |  |
| Including yourself, please give the names of all the people older than five years who currently live in your household and their relationship to you? | | | | Sex | | Age | | Has she borne a child in the last 12 month?  **(only if**  **q. 13=female)** | | | |  |  |  |  |  |  |  |  |  |  |  | First Occupation | | Second Occupation | | What are his / her gross earnings per month? | | Level of obtained education? | | Number of years in education? | | |
|  | | | | |  | |  | |  |  |  |  |  |  |  |  |  |  |  |  |  |  |  |  |  |  |  |  |  |  |  |  |  |
| *code* | | | | *Name* | *m / f* | | *years* | | *1. Yes 0. No* | | | | *code* | | *code* | | *Rupiah* | | *code* | |  | | |  |  |  |  |  |  |  |  |  |  |
| a.  | | | 1 |  |  | |  | |  | | | |  | |  | |  | |  | |  | | |  |  |  |  |  |  |  |  |  |  |
| b.  | | |  |  |  | |  | |  | | | |  | |  | |  | |  | |  | | |  |  |  |  |  |  |  |  |  |  |
| c.  | | |  |  |  | |  | |  | | | |  | |  | |  | |  | |  | | |  |  |  |  |  |  |  |  |  |  |
| d.  | | |  |  |  | |  | |  | | | |  | |  | |  | |  | |  | | |  |  |  |  |  |  |  |  |  |  |
| e.  | | |  |  |  | |  | |  | | | |  | |  | |  | |  | |  | | |  |  |  |  |  |  |  |  |  |  |
| f.  | | |  |  |  | |  | |  | | | |  | |  | |  | |  | |  | | |  |  |  |  |  |  |  |  |  |  |
| g.  | | |  |  |  | |  | |  | | | |  | |  | |  | |  | |  | | |  |  |  |  |  |  |  |  |  |  |
| h.  | | |  |  |  | |  | |  | | | |  | |  | |  | |  | |  | | |  |  |  |  |  |  |  |  |  |  |
| i.  | | |  |  |  | |  | |  | | | |  | |  | |  | |  | |  | | |  |  |  |  |  |  |  |  |  |  |
| j.  | | |  |  |  | |  | |  | | | |  | |  | |  | |  | |  | | |  |  |  |  |  |  |  |  |  |  |
| k.  | | |  |  |  | |  | |  | | | |  | |  | |  | |  | |  | | |  |  |  |  |  |  |  |  |  |  |
| l.  | | |  |  |  | |  | |  | | | |  | |  | |  | |  | |  | | |  |  |  |  |  |  |  |  |  |  |

|  | **21.** | | |  | | |  | |  | |  |  | |  | **22.** | |  | | | | | | |  |  |  |
| --- | --- | --- | --- | --- | --- | --- | --- | --- | --- | --- | --- | --- | --- | --- | --- | --- | --- | --- | --- | --- | --- | --- | --- | --- | --- | --- |
|  | What is the ethnicity of the head of household? **(S/M)** | | | | | | | | | | | |  | | What is the religion of the head of household? | | | | | | | |  |  |  |  |
|  |  |  |  | |  |  | |  | |  | | |  | |  |  | |  |  |  |  | | |  |  |  |
|  | 1 | 🞎 | Buton | |  | 2 | | 🞎 | | Bugis | | |  | | 1 | 🞎 | | Muslim |  | 2 | 🞎 | Hindu | |  |  |  |
|  | 3 | 🞎 | Badjo | |  | 4 | | 🞎 | | Pulo | | |  | | 3 | 🞎 | | Traditional believer |  | 4 | 🞎 | Buddhist | |  |  |  |
|  | 5 | 🞎 | Sangir | |  | 6 | |  | | Sunda | | |  | | 5 | 🞎 | | Christian |  | 6 | 🞎 | Other: | |  |  |  |
|  |  |  |  |  |  |  |  |  |  |  |  |  |  |  |  |  |  |  |  |  |  |  | |  |  |  |
|  |  |  |  |  |  |  |  |  |  |  |  |  |  |  |  |  |  |  |  |  | | | |  |  |  |
|  | 7 |  | Jawa | |  | 8 | |  | | Other | | |  | |  |  | |  |  |  | | | | |  |  |
|  |  |  |  | |  |  | |  | |  | | |  | |  |  | |  |  |  | | | | |  |  |

1. **Financial Situation, Asset Index and Consumption Levels**

|  |  |  |  |  |  |  |  |  |  |  |  | |  | | | | |  |  | | | |  | | |  |
| --- | --- | --- | --- | --- | --- | --- | --- | --- | --- | --- | --- | --- | --- | --- | --- | --- | --- | --- | --- | --- | --- | --- | --- | --- | --- | --- |
|  | **23.** | |  |  |  | **24.** | |  |  |  | **25.** | |  | | | | | **26.** | | |  | | | | |  |
|  | Do you or any of this household member have an account at a bank or savings association? | | | |  | Do you or any of this household member save money elsewhere? | | | |  | Did the household take up a loan during the last two years? | | | | | | | Where? **[SEVERAL ANSWERS POSSIBLE]** | | | | | | |  | |
|  |  |  |  |  |  |  |  |  |  |  |  |  |  |  |  |  |  | 1. Family 2. friends 3. shops 4. financial institution (informal loan) 5. bank 6. others, specify | | | | | | |  | |
|  | 1 | 🞎 | Yes, at a bank | |  | 1 | 🞎 | Yes | |  |  | | | | | | |  |  |  |  |  |  |  |  | |
|  | 2 | 🞎 | Yes, at a savings association | |  | 2 | 🞎 | No | |  |  | | | | | | |  |  |  |  |  |  |  |  | |
|  |  |  |  |  |  |  |  |  | |  |  |  |  | | | | |  |  |  |  |  |  |  |  | |
|  |  |  |  |  |  |  |  |  |  |  | 1 | 🞎 | Yes | | | | |  | | | | | | |  | |
|  | 0 | 🞎 | No | |  |  |  |  |  |  | 0 | 🞎 | No🠆**q. 27** | | | | | ___________ | | | | | | |  | |
|  |  |  |  | |  |  |  |  |  |  |  |  |  | | | | |  | | | | | | |  | |
|  | **27**. | |  | |  | **28.** | |  |  |  |  |  |  | | | | |  | | | | | | |  | |
|  | If needed, could you get a credit at a bank? | | | |  | If you needed to borrow a week’s worth of money, is it easy to get/borrow the money to the people beyond your immediate household and relatives? | | | | | | | | | | | | | | | | | | |  | |
|  |  |  |  |  |  |  |  |  |  |  |  |  |  |  |  |  |  |  |  |  |  |  |  |  |  | |
|  |  |  |  | |  |  |  |  |  |  |  |  |  | | | | |  | | | | | | |  | |
|  | 1 | 🞎 | Yes | |  | 1 | 🞎 | Very easy | | |  | 4 | 🞎 | Quite Difficult |  | |  |  | | | | | | |  | |
|  | 2 | 🞎 | No |  |  | 2 | 🞎 | Quite easy | | |  | 5 | 🞎 | Very Difficult | |  | | | |  | |  | |  |  | |
|  |  |  |  |  |  | 3 | 🞎 | Unsure | | |  |  |  | | | | |  | | | | | | |  | |
|  |  |  |  |  |  |  |  |  |  |  |  |  |  | | | | |  | | | | | | |  | |

|  |  |  |  |  |  |  |  |  |  |  |  |  |  |  |
| --- | --- | --- | --- | --- | --- | --- | --- | --- | --- | --- | --- | --- | --- | --- |
|  | **29.** | |  |  | **30.** | |  |  |  |  | **[COMMENTS]** | |  |  |
|  | How many remittances do you or any of this household member receive per month? | | |  | To cover family needs your household income is… | | |  |  |  |  |  |  |  |
|  |  |  |  |  |  |  |  |  |  |  |  |  |  |  |
|  |  |  |  |  | 1 | 🞎 | sufficient |  |  |  |  |  |  |  |
|  |  |  |  |  | 2 | 🞎 | tight |  |  |  |  |  |  |  |
|  |  |  | IDR |  | 3 | 🞎 | Not sufficient |  |  |  |  |  |  |  |
|  |  |  |  |  |  |  |  |  |  |  |  |  |  |  |

|  |  | |  | | |  | | | | |  | |
| --- | --- | --- | --- | --- | --- | --- | --- | --- | --- | --- | --- | --- |
|  | **31.** | | |  | | | | | | |  | |
|  | Where is your toilet / sanitary facilities | | | | | | | | | | |  |
|  |  |  | | |  | |  |  |  |  | |  |
|  | 0 | 🞎 | | | Domestic latrine with protected septic tank | |  | 1 | 🞎 | Public latrine | |  |
|  | 2 | 🞎 | | | Domestic latrine with waste canal to fishpond / drain | |  | 3 |  | River | |  |
|  | 4 | 🞎 | | | Open land/yard | |  | 5 |  | Other, specify: _______ | |  |
|  |  |  | | |  | |  |  |  |  | |  |
|  |  | |  | | |  | | | | |  | |
|  | **32.** | | |  | | | | | | |  | |
|  | What is the ownership status of your house? | | | | | | | | | | |  |
|  |  |  | | |  | |  |  |  |  | |  |
|  | 0 | 🞎 | | | Rented | |  | 1 | 🞎 | Your own house | |  |
|  | 2 | 🞎 | | | Family’s house (occupied for free) | |  |  |  |  | |  |
|  |  |  | | |  | |  |  |  |  | |  |

|  |  | |  | | |  | | | | | | | | | |  | |  |  | |
| --- | --- | --- | --- | --- | --- | --- | --- | --- | --- | --- | --- | --- | --- | --- | --- | --- | --- | --- | --- | --- |
|  | **33.** | | |  | | | | | | | | | | | |  | |  |  | |
|  | Which of the following vehicles do you own? If so, how many? | | | | | | | | | | | | | | | | | | |  |
|  |  |  | | |  | |  |  |  |  | | | | | | | | | |  |
|  | 0 | 🞎 | | | None | |  | 3 | 🞎 | Bicycle | | | | | | | | | |  |
|  | 1 | 🞎 | | | Motorbike or  Scooter | |  | 4 | 🞎 | Car | | | | | | | | | |  |
|  | 2 | 🞎 | | | Non-powered Boat | |  | 5 | 🞎 | Outboard powered motorboat | | | | | | | | | |  |
|  | 6 | 🞎 | | | Other: ________ | |  |  |  |  | | | | | | | | | |  |
|  |  |  | | |  | |  |  |  |  | | | | | | | | | |  |
|  |  |  | | |  | |  |  |  |  | | | | | | | | | |  |
|  |  |  | | |  | |  |  |  |  | | | | | | | | | |  |
|  | **34.** | | | |  | |  |  |  |  | | | | | | | | | |  |
|  | Do you rent a boat to go fishing or share one with others? If yes, how often do you rent it and how much do you pay? | | | | | | | | | | | | | | | | | | |  |
|  |  |  | | |  | |  | | |  | | | | | | | | | |  |
|  | 1 | 🞎 | | | Yes | |  | ____________ **IDR / Trip** | | | | | | | ______ **Trip / month** | | | | | |
|  | 2 | 🞎 | | | No | |  |  |  |  | |  | | |  | |  | | |  |
|  | 3 |  | | | Production/profit sharing | |  | Your proportion: ____% | | | | | | |  | | | | | |
|  |  |  | | |  | |  |  |  |  | | | | | | | | | |  |
|  |  |  | | |  | |  |  |  |  | | | | | | | | | |  |
|  | **35.** | | |  | | |  |  |  |  | | | | | | | | | |  |
|  | Which of the following items do you own? If so, how many? | | | | | | | | | | | | | | | | | | |  |
|  |  | |  | | |  | | |  | |  | |  |  | | | | | |  |
|  | 0 | | 🞎 | | | None | | |  | | 1 | | 🞎 | Television | | | | | |  |
|  | 2 | | 🞎 | | | Refrigerator | | |  | | 3 | | 🞎 | Mobile Phone | | | | | |  |
|  | 4 | | 🞎 | | | Satellite Dish | | |  | | 5 | | 🞎 | Radio | | | | | |  |
|  | 6 | | 🞎 | | | Computer / Laptop | | |  | | 7 | | 🞎 | Other: | | | | | |  |
|  |  | |  | | |  | | |  | |  | |  |  | | | | | |  |

1. **Fishery Section**

|  |  |  |  |  |  | |  | |  | |  |  |
| --- | --- | --- | --- | --- | --- | --- | --- | --- | --- | --- | --- | --- |
|  | **36.** | Does someone in the household go fishing, collect sea cucumbers, sea urchins, or mines coral? |  |  | 1 | | 🞎 | | Yes | |  |  |
|  |  |  |  |  | | 0 | 🞎 | No | | **🠆q. 54** | | |
|  |  |  |  |  |  | |  | |  | |  |  |

|  |  | |  |  |  |  |  | | | | |  |  |
| --- | --- | --- | --- | --- | --- | --- | --- | --- | --- | --- | --- | --- | --- |
| **37.** | |  | | | | |  | | | | |  |  |
| In the last 12 months…… | | | | | | |  | | | | |  |  |
| How often did someone in your household go fishing? | | | | | | | Once or never | A few times | A few times per month | 1-2 times per week | More than 1-2 times per week |  |  |
| How often did someone in your household go fishing in the less productive season? | | | | | | | Once or never | A few times | A few times per month | 1-2 times per week | More than 1-2 times per week |  |  |
| How often did someone in your household go fishing in the more productive season? | | | | | | | Once or never | A few times | A few times per month | 1-2 times per week | More than 1-2 times per week |  |  |
| How often did someone in your household collect invertebrates or coral? | | | | | | | Once or never | A few times | A few times per month | 1-2 times per week | More than 1-2 times per week |  |  |

1. a. At what specific month(s) you go for fishing?
2. At what specific month(s) as the more productive season for fishing?
3. At what specific month(s) as less productive season for fishing

|  | Jan | Feb | Mar | Apr | Mei | Jun | Jul | Aug | Sep | Okt | Nov | Des |
| --- | --- | --- | --- | --- | --- | --- | --- | --- | --- | --- | --- | --- |
| 1. Go for fishing | 1 | 2 | 3 | 4 | 5 | 6 | 7 | 8 | 9 | 10 | 11 | 12 |
| b.More productive season | 1 | 2 | 3 | 4 | 5 | 6 | 7 | 8 | 9 | 10 | 11 | 12 |
| c.Less productive season | 1 | 2 | 3 | 4 | 5 | 6 | 7 | 8 | 9 | 10 | 11 | 12 |

|  | **39.** | | |  | **40.** |  | **41.** |  | | **42.** |  | | **43.** |  |  |  | | **44.** |  | **45** | |  | **46.** |  | |
| --- | --- | --- | --- | --- | --- | --- | --- | --- | --- | --- | --- | --- | --- | --- | --- | --- | --- | --- | --- | --- | --- | --- | --- | --- | --- |
|  | Which fish species did you fish in the last 12 months?  Snapper  Grouper  Rabbitfish  Tuna  Octopus  Emperor | | | | Rank the fish species by importance for you (income) | | Do you sell these fish?  **If No: q. 46** | | Where do you sell these fish?  0 Local market  1 Export  2 Other (specify) | | | For how much did you sell all of these fish within the last 12 months? | | | | | For how much did you sell the processed fish or its byproduct in the last 12 month? | | | | How much was the catch worth you consumed yourself in the last 12 month? | | What fishing gear did you use to catch the fish? | |  |
|  |  | | | |  | |  | |  | | | *Rupiah* | | | | |  | | | | *Rupiah* | | *Code Q.46* | |  |
| **1.** | 🞎 | |  | |  | | 0 🞎 No 1 🞎 Yes | |  | | |  | | | | |  | | | |  | |  | |  |
| **2.** | 🞎 | |  | |  | | 0 🞎 No 1 🞎 Yes | |  | | |  | | | | |  | | | |  | |  | |  |
| **3.** | 🞎 | |  | |  | | 0 🞎 No 1 🞎 Yes | |  | | |  | | | | |  | | | |  | |  | |  |
| **4.** | 🞎 | |  | |  | | 0 🞎 No 1 🞎 Yes | |  | | |  | | | | |  | | | |  | |  | |  |
| **5.** | 🞎 | |  | |  | | 0 🞎 No 1 🞎 Yes | |  | | |  | | | | |  | | | |  | |  | |  |
| **6.** | 🞎 | |  | |  | | 0 🞎 No 1 🞎 Yes | |  | | |  | | | | |  | | | |  | |  | |  |
| **7.** | 🞎 | |  | |  | | 0 🞎 No 1 🞎 Yes | |  | | |  | | | | |  | | | |  | |  | |  |
| **8.** | 🞎 | |  | |  | | 0 🞎 No 1 🞎 Yes | |  | | |  | | | | |  | | | |  | |  | |  |
| **9.** | 🞎 |  | | |  | | 0 🞎 No 1 🞎 Yes | |  | | |  | | | | |  | | | |  | |  | |  |
| **10.** | 🞎 | |  | |  | | 0 🞎 No 1 🞎 Yes | |  | | |  | | | | |  | | | |  | |  | |  |
| **11.** | 🞎 | |  | |  | | 0 🞎 No 1 🞎 Yes | |  | | |  | | | | |  | | | |  | |  | |  |
| **12.** | 🞎 | |  | |  | | 0 🞎 No 1 🞎 Yes | |  | | |  | | | | |  | | | |  | |  | |  |
| **13.** | 🞎 | | Other _______ | |  | | 0 🞎 No 1 🞎 Yes | |  | | |  | | | | |  | | | |  | |  | |  |
|  |  | |  | |  | |  | |  | | |  | | | | |  | | | |  | |  | |  |

|  |  | |  | | |  | | | | |  | |
| --- | --- | --- | --- | --- | --- | --- | --- | --- | --- | --- | --- | --- |
|  | **47.** | | |  | | | | | | |  | |
|  | In the last 12 months what fishing gear has the fisher used?  **Rank the gears used depending on their usage** | | | | | | | | | | |  |
|  |  | **Gear** | | | | | **Used?** | | | **Rank** | |  |
|  | 0 | No fishing in last 12 month | | | | | 0 🞎No 1 🞎Yes | | |  | |  |
|  | 1 | Fishing by hand (e.g. gleaning) | | | | | 0 🞎No 1 🞎Yes | | |  | |  |
|  | 2 | Fishing by handheld gun (e.g. speargun) | | | | | 0 🞎 No 1 🞎 Yes | | |  | |  |
|  | 3 | Fishing with stationery net (e.g. gill net, trammel net) | | | | | 0 🞎 No 1 🞎 Yes | | |  | |  |
|  | 4 | Fishing with mobile net (e.g. trawl, purse seine, beach seine) | | | | | 0 🞎 No 1 🞎 Yes | | |  | |  |
|  | 5 | Fishing with floating net (bagan) | | | | |  | | |  | |  |
|  | 6 | Fishing with stationary line (e.g. long line) | | | | | 0 🞎 No 1 🞎 Yes | | |  | |  |
|  | 7 | Fishing with mobile line (e.g. trolling, hand line) | | | | | 0 🞎 No 1 🞎 Yes | | |  | |  |
|  | 8 | Fishing with explosives (e.g. bomb) | | | | | 0 🞎 No 1 🞎 Yes | | |  | |  |
|  | 9 | Fishing with chemicals (e.g. cyanide, poison) | | | | | 0 🞎 No 1 🞎 Yes | | |  | |  |
|  | 10 | Dive Fishing (compressor) | | | | | 0 🞎 No 1 🞎 Yes | | |  | |  |
|  | 11 | Trap Fishing (bubu, sero) | | | | | 0 🞎 No 1 🞎 Yes | | |  | |  |
|  | 12 | Other: _____________ | | | | | 0 🞎 No 1 🞎 Yes | | |  | |  |
|  |  |  | | |  | |  |  |  |  | |  |
|  |  |  | | |  | |  |  |  |  | |  |

|  |  |  |  |  | | | |  | |  | |
| --- | --- | --- | --- | --- | --- | --- | --- | --- | --- | --- | --- |
|  | **48.** | Do you sell any other marine product (for example: seaweed, agar agar, coral rock sand, “coral ornaments”)? |  | 1 | 🞎 | Yes |  | |  | |  |
|  |  |  |  | 0 | 🞎 | No | **q. 51** | |  |  |  |
|  |  |  |  |  | | | |  | |  | |

|  |  |  |  | 1. ___________  2. ___________  3. ___________ | |  |
| --- | --- | --- | --- | --- | --- | --- |
|  | **49.** | What are those other marine products? |  |  |  |  |
|  |  |  |  |  |  |  |
|  |  |  |  |  |  |  |

|  |  |  |  |  |  |  |  |  |
| --- | --- | --- | --- | --- | --- | --- | --- | --- |
|  | **50.** | How much do you earn by selling any other marine product per year? |  |  |  |  | Rupiah |  |
|  |  |  |  |  |  |  |  |  |
|  |  |  |  |  |  |  |  |  |

| **51.** | |  |  |  |  |  |  |
| --- | --- | --- | --- | --- | --- | --- | --- |
| **The fish catch has …..**  **compared to** | | | Improved heavily | Improved slightly | Stayed the same | Deteriorated slightly | Deteriorated a lot |
|  | 1 year ago | | 🞎 | 🞎 | 🞎 | 🞎 | 🞎 |
| 5 years ago | | | 🞎 | 🞎 | 🞎 | 🞎 | 🞎 |
|  |  |  |  |  |  |  |  |
| 10 years ago | | | 🞎 | 🞎 | 🞎 | 🞎 | 🞎 |
|  |  |  |  |  |  |  |  |

|  |  |  | | |  | | |  | |  |  | |  |  |
| --- | --- | --- | --- | --- | --- | --- | --- | --- | --- | --- | --- | --- | --- | --- |
|  | **52a.** | Have you changed the fishing ground in the last year? | | | |  | | **52b.** | Is the new fishing ground closer or further away? | | | | |  |
|  |  |  |  |  |  |  |  |  |  |  |  |  |  |  |
|  |  |  | | |  | |  | | |  | |  |  |  |
|  |  | | Yes  | | | | | Further away | |  |  | |  |  |
|  |  | | | No**=>q. 52** | | | | Closer | |  |  | |  |  |
|  |  |  | | |  | | |  | |  |  | |  |  |

|  |  |  |  |  | |  |  | |  |  |  |
| --- | --- | --- | --- | --- | --- | --- | --- | --- | --- | --- | --- |
|  | **53.** | Are there any species that were important in the past but are not important anymore? | | | | | | | |  |  |
|  |  |  |  |  |  |  |  |  |  |  |  |
|  |  |  |  | | Names of the species |  | |  |  |  | |
|  | 1 year ago | | Yes 🞎 No 🞎 |  | |  |  | |  |  |  |
|  | 5 years ago | | Yes 🞎 No 🞎 |  | |  |  | |  |  |  |
|  | 10 years ago | | Yes 🞎 No 🞎 |  | |  |  | |  |  |  |
|  |  |  |  |  | |  |  | |  |  |  |

|  |  |  |  |  |  |  |  |  |  |  |  |  |
| --- | --- | --- | --- | --- | --- | --- | --- | --- | --- | --- | --- | --- |
|  | **54.** |  |  | **1.** |  |  |  | **2.** |  |  |  |  |
|  | Please rate your agreement with the following statement on a scale from strongly disagree to strongly agree | | | Strongly Agree | Agree | Neither agree nor disagree | | Disagree | | Strongly Disagree | |  |
|  | Many important things in my life happen by accident | | | 🞎 | 🞎 | 🞎 | | 🞎 | | 🞎 | |  |
|  | The fish abundance is up to god and cannot be influenced by human beings | | | 🞎 | 🞎 | 🞎 | | 🞎 | | 🞎 | |  |
|  | My life is to a large extent controlled by more important people / forces | | | 🞎 | 🞎 | 🞎 | | 🞎 | | 🞎 | |  |
|  | If I work hard enough I will be able to support my family | | | 🞎 | 🞎 | 🞎 | | 🞎 | | 🞎 | |  |
|  | It is mostly luck if I catch a lot of fish on one day but not many on another day | | | 🞎 | 🞎 | 🞎 | | 🞎 | | 🞎 | |  |
|  | Through my individual fishing behavior, I can make a meaningful contribution to the sustainability of the fish catch | | | 🞎 | 🞎 | 🞎 | | 🞎 | | 🞎 | |  |
|  |  | | |  |  |  | |  | |  | |  |

## Social Capital and fishery management

|  |  |  |  | |  |  |  |  |  |  | | | |  |
| --- | --- | --- | --- | --- | --- | --- | --- | --- | --- | --- | --- | --- | --- | --- |
|  |  |  | **55.** | |  | **56.** |  | **57.** |  |  |  |  |  |  |
|  |  |  | Is the head of household or his wife/her husband member in an association? | | | Where/ Which one? | | How many times per month does he / she participate in the organisation? | |  |  |  |  |  |
|  |  |  |  |  |  | 1. Fishing / agricultural association 2. womens’ group 3. political movement/party 4. labour union 5. religious 6. other - which? | |  |  |  |  |  |  |  |
|  | **1.** | Head of household | 1 | 🞎 | Yes |  | |  | |  |  |  |  |  |
|  |  |  | 0 | 🞎 | No**q. 55.2** | ______________ | | ______________ | |  |  |  |  |  |
|  | **2.** | Wife | 1 | 🞎 | Yes |  | |  | |  | |  |  |  |
|  |  |  | 0 | 🞎 | No**q. 58** | ______________ | | ______________ | |  | | |  |  |
|  |  |  |  |  |  |  | |  | |  |  |  |  |  |

|  |  | |  |  |  |  |  |  |  |  |  |  |  |
| --- | --- | --- | --- | --- | --- | --- | --- | --- | --- | --- | --- | --- | --- |
|  | **58.** | |  |  | **1.** |  |  |  | **2.** |  |  |  |  |
|  | Please rate your agreement with the following statement on a scale from strongly disagree to strongly agree | | | | Strongly Agree | Agree | Neither agree nor disagree | | Disagree | | Strongly Disagree | |  |
|  | Generally speaking, most people in my community can be trusted | | | | 🞎 | 🞎 | 🞎 | | 🞎 | | 🞎 | |  |
|  | | In this village, you have to be alert or someone is likely to take advantage of you. | | | 🞎 | 🞎 | 🞎 | | 🞎 | | 🞎 | |  |
|  | Generally speaking, the local government can be trusted to make decisions in the best of our interest | | | | 🞎 | 🞎 | 🞎 | | 🞎 | | 🞎 | |  |
|  | Generally speaking, the religious leaders can be trusted | | | | 🞎 | 🞎 | 🞎 | | 🞎 | | 🞎 | |  |
|  | | Generally speaking, NGO’s can be trusted to make decisions in our best interest | | | 🞎 | 🞎 | 🞎 | | 🞎 | | 🞎 | |  |
|  | Generally speaking, the fishermen from this community can be trusted not to break the rules | | | | 🞎 | 🞎 | 🞎 | | 🞎 | | 🞎 | |  |
|  | Generally speaking, the fishermen from other communities can be trusted not to break the rules | | | | 🞎 | 🞎 | 🞎 | | 🞎 | | 🞎 | |  |
|  |  | | | |  |  |  | |  | |  | |  |

|  |  |  |  |  |  | |  | |  | |  |  |
| --- | --- | --- | --- | --- | --- | --- | --- | --- | --- | --- | --- | --- |
|  | **59.** | Have you ever heard of the XXXX (TURF area)? 🡺 PAAP |  |  | 1 | | 🞎 | | Yes | |  |  |
|  |  |  |  |  | | 0 | 🞎 | No | | **🠆q. 66** | | |
|  |  |  |  |  |  | |  | |  | |  |  |

60. What do you know about PAAP (TURF area)

______________________________________________________________________________________________________________________________________________________

|  |  | |  |  |  |  |  |  |  | |  |  | |  |
| --- | --- | --- | --- | --- | --- | --- | --- | --- | --- | --- | --- | --- | --- | --- |
|  | **61.** | |  |  | **Awareness (if answer code 1, 2, 3, or 4 for the aspect, go directly to ask their compliance)**  **.** | | | | | | |  | |  |
|  | Please rate your agreement with the following statement on a scale from strongly disagree to strongly agree | | | | Fully | I know most of them | I know half of them | | I know some of them | I don’t know any of them | | |  |  |
|  | How aware are you of the traditional fishing rules? | | | | **1** | **2** | **3** | | **4** | **5** | | |  |  |
|  | How aware are your fellow fishermen of the traditional fishing rules? | | | | **** | **** | **** | | **** | **** | | |  |  |
|  | | How aware are you of the rules of the TURF? | | | **** | **** | **** | | **** | **** | | |  |  |
|  | | How aware are your fellow fishermen on the rules of the TURF? | | | **** | **** | **** | | **** | **** | | |  |  |
|  | COMPLIANCE | | | | Always | Most of the times | Half of the times | | Sometimes | Never | | |  |  |
|  |  |  |  |  |  |  |  |  |  |  |  |  |  |  |
|  | How often do you comply with the local fishing rules? | | | | **** | **** | **** | | **** | **** | | |  |  |
|  | How often do you think your local fishermen comply with the local fishing rules? | | | | **** | **** | **** | | **** | **** | | |  |  |
|  | How often do you comply with the rules of the TURF? | | | | **** | **** | **** | | **** | **** | | |  |  |
|  | How often do you think your local fishermen comply with the rules of the TURF? | | | | **** | **** | **** | | **** | **** | | |  |  |
|  | |  | | |  |  |  | |  |  | | |  |  |

|  | **62.** | | |  |  |  | |  | | | | | | | | | | |  |  |
| --- | --- | --- | --- | --- | --- | --- | --- | --- | --- | --- | --- | --- | --- | --- | --- | --- | --- | --- | --- | --- |
|  | Who do you talk to about the TURF fishing rules? | | | | | Have you changed your fishing behavior after talking to one of those people? | | How have you changed your fishing behavior? | | | | | | | | | | |  |  |
|  | **** | No one**=>q. 63** | | | | **** | No **=>q. 63** | **** | | | Better following the rules | | | | | | | |  |  |
|  | **** | Other fishermen | | | | **** | Yes | **** | | | Less following the rules | | | | | | | |  |  |
|  | **** | Other family members | | | |  |  | **** | | | Other, specify: ___________ | | | | | | | |  |  |
|  | **** | Skipper / owner of the ship | | | |  |  |  | | | | | | | | | | |  |  |
|  | **** | Collector | | | |  |  |  | | | | | | | | | | |  |  |
|  | **** | Other, specify:  _________ | | | |  |  |  | | | | | | | | | | |  |  |
|  |  | |  | | | | | |  |  | |  | |  | |  | |  | |  |
|  | **63.** | | Do you think the TURF concept has benefits for the community? | | | | | |  |  | | 1 | | 🞎 | | Yes | |  | |  |
|  |  | |  |  |  |  |  |  |  |  | | | 0 | 🞎 | No | |  | | | |
|  |  | |  | | | | | |  |  | |  | |  | |  | |  | |  |

|  |  | |  |  |  | |  |  |  |  | |  |  |  |  |
| --- | --- | --- | --- | --- | --- | --- | --- | --- | --- | --- | --- | --- | --- | --- | --- |
|  | **64.** | |  |  | **1.** | | If answer ‘Yes’, how often is your involvement?  **2.** | | | | | | | |  |
|  | Were/Are you involved in ….. | | | | Yes | No | The whole time | Most of the time | | | Half of the time | | Sometimes | |  |
|  | Were you involved in the setting of the TURF fishing rules? | | | | **** |  | **** | **** | | | **** | | **** | |  |
|  | Are you involved in the current management plan? | | | | **** |  |  |  | | |  | |  | |  |
|  | | Are you involved in the surveillance of the TURF? | | | **** |  | **** | **** | | | **** | | **** | |  |

|  | **65.** |  | |  |  |  |  |  |
| --- | --- | --- | --- | --- | --- | --- | --- | --- |
|  | Why are you not involved in the TURF Management? | |  | ________________________________________ | | | |  |
|  |  | |  |  |  |  |  |  |
|  |  | |  |  | 1. I am not interested | | |  |
|  |  | |  |  | 1. I don’t think TURFs make any difference | | |  |
|  |  | |  |  | 1. I don’t have time | | |  |
|  |  | |  |  | 1. Other, specify:   ____________ | | |  |
|  |  | |  |  |  |  |  |  |
|  |  | |  |  |  |  |  |  |

**5.Food Security**

*Questions should be asked to the head of household’s wife (or head of household if female)*

|  |  | |  |  | |  | |  | |  | |  |
| --- | --- | --- | --- | --- | --- | --- | --- | --- | --- | --- | --- | --- |
|  | **66.** | |  | **67.** | |  | | **68.** | |  | |  |
|  | Was the last year a good, normal or bad year for you in terms of food available | | | In the past 12 months did you ever reduce the size of your meals or skip meals because there wasn’t enough food to eat? | | | | How often did this happen – almost every month, some month but not every month or in only one or two month? | | | |  |
|  |  |  |  |  |  |  |  |  |  |  |  |  |
|  | 0 | 🞎 | Good | 0 | 🞎 | | No **->q.69** | 0 | 🞎 | | Almost every month |  |
|  | 1 | 🞎 | Normal | 1 | 🞎 | | Yes | 1 | 🞎 | | Some month but not every month |  |
|  | 2 | 🞎 | Bad |  |  | |  | 2 | 🞎 | | Only one or two month |  |
|  |  |  |  |  | | | |  | | | |  |

|  |  | | | |  |  | | |  | | |
| --- | --- | --- | --- | --- | --- | --- | --- | --- | --- | --- | --- |
| **69.** | | |  | | | | **70.** | |  | |  |
| In the past 12 months did you ever reduce the size of your children meals or let your children skip meals because there wasn’t enough food to eat? | | | | | | | How often did this happen – almost every month, some month but not every month or in only one or two month? | | | |  |
|  |  |  |  |  |  |  |  |  |  |  |  |
| 0 | | 🞎 | | No **->q. 71** | | | 0 | 🞎 | | Almost every month |  |
| 1 | | 🞎 | | Yes | | | 1 | 🞎 | | Some month but not every month |  |
|  | |  | |  | | | 2 | 🞎 | | Only one or two month |  |
|  | | | | | | |  | | | |  |

|  | **71.** | | |  | |  |  |  | |  | |  | |  | |  |  |  |
| --- | --- | --- | --- | --- | --- | --- | --- | --- | --- | --- | --- | --- | --- | --- | --- | --- | --- | --- |
|  | Were there months in the past 12 months in which you did not have enough food to meet your family’s needs? | | | | | | | | | | | | | | | | |  |
|  |  |  |  |  |  |  |  |  |  |  |  |  |  |  |  |  |  |  |
|  |  |  | | | | |  | | | | | | | | | | | |
|  | 0 | | 🞎 | | No **->q. 73** | | | |  | |  | | | | | | | |
|  | 1 | | 🞎 | | Yes | | | |  | |  | |  | |  | | | |

| **72.** |  | |  |  | |  | | | | | | | | | |  |
| --- | --- | --- | --- | --- | --- | --- | --- | --- | --- | --- | --- | --- | --- | --- | --- | --- |
|  | | Which were the months you did not have enough food to meet your family’s needs? | | | | | | | | | | | | | |  |
|  | |  |  |  |  |  |  |  |  |  |  |  |  |  |  |  |
| January | | February | | | March | | April | May | June | July | August | September | October | November | December | |
|  | |  | | |  | |  |  |  |  |  |  |  |  |  | |

|  |  |  |  |  |  | |  |  |  |  |  |  |  |  |  |  |  |
| --- | --- | --- | --- | --- | --- | --- | --- | --- | --- | --- | --- | --- | --- | --- | --- | --- | --- |
|  | **73.** | Which of these cooking devices do you have in your household?  **[ASK ONE BY ONE]** | | | | | | | | | | | | | | |  |
|  |  |  |  |  |  |  |  |  |  |  |  |  |  |  |  |  |  |
|  |  |  |  |  |  | |  |  |  |  |  |  |  |  |  |  |  |
|  |  | 1 | Open fire (3 stones) | | | | | ___ | |  | 5 | LPG stove | | | | ___ |  |
|  |  | 2 | semi-permanent firewood stove | | | | | ___ | |  | 6 | Rice Cooker | | | | ___ |  |
|  |  | 3 | Stationary firewood stove | | | | | ___ | |  | 7 | Other | | | | ___ |  |
|  |  | 4 | Kerosene stove | | | | | ___ | |  |  |  | | | |  |  |
|  |  |  |  |  |  |  | |  |  |  |  |  |  |  |  |  |  |

**6. Livestock**

|  |  |  |  |  |  |  |  |  |
| --- | --- | --- | --- | --- | --- | --- | --- | --- |
|  | **74.** | Do you have domestic animals? |  | 1 | 🞎 | Yes |  |  |
|  |  |  |  | 0 | 🞎 | No | **q.78** |  |
|  |  |  |  |  |  |  |  |  |

|  | **75.** | |  | **76.** |  |  | **77.** |  |  |  |  |
| --- | --- | --- | --- | --- | --- | --- | --- | --- | --- | --- | --- |
|  | Which animals do you currently have? | | | How many of these animals do you have? | | | Altogether, for how much did you sell animals/ products/ services within the last 12 month **[MEAT, LIVE ANIMALS, EGGS, MILK, RENT, DUNG ...]**? | | | |  |
|  |  | | |  | | | *IDR* | | | |  |
| **1.** | 🞎 | Pig | |  | | |  | | | |  |
| **2.** | 🞎 | Sheep | |  | | |  | | | |  |
| **3.** | 🞎 | Goat | |  | | |  | | | |  |
| **4.** | 🞎 | Rabbit | |  | | |  | | | |  |
| **5.** | 🞎 | Buffalo | |  | | |  | | | |  |
| **6.** | 🞎 | Horse | |  | | |  | | | |  |
| **7.** | 🞎 | Cow | |  | | |  | | | |  |
| **8.** | 🞎 | Poultry | |  | | |  | | | |  |
| **10.** | 🞎 | Other ________ | |  | | |  | | | |  |
|  |  |  | |  | | |  | | | |  |

**7. Agriculture**

|  |  |  |  |  |  |  |  |  |  |  |  |  |  |  |  |
| --- | --- | --- | --- | --- | --- | --- | --- | --- | --- | --- | --- | --- | --- | --- | --- |
|  | **78.** | |  |  |  | **79.** | |  |  |  |  |  |  |  |  |
|  | Do you cultivate farming land? | | | |  | What is the property status of your farming land? | | | | | | |  |  |  |
|  |  |  |  |  |  |  |  |  |  |  |  |  |  |  |  |
|  | 1 | 🞎 | Yes |  |  | 1 | 🞎 | your property | | | | |  |  |  |
|  | 0 | 🞎 | No | **q. 86** |  | 2 | 🞎 | rented | | | | |  |  |  |
|  |  |  |  |  |  | 3 | 🞎 | BagiHasil | | | | |  |  |  |
|  |  |  |  |  |  |  |  |  |  |  |  |  |  |  |  |

|  |  |  |  |  |  |  |  |  |  |  |  | |  |  |  |  |
| --- | --- | --- | --- | --- | --- | --- | --- | --- | --- | --- | --- | --- | --- | --- | --- | --- |
|  | **[COMMENTS]** | | | | | |  |  |  |  |  |  |  |  |  |  |
|  |  |  |  |  |  |  |  |  |  |  |  |  |  |  |  |  |
|  |  |  |  |  |  |  |  |  |  |  |  |  |  |  |  |  |
|  |  |  |  |  |  |  |  |  |  |  |  |  |  |  |  |  |

**[EXPLAIN THE DIFFERENCE BETWEEN NON-TRANSFORMED AND TRANSFORMED PRODUCTS]**

|  | **80.** |  |  | **81.** |  | **82.** |  | **83.** |  |
| --- | --- | --- | --- | --- | --- | --- | --- | --- | --- |
|  | Please indicate your five most important agricultural products? | | | Which products did you sell in a non-transformed way last year? | | How much did you sell within the last 12 month in a non-transformed way? | | For how many IDR do you sell each unit?  **[UNIT OF Q.82]** | |
| **1** | Lemon | | 🞎 | 🞎No🞎Yes | | _______ kg | |  | |
| **2** | Shallot | | 🞎 | 🞎No🞎Yes | | _______ kg | |  | |
| **3** | Chili | | 🞎 | 🞎No🞎Yes | | _______ kg | |  | |
| **4** | Cocoa | | 🞎 | 🞎No🞎Yes | | _______ kg | |  | |
| **5** | Maize | | 🞎 | 🞎No🞎Yes | | _______ kg | |  | |
| **6** | Durian | | 🞎 | 🞎No🞎Yes | | _______ kg | |  | |
| **7** | Rambutan | | 🞎 | 🞎No🞎Yes | | _______ kg | |  | |
| **8** | Beans | | 🞎 | 🞎No🞎Yes | | _______ kg | |  | |
| **9** | Peanut | | 🞎 | 🞎No🞎Yes | | _______ kg | |  | |
| **10** | Kangkung | | 🞎 | 🞎No🞎Yes | | _______ kg | |  | |
| **11** | Rubber | | 🞎 | 🞎No🞎Yes | | _______ kg | |  | |
| **12** | Potato | | 🞎 | 🞎No🞎Yes | | _______ kg | |  | |
| **13** | Cucumber | | 🞎 | 🞎No🞎Yes | | _______ kg | |  | |
| **14** | Coffee | | 🞎 | 🞎No🞎Yes | | _______ kg | |  | |
| **15** | Cabbage | | 🞎 | 🞎No🞎Yes | | _______ kg | |  | |
| **16** | Pumpkin | | 🞎 | 🞎No🞎Yes | | _______ kg | |  | |
| **17** | Mango | | 🞎 | 🞎No🞎Yes | | _______ kg | |  | |
| **18** | Mangosteen | | 🞎 | 🞎No🞎Yes | | _______ kg | |  | |
| **19** | Pineapple | | 🞎 | 🞎No🞎Yes | | _______ kg | |  | |
| **20** | Rice | | 🞎 | 🞎No🞎Yes | | _______ kg | |  | |
| **21** | Papaya | | 🞎 | 🞎No🞎Yes | | _______ kg | |  | |
| **22** | Banana | | 🞎 | 🞎No🞎Yes | | _______ bunch | |  | |
| **23** | Watermelon | | 🞎 | 🞎No🞎Yes | | _______ kg | |  | |
| **24** | Cassava | | 🞎 | 🞎No🞎Yes | | _______ kg | |  | |
| **25** | Sugar | | 🞎 | 🞎No🞎Yes | | _______ kg | |  | |
| **26** | Cloves | | 🞎 | 🞎No🞎 Yes | | _______ kg | |  | |
| **27** | Tea | | 🞎 | 🞎No🞎Yes | | _______ kg | |  | |
| **28** | Tobacco | | 🞎 | 🞎No🞎Yes | | _______ kg | |  | |
| **29** | Eggplant | | 🞎 | 🞎No🞎Yes | | _______ kg | |  | |
| **30** | Sweet Potato | | 🞎 | 🞎No🞎Yes | | _______ kg | |  | |
| **31** | Coconut | | 🞎 | 🞎No🞎 Yes | | _______ kg | | __________ | |
| **32** | Sagho | | 🞎 | 🞎No🞎 Yes | | _______ kg | | __________ | |
| **33** | __________ | |  |  | | __________ | | __________ | |

|  |  |  |  |  |  |  |  |  |  |  |  | |  |  |  |  |
| --- | --- | --- | --- | --- | --- | --- | --- | --- | --- | --- | --- | --- | --- | --- | --- | --- |
|  | **[COMMENTS]** | | | | | |  |  |  |  |  |  |  |  |  |  |
|  |  |  |  |  |  |  |  |  |  |  |  |  |  |  |  |  |
|  |  |  |  |  |  |  |  |  |  |  |  |  |  |  |  |  |
|  |  |  |  |  |  |  |  |  |  |  |  |  |  |  |  |  |

|  |  |  |  |  |  |  |  |  |
| --- | --- | --- | --- | --- | --- | --- | --- | --- |
|  | **84.** | Do you transform agricultural products? |  | 1 | 🞎 | Yes |  |  |
|  |  |  |  | 0 | 🞎 | No | 🠆**q.86** |  |
|  |  |  |  |  |  |  |  |  |

|  |  |  |  |  |  |  |  |  |
| --- | --- | --- | --- | --- | --- | --- | --- | --- |
|  | **85.** | How much do you earn per month by transforming agricultural products? |  |  |  |  |  |  |
|  |  |  |  |  |  |  | IDR |  |
|  |  |  |  |  |  |  |  |  |

1. **8. Expenditures**

**[Please try to get monthly values, if possible. Only in the cases where it is absolutely not possible to get monthly values, fill in the values per week or per year]**

|  | **86.** | |  |  | **a.** |  | **b.** |  | **c.** |  |
| --- | --- | --- | --- | --- | --- | --- | --- | --- | --- | --- |
|  | How much do you usually spend on the following items for all of the household members?**[Use -9 for natural resources expenditures]** | | | | per week | | per month | | per year | |
|  |  |  |  |  | *Rupiah* | | *Rupiah* | | *Rupiah* | |
| **1.** | 🞎 | Food | | 0 🞎 No 1 🞎Yes |  | |  | |  | |
| **2.** | 🞎 | Cigarettes | | 0 🞎No 1 🞎Yes |  | |  | |  | |
| **3.** | 🞎 | Restaurant | | 0 🞎No 1 🞎Yes |  | |  | |  | |
| **4.** | 🞎 | Mobile Phone costs | | 0 🞎No 1 🞎Yes |  | |  | |  | |
| **5.** | 🞎 | Water | | 0 🞎No 1 🞎Yes |  | |  | |  | |
| **6.** | 🞎 | Fishing Gear expenses (hooks, nets, googles etc.) | | 0 🞎No 1 🞎Yes |  | |  | |  | |
| **7.** | 🞎 | Transport (public and private) | | 0 🞎No 1 🞎Yes |  | |  | |  | |
| **8.** | 🞎 | Rent (gear, field, …) | | 0 🞎No 1 🞎Yes |  | |  | |  | |
| **9.** | 🞎 | Clothing | | 0 🞎No 1 🞎Yes |  | |  | |  | |
| **10.** | 🞎 | Medical Expenditures | | 0 🞎No 1 🞎Yes |  | |  | |  | |
| **11.** | 🞎 | Schooling Expenditures for children (schooling fees, material etc.) | | 0 🞎No 1 🞎Yes |  | |  | |  | |
| **12.** | 🞎 | Agricultural Expenditures (semence, fertilizer etc.) | | 0 🞎No 1 🞎Yes |  | |  | |  | |
| **13.** | 🞎 | Animal expenditures | | 0 🞎No 1 🞎Yes |  | |  | |  | |
| **14.** | 🞎 | Expenditures for the transformation of agricultural products | | 0 🞎No 1 🞎Yes |  | |  | |  | |
| **15.** | 🞎 | Expenditures for the transformation of fishing products | | 0 🞎No 1 🞎Yes |  | |  | |  | |
| **16.** | 🞎 | Expenditures for other household activities used to generate revenues | | 0 🞎No 1 🞎Yes |  | |  | |  | |
| **17.** | 🞎 | Ceremonies | | 0 🞎No 1 🞎Yes |  | |  | |  | |
| **18.** | 🞎 | Money sent to household member currently away | | 0 🞎No 1 🞎Yes |  | |  | |  | |
| **19.** | 🞎 | Energy expenditures (petrol, gas, electricity etc.) | | 0 🞎No 1 🞎Yes |  | |  | |  | |
| **20.** | 🞎 | Micro-credit or other kind of loan | | 0 🞎No 1 🞎Yes |  | |  | |  | |

|  |  |  |  |  |  |  |  |  |  |  |  | |  |  |  |  |  |  |
| --- | --- | --- | --- | --- | --- | --- | --- | --- | --- | --- | --- | --- | --- | --- | --- | --- | --- | --- |
|  | **[COMMENT]** | | | | | |  |  |  |  |  |  |  |  |  |  |  |  |
|  |  |  |  |  |  |  |  |  |  |  |  |  |  |  |  |  |  |  |
|  |  |  |  |  |  |  |  |  |  |  |  |  |  |  |  |  |  |  |
|  |  |  |  |  |  |  |  |  |  |  |  |  |  |  |  |  |  |  |

1. **9. Conclusion**

|  |  | | | | Improved heavily | Improved slightly | Stayed the same | Deteriorated slightly | Deteriorated a lot |
| --- | --- | --- | --- | --- | --- | --- | --- | --- | --- |
|  | **87.** | Compared to two years ago, has life | **1.** | in your household… | ① | ② | ③ | ④ | ⑤ |
|  |  |  |  |  |  |  |  |  |  |
|  |  |  | **2.** | in your village… | ① | ② | ③ | ④ | ⑤ |
|  |  |  |  |  |  |  |  |  |  |

|  |  |  |  |  | | |  | |  | |  | |  | |  |
| --- | --- | --- | --- | --- | --- | --- | --- | --- | --- | --- | --- | --- | --- | --- | --- |
|  | **88.** | How? | 1) | |  |  | |  | |  | |  | |  | |
|  |  |  |  |  | | |  | |  | |  | |  | |  |
|  |  |  | 2) |  | | |  | |  | |  | |  |  | |
|  |  |  |  |  | | |  | |  | |  | |  | |  |

|  |  |  |  |  | | |  | |  | |  |  | | |  | |  | |  |  | |  |  |
| --- | --- | --- | --- | --- | --- | --- | --- | --- | --- | --- | --- | --- | --- | --- | --- | --- | --- | --- | --- | --- | --- | --- | --- |
|  | **89.** | What is your principal source of information? | | |  | 1 | | 🞎 | | radio | | |  | 4 | | 🞎 | | Neighbours / friends | | |  | | |
|  |  |  |  |  |  | 2 | | 🞎 | | TV | | |  | 5 | | 🞎 | | Community meetings | | |  | | |
|  |  |  |  |  |  | 3 | | 🞎 | | newspaper | | |  | 6 | | 🞎 | | other ____________ | | |  | | |
|  |  |  |  |  | | |  | |  | |  |  | | |  | |  | |  |  | |  |  |

|  |  | |  |  |  |  |  | |  |  |  |  |  | |  |  |  |  |  |  |
| --- | --- | --- | --- | --- | --- | --- | --- | --- | --- | --- | --- | --- | --- | --- | --- | --- | --- | --- | --- | --- |
|  | **90.** | |  |  | **91.** | | |  |  |  |  | **92.** | | |  |  |  |  |  |  |
|  | Can you please give us your surname and name? | | |  | Can we reinterview you in two years? | | | | |  |  | Can you please give us your mobile phone number? | | | | | | | |  |
|  |  |  |  |  |  |  |  |  |  |  |  |  |  |  |  |  |  |  |  |  |
|  |  |  | |  |  |  |  |  |  |  |  |  | | | | | | | |  |
|  |  |  | |  |  | | | | |  |  |  |  |  | |  |  |  |  |  |
|  |  |  | |  | 0 | 🞎 | No | |  |  |  |  |  |  | |  | | | |  |
|  |  |  | |  | 1 | 🞎 | Yes | |  | | | | | | |  |  |  |  |  |
| Note that no one will have access to this information except for the researchers. | | | | | | | | | | | | | | | | | | | |  |
|  |  |  |  |  |  |  |  |  |  |  |  |  |  |  |  |  |  |  |  |  |
|  |  |  | |  |  |  |  | |  |  | | | | | | | | | |  |

|  |  | |  |  |  |  |  | |  | |  |  |
| --- | --- | --- | --- | --- | --- | --- | --- | --- | --- | --- | --- | --- |
|  | **93.** | |  |  |  |  |  | |  | |  |  |
|  | Could you please give us the name and telephone number of someone close to you who we can reach in case you are not available? | | | |  | **a.** | Other name and phone number given? | |  | | 0 No  1 Yes |  |
|  |  |  |  |  |  |  |  | |  | |  |  |
|  |  |  |  |  |  | **b.** | Name | | | |  |  |
|  |  |  |  |  |  |  |  | | | |  |  |
|  |  |  | |  |  | **c.** | Phone number | | |  |  |  |
|  |  |  | |  |  |  |  |  | | |  |  |
