# Supplementary material for: Catalyzing sustainable fisheries management through behavior change interventions
Source: Conserv Biol. 2020 Apr 15;34(5):1176–89. doi: 10.1111/cobi.13475 (PMC7540413; doi:10.1111/cobi.13475)
Supplement: Supplementary file 6 — Supplementary Material [file COBI-34-1176-s006.docx]

Preparation (completed by Enumerator / Committee)

No. Questionnaire

________________

Enumerator Code

________________

Name of Respondent

________________

Day / date of the interview

________________

Name of interview location

[] Selamun Village [] Kampung Baru Village [] Rajawali Village [] Merdeka Village [] Tanah Rata Village

Survey Period:

[] Pre-Campaign - Intervention [] Post Campaign - Intervention [] Pre Campaign - Comparison [] Post Campaign - Comparison

MANAGEMENT OF FISHERIES AREA (PAAP) IN KKPN TWP MARINE BANDA

Introduction

Good morning / afternoon / afternoon

We << mention the name of the TWP Laut Banda >> - intends to conduct research on the Management of access of the fishery area at Banda Sea TWP location. The purpose of this research is to know the opinion of the father / mother about it.

This survey consists of 24 statements, which I will read to you. Please be willing to Mr / Mrs to respond to this statement. This interview can be completed in approximately 40 minutes. Given the importance of this information, we hope that you are willing to answer the questions in this survey. There is no wrong and correct answer. Honesty and openness of Father / Mother is very important in providing this information. Answers Mr / Mrs will only be known by us, as researchers.

Have you ever been interviewed before?

[] Already (end the interview and say thanks) [] Not yet (continue interview)

Will you be interviewed?

[] No (end the interview and say thanks) [] Yes (continue the interview)

SELF INFORMATION

I will read some statements about you. Please kindly give us the answer that best suits you. There is only one answer for each question.

(1) Gender (filled directly by Enumerator)

[] Women [] Men

(2) What is your current age?

[] Under or equal to 17 years [] 18-24 years [] 25-31 years [] 32 - 38 years [] 39 - 45 years [] 46 - 52 years [] Above or equal to 53 years old

(3) Mention your last level of education

[] Never graduated [] Did not finish elementary school [] Graduated from elementary school / equivalent [] Junior High or equivalent [] High School graduate / equal [] Others (specify) ________________

(4) What is your main job?

[] Full-time fisherman (go to AF) [] Part-time fisherman (go to AF) [] Others (specify) ________________

The most commonly caught type of fish (one species)

________________

The type of fishing gear used

________________

Fishing time

________________

(B) The average costs incurred for fishing each time to go to sea are:

[] Under Rp 50.000 [] Rp 50.000 - Rp 100.000 [] Rp 100,000 - Rp 200,000 [] Rp 200,000 - Rp 250,000

(C) Within a month, you usually do sea activities to find as many fish

[] Less than 4 times in a month [] 5-20 times per month [] 21 - 36 times per month [] more than 36 times per month

(D) Your source of capital for fishing, usually obtained from:

[] Own / family [] Papalele / collectors [] cooperative [] borrow friend [] Others (specify) ________________

(E) In 1 month, how did your catch compare to the same month last year?

[] Same [] More [] More and more bigger [] Less [] Uncertain [] Smaller [] Do not remember / do not know

(F) In this 1 month, to get the same amount of fish as last year, your fishing distance:

[] Same course [] Closer than last year [] Farther than last year [] Not sure [] Do not remember

(5) What is the number of your family members in one house (including yourself)

[] 1 person (just yourself) [] 2 people [] 3 people [] 4 people [] equal or more than 5 people

(6) Average monthly expenditure of your family is:

[] Under Rp 500,000 [] Rp 500,000 - Rp 1,000,000 [] Rp 1.000.000 - Rp 2,000,000 [] above Rp 2,000,000

FISHERY MANAGEMENT

Here are two questions about fisheries management. Please feel free to give the best answer according to your opinion.

(7) In your own words, please explain what is meant by Area Fisheries Access Management. (If the respondent answers "Not Know", write "Do not Know")

________________

(8) State all existing rules for management of fishery area access

________________

DAILY HABITS IN SEARCHING AND MANAGING SEA MARKETS

Here are some statements about the habits of finding and managing seafood. Please kindly give your answer in accordance with the habits and beliefs of Mr / Ms.

(9) Other people in this village, who set an example for me to find fish according to the rules are:

[] Head of village [] Chairman of RT [] Fellow fisherman [] Imam Mosque [] Wife / husband [] Children [] UPTD Fisheries [] None [] Others (specify) ________________

(10) Other people in this village who require me to find fish according to the rules is

[] Head of village [] Chairman of RT [] Fellow fisherman [] Imam Mosque [] Wife / husband [] Children [] UPTD Fisheries [] None [] Others (specify) ________________

For the statement below, please state your answer, with "Yes", "No", or 'Can not remember'

(11) In the last 6 months, I talked with fellow fishermen about:

(A) benefits gained from the management of the fishery area access

[] Yes [] No [] Do not remember [] forgot

(B) compliance with applicable rules within the territory of the fishery access area

[] Yes [] No [] Do not remember [] forgot

(C) ways of monitoring and reporting violations of rules in the area of ​​access of the fishing area

[] Yes [] No [] Do not remember [] forgot

Here, please tell me whether 'easy,' rather easy ',' hesitant ',' rather difficult ', difficult' to do things yourself in this statement.

(12) For Mr / Ms

(A) does not look for fish in the no take / red zone

[] Easy [] Somewhat easy [] Hesitant [] Somewhat difficult [] Difficult

(B) complies with the rules of access management of the fishing area

[] Easy [] Somewhat easy [] Hesitant [] Somewhat difficult [] Difficult

(C) engage in processes and discussions for the management rules of the fishery area access

[] Easy [] Somewhat easy [] Hesitant [] Somewhat difficult [] Difficult

(D) report the catch

[] Easy [] Somewhat easy [] Hesitant [] Somewhat difficult [] Difficult

(E) report a violation of the rules in the area of ​​access of the fishery area

[] Easy [] Somewhat easy [] Hesitant [] Somewhat difficult [] Difficult

(F) invites fellow fishermen to comply with the management rules of fisheries area access

[] Easy [] Somewhat easy [] Hesitant [] Somewhat difficult [] Difficult

Here, please Mr / Mrs declare whether 'Agreed', 'Disagree', 'Do not know' to the statement below

(13) For you to comply with the rules of management of fishery area access

(A) is a form of responsibility as a fisherman in the region

[] Agree [] Disagree [] Do not know

(B) ensuring the continuation of my family's life in the future

[] Agree [] Disagree [] Do not know

(C) maintaining the availability of fish and other marine resources for a long time

[] Agree [] Disagree [] Do not know

(D) preserves the traditions of life as fishermen from generation to generation

[] Agree [] Disagree [] Do not know

(14) For Mr / Ms, adhere to the rules of management of fishery area access

(A) will incur additional costs to replace fishing gear

[] Agree [] Disagree [] Do not know

(B) can not be done because there is no firmness against rule violators

[] Agree [] Disagree [] Do not know

(C) can not be executed because there is no visible border for the location of the fishery area access

[] Agree [] Disagree [] Do not know

(D) makes the time to go to sea longer due to reporting the catch

[] Agree [] Disagree [] Do not know

Here's what you want to do, 'Somewhat sure to be able to do', 'Doubtful', 'Somewhat unsure of being able to do', 'Unsure able to do' the following statements.

(15) Mr / Mrs feel,

(A) does not catch fish in the no-take area

[] Sure able to do [] Somewhat sure able to do [] Hesitant [] Somewhat unsure able to do [] Not sure able to do

(B) catch fish according to the rules in the area of ​​fishery access area

[] Sure able to do [] Somewhat sure able to do [] Hesitant [] Somewhat unsure able to do [] Not sure able to do

(C) using the type of fishing gear permitted in the area of ​​fishery access area

[] Sure able to do [] Somewhat sure able to do [] Hesitant [] Somewhat unsure able to do [] Not sure able to do

(D) reporting the catch

[] Sure able to do [] Somewhat sure able to do [] Hesitant [] Somewhat unsure able to do [] Not sure able to do

(E) supervise and report violations in the area of ​​access of the fishing area

[] Sure able to do [] Somewhat sure able to do [] Hesitant [] Somewhat unsure able to do [] Not sure able to do

(16) (Enumerator provides maps and explains how to read maps to respondents Enumerators then fill in answers according to the accuracy / inaccuracy of respondents).

The enumerator read this question to the respondent:

From this map, point to / mention all the locations you usually go looking for fish

(Enumerator: Writing all respondent's answer If not willing to answer write 'No answer')

________________

(A) Based on the location of the above mentioned fishing / fowl, please Mr / Mrs choose the statement that best describes you right now

[] I do not know the designation rules for this area and do not think to find out [] I do not know the designation rules for this area but have been thinking about finding out [] I am not implementing the designation rules for this area but in the near future I think to do it [] I have followed the designation rules for this area, but only implemented it for less than 6 months [] I have followed the rules of the designation of this area and have done so in 6 months or more

For the following statement, please choose the one that best describes you right now

(17) For the following statement, please choose the one that best describes you right now

[] I do not know the rules about fishing gear allowed in the area of ​​access area and do not think to find out [] I do not know the rules of fishing gear are allowed in the area of ​​access area but in the near future thought to find out [] I already know fishing gear Which is allowed in the area of ​​access area and in the near future it is thought to do it [] I have been using the type of fishing gear according to the rules in the area of ​​access area, for less than 6 months [] I have used the type of fishing gear that match the rules of area access area, 6 months or more

(18) For the following statement, please choose the one that best describes you right now

[] I do not know the rules about the size of the catch in the area of ​​access area and do not think to find out [] I do not know the catch size rules in the area of ​​access area but in the near future it is thought to find out [] I already know the size of the catch allowed in the region Access area and in the near future think to do it [] I have caught fish with the size of fish catch according to the rules in the area of ​​access area, and have been doing it for less than 6 months [] I have captured the size of the fish according to the rules of area access area and have done it in 6 Months or more

(19) For the following statement, please choose the one that best describes you right now

[] I did not participate in the management of area access areas and did not think to do it [] I did not participate in the management of area access areas but have thought to find out [] I have thought about participating in the management of area access areas in the near future [] I have Participate in the management of area access areas, but only implement them for less than 6 months [] I have participated in the management of area access areas and have done so in 6 months or more

(20) For the following statement, please choose the one that best describes you right now

[] I have never been involved in surveillance of the territory of the fisheries access area and have not thought of doing it [] I have never been involved in surveillance of area access areas and thought to find out [] I was thinking of being involved in the monitoring of area access areas, in the near future [] I Has been involved in the supervision of area access areas, less than 6 months [] I have been involved in the supervision of the area of ​​access to the fishing area, within 6 months or more

MEDIA EFFECTIVENESS

(21) What activities do you think most effectively convey information about PAAP here:

[] Launching PAAP Program [] Safari Ramadhan [] Street Quiz [] Fisherman's Meeting [] Capture Training [] Banda Lesatari Formation [] Environmental Education for Elementary School, Junior High School, Senior High [] Welcoming Competition HUT RI August 17 [] Nobar Film [] Fishing Contest 5 Country [] Nothing Effective [] Do not Know [] Others (specify) ________________

(22) What activities do you think are most effective in conveying information about fisheries management rules here:

[] Launching PAAP Program [] Safari Ramadhan [] Street Quiz [] Fisherman's Meeting [] Capture Training [] Banda Lesatari Formation [] Environmental Education for Elementary School, Junior High School, Senior High [] Welcoming Competition HUT RI August 17 [] Nobar Film [] Fishing Contest 5 Country [] Nothing Effective [] Do not Know [] Others (specify) ________________

(23) What media do you think is the most effective way of passing information about PAAP here:

[] Banner Shop / Warun g [] Banner Eid [] Banner Ramadan [] Schedule Imsyakiah [] Video Banda Lestari [] hat [] Sticker PAAP [] PIN / Bros. [] Kaos Event [] Photos Novela [] Poster [] Calendar 2017 [] Pen [] Umbul-Umbul [] Mascot [] Do not know [] Nothing effective [] Others (specify) ________________

(24) What media are you most effective in conveying information about fisheries management rules here:

[] Banner Banner [] Idul Fitri Banner [] Banner Ramadhan [] Imsyakiah Schedule [] Banda Lestari [] Hat [] Sticker PAAP [] PIN / Brooch [] Activity Shirt [] Novela Photo [] Poster [] Calendar 2017 [] Pen [] Umbul-Umbul [] Mascot [] Do not know [] Nothing is effective [] Others (specify) ________________

*****

Thank you for your willingness to take the time to answer this survey.
