# Supplementary material for: Catalyzing sustainable fisheries management through behavior change interventions
Source: Conserv Biol. 2020 Apr 15;34(5):1176–89. doi: 10.1111/cobi.13475 (PMC7540413; doi:10.1111/cobi.13475)
Supplement: Supplementary file 7 — Supplementary Material [file COBI-34-1176-s007.docx]

Preparation (completed by Enumerator)

No. Questionnaire

________________

Name of Enumerator

________________

Name of Respondent

________________

Census Execution Date

________________

Village Name

[] Desa Mertak [] Sengkol Village

Survey Period:

[] Pre-Campaign - Intervention [] Post Campaign - Intervention [] Pre Campaign - Comparison [] Post Campaign - Comparison

OPINIONAL POLICY ON THE MANAGEMENT OF FISHERY AREA ACCESS TO BUKBANG TELUKAN

Introduction

Good morning / afternoon / afternoon

I << name myself >> I am currently assisting the Central Lombok and Fishery Service of Central Lombok to conduct research on the Management of Fishery Area Access at Bumbang Bay. The purpose of this research is to know what you think about it.

This survey consists of 19 statements, which I will read to you. Please kindly respond to this statement. This interview can be completed in approximately 40 minutes. Given the importance of this information, we hope that you are willing to answer the questions in this survey. There is no wrong and correct answer. Honesty and openness are very important in providing this information.Your answer will only be known to us, as a researcher.

Have you ever been interviewed before

[] It has been [] Not yet

Are you willing to be interviewed?

[] No [] Yes

SELF INFORMATION

I will read some statements about you. Please kindly give us the answer that best suits you. There is only one answer for each question.

(1) What is the age of the father at this time?

[] Under or equal to 17 years [] 18-24 years [] 25-31 years [] 32-38 years [] 39-45 years [] 46-52 years [] Above or equal to 53 years old

(2) Have you ever been to school where?

[] Never graduated [] Did not finish elementary school [] Graduated from elementary school / equivalent [] Junior High or equivalent [] High School graduate / equal [] Others (specify) ________________

(3) What is your main job?

[] Full-time fisherman [] Fishermen part-time

(A) What type of lobster do you catch most often?

[] Sand lobster [] Pearl lobster [] Sand lobster and pearl [] Others (specify) ________________

(B) What fishing equipment is used?

[] Nets [] Compressors [] Others (specify) ________________

(C) What type of vessel is used?

[] Boat katinting [] Boat katir [] Boat machine in [] Others (specify) ________________

(D) When did it catch him?

[] 5 pm to 10 am [] 7 pm to 11 pm

(E) The source of father's capital to go to sea is obtained from where?

[] Own / family [] Cooperative [] Collector [] Friends / fellow fisherman [] Others (specify) ________________

(F) How many family members do you have in one house? (Including your own)

[] 2 people [] 3 people [] 4 people [] 5 people [] more than 5 people

(G) What is the average monthly family expenditure?

[] Below 1 million [] Between 1 million - 2 million [] Above 2 million

(H) What is the average cost that you spend looking for the lobster whenever you go down to sea?

[] Under 250 thousand [] 250 thousand - 500 thousand [] Above 500 thousand

(I) How many times do you go down to sea in a month to find lobsters?

[] Less than 8 times in a month [] 8 - 16 times a month [] 16 - 18 times [] 18 - 24 times [] 24 - 30 times [] more than 30 times

(4) How did you catch this month compared to the same month last year?

[] More [] more [] more and more [] fewer [] indefinite

(5) How is your father's fishing distance in one month, to get the same amount of lobster as last year?

[] Just the same [] closer than last year [] farther than last year [] do not remember [] indeterminate

FISHERY MANAGEMENT

Here are two questions about fisheries management. Please kindly give the best answer according to your opinion.

(6) Sir, please explain what is meant by Area Fisheries Access Management with your own words.

________________

(7) Please explain all existing rules for Area Fisheries Access Management

________________

DAILY HABITS IN SEARCHING AND MANAGING SEA MARKETS

Here are some statements about the habits of finding and managing seafood. Please kindly give your answer in accordance with the habits and beliefs of Mr / Ms.

(8) Who is the example for you to search for lobster parent according to the rules in this village?

[] Head of the hamlet [] Chairman of the fishing group [] Fellow fisherman [] Wife [] Staff of the Central Lombok and Fishery Service of Lombok [] None [] Do not know [] Others (specify) ________________

(9) Who requires you to search for lobster according to the rules in this village?

[] Head of the hamlet [] Chairman of the fishing group [] Fellow fisherman [] Wife [] Staff of the Central Lombok and Fishery Service of Lombok [] None [] Do not know [] Others (specify) ________________

For the statement below, please state your answer, with "Yes", "No", or 'Can not remember'

(10) In the last 6 months, I talked to other fellow fishermen about:

(A) benefits gained from the management of the fishery area access

[] Yes [] No [] Do not remember

(B) compliance with applicable rules within the territory of the fishery access area

[] Yes [] No [] Do not remember

(C) ways of monitoring and reporting violations of rules in the area of ​​access of the fishing area

[] Yes [] No [] Do not remember

Here, please tell me whether 'easy,' rather easy ',' hesitant ',' rather difficult ', difficult' to do things yourself in this statement.

(11) For me,

(A) not looking for fish in the core zone

[] Easy [] Somewhat easy [] Hesitant [] Somewhat difficult [] Difficult

(B) complies with the rules of access management of the fishing area

[] Easy [] Somewhat easy [] Hesitant [] Somewhat difficult [] Difficult

(C) engage in processes and discussions for the management rules of the fishery area access

[] Easy [] Somewhat easy [] Hesitant [] Somewhat difficult [] Difficult

(D) report the catch

[] Easy [] Somewhat easy [] Hesitant [] Somewhat difficult [] Difficult

(E) report a violation of the rules in the area of ​​access of the fishery area

[] Easy [] Somewhat easy [] Hesitant [] Somewhat difficult [] Difficult

(F) invites fellow fishermen to comply with the management rules of fisheries area access

[] Easy [] Somewhat easy [] Hesitant [] Somewhat difficult [] Difficult

Here, please tell me whether 'Agreed', 'Disagree', 'Do not know' to the statement below

(12) To me, by obeying the rules of access management of the fishing area

(A) Then the catch in the future will add more when compared to before the establishment of PAAP

[] Agree [] Disagree [] Do not know

(B) The location of the fishing will be closer

[] Agree [] Disagree [] Do not know

(C) The abundance of lobsters will increase from the core zone

[] Agree [] Disagree [] Do not know

(D) The size of the lobster catch will be uniform

[] Agree [] Disagree [] Do not know

(E) Will make the time go to sea reduced so that time with family more and more

[] Agree [] Disagree [] Do not know

(13) To me, by obeying the rules of access management of the fishing area

(A) Causes reduced fishing location

[] Agree [] Disagree [] Do not know

(B) Lobster catch will be reduced

[] Agree [] Disagree [] Do not know

(C) Causes trouble when catching lobsters because they can not dive without tools

[] Agree [] Disagree [] Do not know

(D) Increase employment due to having to report the catch

[] Agree [] Disagree [] Do not know

Here, please tell me whether 'Sure able to do', 'Somewhat sure to do', 'Doubtful', 'Somewhat unsure of being able to do', 'Unsure able to do' the statements below.

(14) I feel,

(A) does not catch fish in the no-take area

[] Sure able to do [] Somewhat sure able to do [] Hesitant [] Somewhat unsure able to do [] Not sure able to do

(B) arresting losbter according to the rules in the area of ​​access of the fishing area

[] Sure able to do [] Somewhat sure able to do [] Hesitant [] Somewhat unsure able to do [] Not sure able to do

(C) using the type of fishing gear permitted in the area of ​​fishery access area

[] Sure able to do [] Somewhat sure able to do [] Hesitant [] Somewhat unsure able to do [] Not sure able to do

(D) reporting the catch

[] Sure able to do [] Somewhat sure able to do [] Hesitant [] Somewhat unsure able to do [] Not sure able to do

(E) supervise and report violations in the area of ​​access of the fishing area

[] Sure able to do [] Somewhat sure able to do [] Hesitant [] Somewhat unsure able to do [] Not sure able to do

(15) (Enumerator provides maps and explains how to read maps to respondents Enumerators then fill in answers according to the accuracy / inaccuracy of respondents).

The enumerator read this question to the respondent:

From this map, please show / name all the locations where you usually go to look for lobsters

(Enumerator: Writing all respondent's answer If not willing to answer write 'No answer')

________________

(A) Based on the location of the fishing lobster you mentioned earlier, please select the statement that best describes yourself the current father

[] I do not know the designation rules for this area and do not think to find out [] I do not know the designation rules for this area but have been thinking about finding out [] I am not implementing the designation rules for this area but in the near future I think to do it [] I have followed the designation rules for this area, but only implemented it for less than 6 months [] I have followed the rules of the designation of this area and have done so in 6 months or more

For the following statement, please select the one that best describes your current father / mother

(16) For the following statement, please select the one that best describes your current father / mother

[] I do not know the rules about fishing gear allowed in the area of ​​access area and do not think to find out [] I do not know the rules of fishing gear are allowed in the area of ​​access area but in the near future thought to find out [] I already know fishing gear Which is allowed in the area of ​​access area and in the near future it is thought to do it [] I have been using the type of fishing gear according to the rules in the area of ​​access area, for less than 6 months [] I have used the type of fishing gear that match the rules of area access area, 6 months or more

(17) For the following statement, please select the one that best describes you now

[] I do not know the rules about the size of the catch in the area of ​​access area and do not think to find out [] I do not know the catch size rules in the area of ​​access area but in the near future it is thought to find out [] I already know the size of the catch allowed in the region Access area and in the near future think to do it [] I have caught fish with the size of fish catch according to the rules in the area of ​​access area, and have been doing it for less than 6 months [] I have captured the size of the fish according to the rules of area access area and have done it in 6 Months or more

(18) For the following statement, please select the one that best describes you now

[] I did not participate in the management of area access areas and did not think to do it [] I did not participate in the management of area access areas but have thought to find out [] I have thought about participating in the management of area access areas in the near future [] I have Participate in the management of area access areas, but only implement them for less than 6 months [] I have participated in the management of area access areas and have done so in 6 months or more

(19) For the following statement, please select the one that best describes you now

[] I have never been involved in surveillance of the territory of the fisheries access area and have not thought of doing it [] I have never been involved in surveillance of area access areas and thought to find out [] I was thinking of being involved in the monitoring of area access areas, in the near future [] I Has been involved in the supervision of area access areas, less than 6 months [] I have been involved in the supervision of the area of ​​access to the fishing area, within 6 months or more

MEDIA EFFECTIVENESS

(20) What activities do you think most effectively convey information about PAAP here:

[] Art festivals [] Fishing meetings [] Religious activities [] Nothing effective [] Others (specify) ________________

(21) What activities do you think are most effective in conveying information about fisheries management rules here:

[] Art festivals [] Fishing meetings [] Religious activities [] Nothing effective [] Others (specify) ________________

(22) What kind of media you feel the most effective to convey information about PAAP here is:

[] Stall banner [] Poster [] Calendar [] Nothing effective [] Others (specify) ________________

(23) What media do you think is the most effective way of conveying information about fishery rules here:

[] Stall banner [] Poster [] Calendar [] Nothing effective [] Others (specify) ________________

*****

Thank you for taking the time to answer this survey.
