# Supplementary material for: Catalyzing sustainable fisheries management through behavior change interventions
Source: Conserv Biol. 2020 Apr 15;34(5):1176–89. doi: 10.1111/cobi.13475 (PMC7540413; doi:10.1111/cobi.13475)
Supplement: Supplementary file 8 — Supplementary Material [file COBI-34-1176-s008.docx]

Preparation (completed by Enumerator / Committee)

No. Questionnaire

________________

Enumerator Code

________________

Day / date of the interview

________________

Name of interview location

[] Village Poopoh [] Village Teling [] Village Kumu [] Pinasungkulan Village [] Rap-Rap Village [] Arakan Village [] Sondaken Village [] Pungkol Village [] Wawontulap Village [] Desa Popareng

Survey Period:

[] Pre-Campaign - Intervention [] Post Campaign - Intervention [] Pre Campaign - Comparison [] Post Campaign - Comparison

FISHERMAN'S OPPORTUNITIES OF FISHERMAN'S DISTRIBUTORS - WAWONTULAP ABOUT THE MANAGEMENT OF FISHERIES AREA (PAAP) ACCESS IN THE NATIONAL PARK BUNAKEN

Introduction

Good morning / afternoon / afternoon

We from Bunaken National Park intend to hold a poll on the management of fishery area access (PAAP) in the Arakan-Wawontulap coastal area of ​​Bunaken National Park, with the aim of collecting fisheries management data by the buffer fishermen in the Arakan-Wawontulap coastal Bunaken National Park.

This survey consists of 20 statements, which I will read to you. Please be willing to Mr / Mrs to respond to this statement. This interview can be completed in approximately 40 minutes. Given the importance of this information, we hope that you are willing to answer the questions in this survey. There is no wrong and correct answer. Honesty and openness of Father / Mother is very important in providing this information. Answers Mr / Mrs will only be known by us, as researchers.

Have you ever been interviewed before?

[] Already (end the interview and say thanks) [] Not yet (continue interview)

Will you be interviewed?

[] No (end the interview and say thanks) [] Yes (continue the interview)

SELF INFORMATION

I will read some statements about you. Please kindly give us the answer that best suits you. There is only one answer for each question.

(1) Gender (filled directly by Enumerator)

[] Women [] Men

(2) What is your current age?

[] <17 years (end of interview and thank you) [] 17 - 20 years [] 21 - 25 years [] 26 - 30 years [] 31 - 35 years [] 36 - 40 years [] 41 - 45 years [ ] 46 - 50 years [] 51 - 55 years []> 55 years

(3) Final education Mr / Ms

[] Never graduated from elementary school [] Graduated from elementary school [] Graduated from elementary / high school [] Graduated from Junior High School / MTs [] Not Graduated Junior High School / MTs High School / SMEA / STM / Madrasah Aliyah [] Not Graduated High School / SMEA / STM / Madrasah Aliyah [] Graduate D3 [] Bachelor's Degree [] Others (specify) ________________

(4) What is the main livelihood of Mr / Ms?

[] Full-time fisherman [] Part-time fisherman [] Seasonal fisherman [] Others (specify) ________________

The most frequent thing do you do the following?

(A) Which types of fish are most often caught?

[] Behang [] Goropa [] Bob [] Octopus [] Suntung [] Others (specify) ________________

Other types of fish catch

________________

(B) What kind of fishing gear is used?

[] Soma [] Fishing [] Sero [] Jubi [] Dadofa [] Others (specify) ________________

(C) Fishing time?

[] At 4 am - 8 am [] 6 am - 3 pm [] 4 pm - 8 pm [] 4 pm - 6 am [] Others (specify) ________________

(D) The average costs incurred for fishing each time to go to sea are:

[] Under Rp 50.000 [] Rp 50.000 - Rp 100.000 [] Rp 125.000 - Rp 175.000 [] Above Rp 200,000

(E) Within a span of one week, how many times do you go to the sea to catch fish

[] 7 times / day [] 14 times / day twice [] 6 times (every day except Friday) [] 3 times [] Others (specify) ________________

(F) Source of capital Mr / Ms to go to sea, usually obtained from:

[] Self-possession (wife's gift) [] collector [] borrow to relatives [] borrow neighbors [] borrow group leader [] Others (specify) ________________

(G) According to Mr / Mrs in this month, how the catch compared to the same month last year?

[] Same [] More [] More and more bigger [] Less [] Uncertain [] Do not remember / do not know

(H) According to Mr / Ms in a month, to get the same number of fish with last year, what is the distance of sea:

[] Same course [] Closer than last year [] Farther than last year [] Not sure [] Do not remember

(5) What is the number of family members in a house that is dependent Mr / Mrs (including yourself)

[] 1 person [] 2 people [] 3 people [] 4 people [] more than 5 people

(6) The average family expenditure of Mr / Ms each month is:

[] Less than Rp. 1 million [] Rp. 1 million - Rp. 1.5 million [] Rp. 1.6 million - Rp. 2 million [] above Rp. 2 million

FISHERY MANAGEMENT

Here are two questions about fisheries management. Please feel free to give the best answer according to your opinion.

(7) In your own words, please explain what is meant by Area Fisheries Access Management. (If the respondent answers "Not Know", write "Do not Know")

________________

(8) What are the agreements established by the fishing communities for the management of fishery area access

________________

DAILY HABITS IN SEARCHING AND MANAGING SEA MARKETS

Here are some statements about the habits of finding and managing seafood. Please kindly give your answer in accordance with the habits and beliefs of Mr / Ms.

(9) Other people in this village, who set an example for Mr / Ms to fish in accordance with the rules are:

[] DKP (Prov / Kab) [] Bunaken National Park [] ANTRA [] Chairman of Fishermen Group [] Fisheries Extension [] Collector [] Fellow Fisherman [] Imam Masjid [] Pinatua [] Lecturer (his name). ........... [] Wife / husband [] Child [] None [] Do not know [] Other (specify) ________________

(10) Others in this village who require me to fish in accordance with the agreement is

[] DKP (Prov / Kab) [] Bunaken National Park [] ANTRA [] Chairman of Fishermen Group [] Fisheries Extension [] Collector [] Fellow Fisherman [] Imam Masjid [] Pinatua [] Lecturer (his name). ........... [] Wife / husband [] Child [] None [] Do not know [] Other (specify) ________________

(11) In the last 6 months, have you ever met with fellow fishermen to form a group of fishermen?

[] 1 month [] once a week [] gathered at any time [] new intent group [] never

(12) Which of the following statements is most suitable for you?

[] I have never participated to verify myself as a fisherman [] I have not intend to participate to verify myself as a fisherman [] I intend to participate verify myself as a fisherman [] I have participated to verify myself as a fisherman

For the statement below, please state your answer, with "Yes", "No", or 'Can not remember'

(13) In the last 6 months, Mr / Ms spoke with other fishermen about:

(A) benefits derived from the Management of Fisheries Area Access (PAAP)

[] Yes [] No [] Do not remember

(B) compliance with applicable agreements within the territory of the fishery access area

[] Yes [] No [] Do not remember

(C) supervision and reporting of breach of agreement in the area of ​​access of the fishery area

[] Yes [] No [] Do not remember

(D) fishing gear is allowed in the area of ​​access of the fishing area

[] Yes [] No [] Do not remember

(E) the size of the fish caught in the area of ​​access of the fishery area

[] Yes [] No [] Do not remember

(F) types of fish that are managed and regulated in the area of ​​fishery area access

[] Yes [] No [] Do not remember

Here, please tell me whether 'easy,' rather easy ',' hesitant ',' rather difficult ', difficult' to do things yourself in this statement.

(14) For me,

(A) does not catch fish in the fish saving area

[] Easy [] Somewhat easy [] Hesitant [] Somewhat difficult [] Difficult

(B) comply and obey the agreement of management rules of access of fisheries area

[] Easy [] Somewhat easy [] Hesitant [] Somewhat difficult [] Difficult

(C) are involved in the process and discussion for agreement on management rules of fishery area access

[] Easy [] Somewhat easy [] Hesitant [] Somewhat difficult [] Difficult

(D) reported fish catch results

[] Easy [] Somewhat easy [] Hesitant [] Somewhat difficult [] Difficult

(E) report violations of the rules agreement in the area of ​​access of the fishery area

[] Easy [] Somewhat easy [] Hesitant [] Somewhat difficult [] Difficult

(F) invite fellow fishermen to comply with the agreement of management of fishery area access

[] Easy [] Somewhat easy [] Hesitant [] Somewhat difficult [] Difficult

Here, please Mr / Mrs declare whether 'Agreed', 'Disagree', 'Do not know' to the statement below

(15) For me, obey the agreement of management rules of access of fisheries area

(A) is a form of responsibility as a fisherman in the region

[] Agree [] Disagree [] Do not know

(B) assure my family's future work and survival

[] Agree [] Disagree [] Do not know

(C) keep the fish there for a long time

[] Agree [] Disagree [] Do not know

(D) preserves the traditions of life as fishermen from generation to generation

[] Agree [] Disagree [] Do not know

(E) will benefit fishermen based on their activities

[] Agree [] Disagree [] Do not know

(F) is a need that must be met by fishermen

[] Agree [] Disagree [] Do not know

(16) For me, obey the agreement of management rules of access of fishery area

(A) will incur additional costs to replace fishing gear

[] Agree [] Disagree [] Do not know

(B) can not be done because there is no firmness against rule violators

[] Agree [] Disagree [] Do not know

(C) can not be executed because there is no visible border for the location of the fishery area access

[] Agree [] Disagree [] Do not know

(D) makes the time to go to sea longer due to reporting the catch

[] Agree [] Disagree [] Do not know

(E) there is no benefit in the presence of fish saving areas

[] Agree [] Disagree [] Do not know

(F) the emergence of social conflicts between fishermen

[] Agree [] Disagree [] Do not know

Here's what you want to do, 'Somewhat sure to be able to do', 'Doubtful', 'Somewhat unsure of being able to do', 'Unsure able to do' the following statements.

(17) I feel,

(A) does not catch fish in the fish saving area

[] Sure able to do [] Somewhat sure able to do [] Hesitant [] Somewhat unsure able to do [] Not sure able to do

(B) catching fish according to the agreement at the PAAP location

[] Sure able to do [] Somewhat sure able to do [] Hesitant [] Somewhat unsure able to do [] Not sure able to do

(C) using an agreed fishing gear at the PAAP site

[] Sure able to do [] Somewhat sure able to do [] Hesitant [] Somewhat unsure able to do [] Not sure able to do

(D) reported fish catch results

[] Sure able to do [] Somewhat sure able to do [] Hesitant [] Somewhat unsure able to do [] Not sure able to do

(E) supervise and report the breach of agreement at the PAAP location

[] Sure able to do [] Somewhat sure able to do [] Hesitant [] Somewhat unsure able to do [] Not sure able to do

(18) (Enumerator provides maps and explanations on how to read the map to respondents Enumerators then fill in answers according to the accuracy / inaccuracy of respondents).

The enumerator read this question to the respondent:

From this map, can you point to all locations where you usually go to fish

(Enumerator: Writing all respondent's answer If not willing to answer write 'No answer')

________________

(A) Based on the location of the fishing that has been mentioned, please choose the statement that best describes yourself Mr / Ms at this time

[] I do not know the designation rules for this location and do not think to find out [] I do not know the designation rules for this location but have thought to find out [] I did not enforce the designation rules for this location but in the near future it was thought to do so [] I have followed the designation rules for this location, but only implemented it for less than 6 months [] I have followed this location designation rule and have done so in 6 months or more

For the following statement, please choose the one that best describes you right now

(19) For the following statement, please choose the one that best describes you right now

[] I do not know the agreement about the permit rules on the PAAP site and do not think to find out [] I do not know the agreement of the rules of fishing gear allowed in the PAAP location but in the near future it is thought to find out [] I already know the fishing gear Which is allowed in the PAAP location and in the near future it is thought to do it [] I have been using the type of fishing gear according to the agreement of the rules in PAAP location, for less than 6 months [] I have used the type of fishing gear which is in agreement with PAAP location rules, in 6 Months or more

(20) For the following statement, please select the most appropriate description of yourself / Mrs at this time

[] I do not know the agreement about the size of the fish in the PAAP location and do not think to find out [] I do not know the agreement of the fishing catch size rule in PAAP location but in the near future I think to find out [] I know the size of the fish catch Is allowed at the PAAP site and in the near future it is thought to do it [] I have caught fish with the size of the fish catch according to the arrangement of the rules at the PAAP location, and have done it less than 6 months [] I have captured the size of the fish according to the agreement of the rules at the PAAP location and Do it in 6 months or so

(21) For the following statement, please choose the one that best describes you right now

[] I did not participate in PAAP and did not think to do it [] I did not participate in PAAP but had thought to find out [] I have thought to participate in PAAP in the near future [] I have participated in PAAP, but just implemented it less than 6 months [] I have participated in PAAP and have done it in 6 months or more

(22) For the following statement, please choose the one that best describes you right now

[] I have never been involved in PAAP location surveillance and have not thought of doing it [] I have never been involved in PAAP location surveillance and thought to find out [] I was thinking about getting involved in PAAP location surveillance in the near future [] I was already involved in surveillance PAAP location, less than 6 months [] I have been involved in location surveillance, within 6 months or more

(23) What activities do you think most effectively convey information about PAAP here:

[Event Festival] [] Events / Festivals [] Religious Events [] Festival Tatapaan [] Mass Treatment [] Festival of Conservation of North Sulawesi [] Events / Events August 17 [] Development Parade [] Even / ] Santaklause [] Fisherman's Workshop [] Coastal and village net activities [] Lesson activities in Sunday School [] Traditional races [] Daseng [] Activities Socialization of forest protection, conservation and animals [] Safari / official visits [] None Effective [] Others (specify) ________________

(24) What activities do you think most effectively convey information on fisheries management rules here:

[Event Festival] [] Events / Festivals [] Religious Events [] Festival Tatapaan [] Mass Treatment [] Festival of Conservation of North Sulawesi [] Events / Events August 17 [] Development Parade [] Even / ] Santaklause [] Fisherman's Workshop [] Coastal and village net activities [] Lesson activities in Sunday School [] Traditional races [] Daseng [] Activities Socialization of forest protection, conservation and animals [] Safari / official visits [] None Effective [] Others (specify) ________________

(25) What kind of media you feel most effective in conveying information about PAAP here:

[] Banner banner [] Poster [] Calendar [] Billboards / banners [] Banners [] Hats [] Kaos [] Stickers [] Wall clock [] Muk [] School bag [] Balpoin [] Trophy / Cup [] Track PAAP [] Short film PAAP [] Flipchart [] Flyer [] Prayer sheet [] Social Media Facebook [] Blog [] Media Print Newspaper (Tribune Manado) [] Media on line www.villagerspost.com [] Media on line www .cendananews.com [] Media on line www.tribunnews.manado.com [] Media radio (Smart Fm 101.2 Manado) [] Nothing effective [] Others (specify) ________________

(26) What activities do you think most effectively convey information about fisheries management rules here:

[] Banner banner [] Poster [] Calendar [] Billboards / banners [] Banners [] Hats [] Kaos [] Stickers [] Wall clock [] Muk [] School bag [] Balpoin [] Trophy / Cup [] Track PAAP [] Short film PAAP [] Flipchart [] Flyer [] Prayer sheet [] Social Media Facebook [] Blog [] Media Print Newspaper (Tribune Manado) [] Media on line www.villagerspost.com [] Media on line www .cendananews.com [] Media on line www.tribunnews.manado.com [] Media radio (Smart Fm 101.2 Manado) [] Nothing effective [] Others (specify) ________________

*****

Thank you for your willingness to take the time to answer this survey.
