# Supplementary material for: Catalyzing sustainable fisheries management through behavior change interventions
Source: Conserv Biol. 2020 Apr 15;34(5):1176–89. doi: 10.1111/cobi.13475 (PMC7540413; doi:10.1111/cobi.13475)
Supplement: Supplementary file 9 — Supplementary Material [file COBI-34-1176-s009.docx]

Preparation (completed by Enumerator / Committee)

No. Questionnaire

________________

Name of Enumerator

________________

Day / date of the interview

________________

Name of interview location

[] Gili Trawangan [] Gili Meno [] Gili Air

Survey Period:

[] Pre-Campaign - Intervention [] Post Campaign - Intervention [] Pre Campaign - Comparison [] Post Campaign - Comparison

PAAP TWP Gili Matra Questionnaire

Introduction

Good morning / afternoon / afternoon

We << mention the name of the self and the name of the institution >> - intend to conduct research on << write the name of research and location >> The purpose of this study is << write the purpose of data usage >>.

The survey consists of XX statements, which I will read to you. Please be willing to Mr / Mrs to respond to this statement. This interview can be completed in approximately 40 minutes. Given the importance of this information, we hope that you are willing to answer the questions in this survey. There is no wrong and correct answer. Honesty and openness of Father / Mother is very important in providing this information. Answers Mr / Mrs will only be known by us, as researchers.

Have you ever been interviewed before?

[] Already (end the interview and say thanks) [] Not yet (continue interview)

Will you be interviewed?

[] No (end the interview and say thanks) [] Yes (continue the interview)

SELF INFORMATION

I will read some statements about you. Please kindly give us the answer that best suits you. There is only one answer for each question.

(1) Gender (filled directly by Enumerator)

[] Women [] Men

(2) What is your current age?

[] Under or equal to 17 years [] 18-24 years [] 25-31 years [] 32 - 38 years [] 39 - 45 years [] 46 - 52 years [] Above or equal to 53 years old

(3) Mention the last level of education Mr / Ms

[] Never graduated [] Did not finish elementary school [] Graduated from elementary school / equivalent [] Junior High or equivalent [] High School graduate / equal [] Others (specify) ________________

(4) What is your main job?

[] Full-time fisherman (continued to AH) [] Part-time fisherman (continued to AH) [] Business owner / manager dive shop / boat trip [] dive shop operator [] Captain boat [] Others (please specify) [] Others (specify) ________________

(5) What side job Mr / Mrs:

[] Labor [] Tour [] Gardening [] Coachman cidomo [] Fisherman [] Others (specify) ________________

If the respondent is a full-time or part-time fisherman, go to question A - J. Respondents who choose other than directly ask K questions

Self Information for Full-time Fishermen and Part Time Fishers

(A) If you choose a full-time / part-time fisherman: what kind of fish do you catch most? (Show image)

[] Lutjanus bohar [] Lutjanus gibbus [] Lutjanus bohar and Lutjanus gibbus [] Others (specify) ________________

(B) What fishing equipment is used?

[] Fishing Line [] Dengkol Nets [] Sret Nets [] Supply Nets [] Spider Nets [] Arrows [] Others (specify) ________________

(C) When did you catch a fish in the sea?

[] Morning [06.00 - 12.00] [] Afternoon [12.00 - 18.00] [] Night [22.00 - 05.00]

(D) In ​​conducting fishing activities, using what kind of ship are you or your group?

________________

(E) If you / your group are using a ship, specify the size of the PK engine that your ship / group you are using

________________

(F) The average costs incurred for fishing each time to go to sea are:

[] Under Rp 250,000 [] Rp 250,000 - Rp 500,000 [] Rp 500,000 - Rp 1,000,000 [] Above Rp 1,000,000

(G) Your source of capital for fishing, usually derived from:

[] Self / family [] tauke / collector [] cooperative [] borrow friend / neighbor [] Others (specify) ________________

(H) Within a month, you usually do sea activities to find as many fish

[] Less than 4 times in a month [] 5-20 times per month [] 21 - 36 times per month [] more than 36 times per month [] uncertain

(I) In 1 month, how did your catch compare to the same month last year?

[] Same [] More [] More and more bigger [] Less [] Uncertain [] Do not remember / do not know

(J) In this 1 month, to get the same number of fish as last year, your fishing distance:

[] Same course [] Closer than last year [] Farther than last year [] Not sure [] Do not remember

Go to question number 6.

Self Information for Respondents In addition to Full-time Fishermen and Part Time Fishers

(K) In one trip / boat how many guests?

[] Less than 10 people [] 10-20 people [] Above 20 people

(L) If you are a dive shop operator / boat captain trip: how many times a day brings guests?

[] 1-2 times [] 3-4 times [] More than 4 times

(M) When is the time? (Respondents may answer more than 1)

[] Morning 06.00-12.00 [] Afternoon 12.00-18.00 [] Night 22.00-05.00

Go to question number 6.

(6) What is the number of your family members in one house (including yourself)

[] 1 person (just yourself) [] 2 people [] 3 people [] 4 people [] equal or more than 5 people

(7) Average monthly expenditure of your family is:

[] Under Rp 500,000 [] Rp 500,000 - Rp 1,500,000 [] Rp 1,500,000 - Rp 2,500,000 [] above Rp 2,500,000

FISHERY MANAGEMENT

Here are two questions about fisheries management. Please feel free to give the best answer according to your opinion.

(8) In your own words, please explain what is meant by Area Fisheries Access Management. (If the respondent answers "Not Know", write "Do not Know")

________________

(9) State all existing rules for management of fishery area access

________________

DAILY HABITS IN SEARCHING AND MANAGING SEA MARKETS

Here are some statements about the habits of finding and managing seafood. Please kindly give your answer in accordance with the habits and beliefs of Mr / Ms.

(10) Other people in this village, who set an example for me to do activities in accordance with the Gili Matra TWP zoning rules are:

[] Chairman of the group [] Chairman of the fisherman group [] Chairman of the cooperative [] Tengkulak [] Fellow fisherman [] Chairman of Mosque / Church [] Wife / husband [] Child [] None [] Do not know [] Others (specify) ________________

(11) Anyone in this village who requires me to do activities in accordance with TWP Gili Matra zoning rules is

[] Chairman of the group [] Chairman of the fisherman group [] Chairman of the cooperative [] Tengkulak [] Fellow fisherman [] Chairman of Mosque / Church [] Wife / husband [] Child [] None [] Do not know [] Others (specify) ________________

For the statement below, please state your answer, with "Yes", "No", or 'Can not remember'

(12) In the last 6 months, I talked to others about:

(A) benefits gained from the management of the fishery area access

[] Yes [] No [] Do not remember

(B) compliance with applicable rules within the territory of the fishery access area

[] Yes [] No [] Do not remember

(C) ways of monitoring and reporting violations of rules in the area of ​​access of the fishing area

[] Yes [] No [] Do not remember

Here, please tell me whether 'easy,' rather easy ',' hesitant ',' rather difficult ', difficult' to do things yourself in this statement.

(13) For me,

(A) not looking for fishing / doing tourism activities in the Core Zone

[] Easy [] Somewhat easy [] Hesitant [] Somewhat difficult [] Difficult

(B) complies with the rules of access management of the fishing area

[] Easy [] Somewhat easy [] Hesitant [] Somewhat difficult [] Difficult

(C) engage in processes and discussions for the management rules of the fishery area access

[] Easy [] Somewhat easy [] Hesitant [] Somewhat difficult [] Difficult

(D) report the catch

[] Easy [] Somewhat easy [] Hesitant [] Somewhat difficult [] Difficult

(E) report a violation of the rules in the area of ​​access of the fishery area

[] Easy [] Somewhat easy [] Hesitant [] Somewhat difficult [] Difficult

(F) invites others to comply with the management rules of the fishery area access

[] Easy [] Somewhat easy [] Hesitant [] Somewhat difficult [] Difficult

Here, please Mr / Mrs declare whether 'Agreed', 'Disagree', 'Do not know' to the statement below

(14) To me, adhere to the rules of access management of the fishing area

(A) is a form of responsibility as a community in the region

[] Agree [] Disagree [] Do not know

(B) can guarantee the continuation of my family's life in the future

[] Agree [] Disagree [] Do not know

(C) can keep the availability of fish and other marine resources for a long time

[] Agree [] Disagree [] Do not know

(D) can preserve the traditions of life as fishermen from generation to generation

[] Agree [] Disagree [] Do not know

(15) To me, obey the rules of management of fishery area access

(A) will incur additional costs to replace the work tool

[] Agree [] Disagree [] Do not know

(B) can not be done because there is no firmness against rule violators

[] Agree [] Disagree [] Do not know

(C) can not be executed because there is no visible border for the location of the fishery area access

[] Agree [] Disagree [] Do not know

(D) makes the time to go on longer because of having to report the catch (or something else)

[] Agree [] Disagree [] Do not know

(E) will reduce revenue

[] Agree [] Disagree [] Do not know

Here's what you want to do, 'Somewhat sure to be able to do', 'Doubtful', 'Somewhat unsure of being able to do', 'Unsure able to do' the following statements.

(16) I feel,

(A) does not infringe on the Core Zone

[] Sure able to do [] Somewhat sure able to do [] Hesitant [] Somewhat unsure able to do [] Not sure able to do

(B) catch fish according to the rules in the area of ​​fishery access area

[] Sure able to do [] Somewhat sure able to do [] Hesitant [] Somewhat unsure able to do [] Not sure able to do

(C) using the type of fishing gear permitted in the area of ​​fishery access area

[] Sure able to do [] Somewhat sure able to do [] Hesitant [] Somewhat unsure able to do [] Not sure able to do

(D) reporting of fish catch

[] Sure able to do [] Somewhat sure able to do [] Hesitant [] Somewhat unsure able to do [] Not sure able to do

(E) supervise and report violations in the area of ​​access of the fishing area

[] Sure able to do [] Somewhat sure able to do [] Hesitant [] Somewhat unsure able to do [] Not sure able to do

(F) conduct tourism activities according to the rules in the area of ​​fishery access area

[] Sure able to do [] Somewhat sure able to do [] Hesitant [] Somewhat unsure able to do [] Not sure able to do

(G) periodically reporting the number of guests / tourists

[] Sure able to do [] Somewhat sure able to do [] Hesitant [] Somewhat unsure able to do [] Not sure able to do

(17) (Enumerator provides a map and gives an explanation of how to read the map to the respondent Enumerator then fill in answer according to the accuracy / inaccuracy of respondents).

The enumerator read this question to the respondent:

From this map, point to / mention all the locations you usually do activities (eg fishing or tourism activities)

(Enumerator: Writing all respondent's answer If not willing to answer write 'No answer')

________________

(A) Based on the location of the above mentioned fishing / fowl, please Mr / Mrs choose the statement that best describes you right now

[] I do not know the designation rules for this area and do not think to find out [] I do not know the designation rules for this area but have been thinking about finding out [] I am not implementing the designation rules for this area but in the near future I think to do it [] I have followed the designation rules for this area, but only implemented it for less than 6 months [] I have followed the rules of the designation of this area and have done so in 6 months or more

For the following statement, please choose the one that best describes you right now

(18) For the following statement, please choose the one that best describes you right now

[] I do not know the rules about fishing gear allowed in the area of ​​access area and do not think to find out [] I do not know the rules of fishing gear are allowed in the area of ​​access area but in the near future thought to find out [] I already know fishing gear Which is allowed in the area of ​​access area and in the near future it is thought to do it [] I have been using the type of fishing gear according to the rules in the area of ​​access area, for less than 6 months [] I have used the type of fishing gear that match the rules of area access area, 6 months or more

(19) For the following statement, please choose the one that best describes you right now

[] I do not know the rules about the size of the catch in the area of ​​access area and do not think to find out [] I do not know the catch size rules in the area of ​​access area but in the near future it is thought to find out [] I already know the size of the catch allowed in the region Access area and in the near future think to do it [] I have caught fish with the size of fish catch according to the rules in the area of ​​access area, and have been doing it for less than 6 months [] I have captured the size of the fish according to the rules of area access area and have done it in 6 Months or more

(20) For the following statement, please choose the one that best describes you right now

[] I do not know the rules of the number of guests / trips allowed in the territory of the fishery area and do not think to find out [] I do not know the rules of the number of guests / trips allowed in the fishery area access area but in the near future it is thought to find out [] I already know the rules of the number of guests / trips allowed in the fishing area access area and in the near future it is thought to do it [] I already know about the number of guests / trips allowed in the fishing area access area, and have done it less than 6 months [] I Already know about the rules of the number of guests / trips allowed in the fishing area access areas and have done so in 6 months or more

(21) For the following statement, please choose the one that best describes you right now

[] I did not participate in the management of area access areas and did not think to do it [] I did not participate in the management of area access areas but have thought to find out [] I have thought about participating in the management of area access areas in the near future [] I have Participate in the management of area access areas, but only implement them for less than 6 months [] I have participated in the management of area access areas and have done so in 6 months or more

(22) What activities do you think are the most effective way of passing information about PAAP here?

[] Art festivals [] Fishing meetings [] Religious activities [] Nothing effective [] Others (specify) ________________

(23) What activities do you think most effectively give information about the fishery management rules here?

[] Art festivals [] Fishing meetings [] Religious activities [] Nothing effective [] Others (specify) ________________

(24) What media do you think is the most effective way of passing information about PAAP here?

[] Stall banner [] Poster [] Calendar [] Nothing effective [] Others (specify) ________________

(25) What media do you think is the most effective way to convey information about fishery rules here?

[] Stall banner [] Poster [] Calendar [] Nothing effective [] Others (specify) ________________

*****

Thank you for your willingness to take the time to answer this survey.
