# Supplementary material for: Catalyzing sustainable fisheries management through behavior change interventions
Source: Conserv Biol. 2020 Apr 15;34(5):1176–89. doi: 10.1111/cobi.13475 (PMC7540413; doi:10.1111/cobi.13475)
Supplement: Supplementary file 11 — Supplementary Material [file COBI-34-1176-s011.docx]

Preparation (completed by Enumerator / Committee)

No. Questionnaire ________________ Enumerator ________________

Day / date of interview ________________ Name of interview location: Village ________________

Survey period: [] Pre Campaign - Intervention [] Post Campaign - Intervention

**Introduction**

Good morning / afternoon. We << name and the institution >> - intend to gather information on marine management and fisheries resources in the region. The purpose of gathering this information is to have a complete picture of coastal community here and fisheries related issues in the community.

This survey consists of 17 questions, which I will read to you. The time required to complete this survey is about 25 minutes. Given the importance of information from you, we hope that you are willing to answer the questions in this survey honestly. There is no wrong and correct answer, so honesty and openness is very important in every given response.

Have you ever been interviewed before?

[] Already (end the interview and say thanks) [] Not yet (continue interview)

Will you be interviewed?

[] No (end the interview and say thank you) [] Yes (continue the interview)

SELF INFORMATION

(1) Gender (filled directly by Enumerator) [] Female [] Male

I will read some questions about you. Please kindly give us the answer that best suits you. There is only one answer for each question.

(2) What is your current age? ________________

(3) Mention your last level of education

[] Never go to school [] Do not finish elementary school 
[] Graduate School Elementary / equivalent [] Graduated from Junior High School / equivalent 
[] High School or equivalent [] Others (specify) ________________

(4) What is your main job?

[] Full-time fisherman (go to AH) [] Part-time / seasonal fisherman (go to AH) 
[] Civil servants (continued to No. 5) [] Not working anyway (go to No. 5) 
[] Others (specify) ________________

(A) Answer the following questions:

Type of fish MOST often captured ________________

The types of fishing gear used to fish the most commonly caught fish ________________

Longest time of catch (average in hours) to fish the most commonly caught fish _____________

(B) Average costs incurred (in rupiah) to fish each time you go to sea are: (the cost of going to sea is fuel, food, cigarettes, bait, additional fishing gear) _______________

(C) How many days a week do you usually go to fish?

[] 1 times [] 2 times [] 3 times [] 4 times [] 5 times [] 6 times [] 7 times [] uncertain

(D) Where did you get your capital to fish from?

[] Own / family [] basket / collectors [] cooperative [] borrow friends 
[] Others (specify) ________________

(E) How is your catch this year compared to last year in the same month?

[] Same [] More [] More and more 
[] Less [] Uncertain [] Do not remember / do not know

(F) In order to fish the same amount of fish this month as last year's catch, is there a difference in the distance in doing the fishing activities?

[] Same [] Closer than last year [] Farther than last year 
[] Not sure [] Do not remember

(G) You think that fishing can provide for the needs of you and your family for present and future time

[] Strongly Agree [] Agree [] Ordinary / Neutral [] Disagree [] Strongly disagree

(H) Other people in this village who set an example for you to look for fish according to the rules are

[] Fellow fisherman [] Other family members [] Skipper / owner ship 
[] Collectors / collectors [] None [] Others (specify) ________________

(5) What is the number of your family members in one household (including yourself)

[] Only 1 person (yourself) [] 2 persons [] 3 people 
[] 4 people [] 5 people or more

(6) What is your family's average monthly expenditure on monthly basis:

(Such expenditures include meals, clothing, cigarettes, fishing gear, fuel, telephone credit, tuition, medical expenses, etc.) ________________

FISHERY MANAGEMENT

Here are two questions about fisheries management. Please feel free to give the best answer according to your opinion.

(7) Have you heard or heard of the Mosquito Village Management Area (KPDN)?

[] Never **[STOP INTERVIEW AND SAY THANK YOU]** 
[] Never [go to next question]

(8) In your own words, please explain what you know about the Mosquito Village Management Area (KPDN). (Write the respondent's answer here)

DAILY HABITS IN SEARCHING AND MANAGING SEA MARKETS

Here are some questions about the habits of finding and managing seafood. Please kindly give your answer in accordance with the habits and beliefs of Mr / Mrs.

For the questions below, please provide your answer, "Yes", "No", or 'Can not remember'

(9) In the last 6 months, Mr / Ms talked with other fishermen about:

(A) the benefits derived from the KPDN

[] Yes [] No [] Do not remember

(B) does not catch fish in tourism utilization zones

[] Yes [] No [] Do not remember

(C) does not shoot fish on 18-28 Hijriah every month

[] Yes [] No [] Do not remember

(D) do not throw garbage into the sea

[] Yes [] No [] Do not remember

(E) does not catch sunu size less than 35 cm

[] Yes [] No [] Do not remember

Here, please tell me whether 'easy,' rather easy ',' hesitant ',' rather difficult ', difficult' to do things yourself in this statement.

(10) For Mr / Ms,

(A) does not catch fish in the tourism utilization zone

[] Easy [] Somewhat easy [] Hesitant [] Somewhat difficult [] Difficult

(B) comply with KPDN rules

[] Easy [] Somewhat easy [] Hesitant [] Somewhat difficult [] Difficult

(C) engage in processes and discussions for KPDN rules

[] Easy [] Somewhat easy [] Hesitant [] Somewhat difficult [] Difficult

(D) report on the catch from inside and outside KPDN

[] Easy [] Somewhat easy [] Hesitant [] Somewhat difficult [] Difficult

(E) reported violations of the rules in the KPDN region

[] Easy [] Somewhat easy [] Hesitant [] Somewhat difficult [] Difficult

(F) invites fellow fishermen to comply with KPDN rules

[] Easy [] Somewhat easy [] Hesitate [] Somewhat difficult [] Difficult

(G) does not catch fish on 18-28 Hijriah every month

[] Easy [] Somewhat easy [] Hesitant [] Somewhat difficult [] Difficult

(H) do not throw garbage into the sea

[] Easy [] Somewhat easy [] Hesitant [] Somewhat difficult [] Difficult

(I) did not catch the sunu size less than 35 cm

[] Easy [] Somewhat easy [] Hesitant [] Somewhat difficult [] Difficult

(11) (Enumerator provides a map with the name of the area and gives an explanation of how to read the map to the respondent Information can be given such as, direction of the wind, village position, and scale Enumerator then overwrite the OHP map and fill in answer according to the answers given by the respondent ). The enumerator read this question to the respondent:

11.a. From this map, point to / mention all the locations you usually go looking for fish

(Enumerator: Writing all respondent's answer If not willing to answer write 'No answer')

[] In the tourism utilization zone 
[] Beyond KPDN [] Outside of tourism utilization zone but inside KPDN zone 
[ ] No answer

11.b. From this map, point to / name your location usually go to fish in the calendar period of Hijri

Enumerator: write all respondents' answers. If you are not willing to answer write 'No answer'}

1 - 9 Hijri calendar

[] In [] Outside [] No answer

10 - 17 Hijri calendar

[] In [] Outside [] No answer

18 - 28 Hijri calendar

[] In [] Outside [] No answer

For the following statement, please choose the one that best describes you right now

(12) For the following statement, please Mr / Mrs choose that best describes yourself Mr / Ms at this time

[] I do not know the rules about fishing gear being allowed in KPDN area and do not think to find out

[] I do not know the rules of fishing gear that is allowed in KPDN area but in the near future think to find out

[] I already know the permissible fishing gear in KPDN area and but have not thought to obey it

[] I already know the permissible fishing gear in KPDN area and in the near future I think to obey it

[] I have been using the kind of fishing gear according to the rules in the KPDN area, for less than 6 months

[] I have been using a kind of fishing gear which is in accordance with KPDN area rules, within 6 months or so

[ ] No answer

(13) For the following statement, please Mr. / Mrs. choose that best describes yourself Mr / Ms at this time

[] I do not know the rules about the size of the catch in the KPDN area and do not think to find out

[] I do not know the catch size rule in KPDN area but in the near future I think to find out

[] I already know the size of the catch that is allowed in KPDN area but have not yet thought to obey it

[] I already know the size of the catch that is allowed in KPDN area and in the near future thought to obey it

[] I have caught fish with the size of fish catch according to the rules in KPDN region, and have been doing it less than 6 months

[] I have captured the size of the fish according to KPDN region rules and have done it in 6 months or more

[ ] No answer

(14) For the following statement, please Mr. / Mrs. choose that best describes yourself Mr / Ms at this time

[] I was never involved in KPDN surveillance and did not think of doing it

[] I was never involved in KPDN surveillance and thought to find out

[] I am thinking to be involved in the monitoring of KPDN area, in the near future

[] I have been involved in KPDN area surveillance, less than 6 months

[] I have been involved in the supervision of the KPDN area, within 6 months or more

[ ] No answer

(15) For the following statement, please Mr / Mrs choose that best describes yourself Mr / Ms at this time

[] I do not participate in the management of the KPDN region and do not think to look for ways to get involved

[] I did not participate in KPDN area management but have thought to find out how to get involved

[] I know about the management of the KPDN area but I have not participated or actively engaged in reporting

[] I am preparing to participate or participate in the management of KPDN areas

[] I have participated in the management of the KPDN area, but only implemented it for less than 6 months

[] I have participated in the management of the KPDN area and have done so in 6 months or more

[ ] No answer

***** Thank you for your willingness to take the time to answer this survey *****
