# Supplementary material for: Catalyzing sustainable fisheries management through behavior change interventions
Source: Conserv Biol. 2020 Apr 15;34(5):1176–89. doi: 10.1111/cobi.13475 (PMC7540413; doi:10.1111/cobi.13475)
Supplement: Supplementary file 13 — Supplementary Material [file COBI-34-1176-s013.docx]

No. Questionnaire

________________

Name of Enumerator

________________

Name of Respondent

________________

Day / date of the interview

________________

Name of interview location

[] Gampong Kuta Ateuh [] Gampong Ie Meulee

Survey Period:

[] Pre Campaign - Intervention [] Post Campaign - Intervention

OPINIONAL POLICY ON THE MANAGEMENT OF FISHERIES AREAS ACCESS IN KKPD SABANG

Introduction

Good morning / afternoon / afternoon

Currently I am assisting DKP Sabang to conduct research on the Management of Fishery Area Access (PAAP) in the Regional Water Conservation Area (KKPD) of Sabang. The purpose of this research is to know the opinion of Mr. / Abang about it.

This survey consists of 27 statements, which I will read to Mr. / Abang. Please kindly give your response to this question. This interview can be completed in approximately 40 minutes. Given the importance of this information, we hope that Mr. / Abang is willing to answer the questions in this survey. There is no wrong and correct answer. Honesty and openness of Mr. / Abang is very important in providing this information. Your answer will only be known to us, as a researcher.

Have you ever been interviewed before?

[] Already (end the interview and say thanks) [] Not yet (continue interview)

Will you be interviewed?

[] No (end the interview and say thanks) [] Yes (continue the interview)

SELF INFORMATION

I will read some questions about you. Please kindly give us the answer that best suits you. There is only one answer for each question.

(1) What is the current age of Mr. / Abang?

[] Under or equal to 17 years [] 18-24 years [] 25-31 years [] 32 - 38 years [] 39 - 45 years [] 46 - 52 years [] Above or equal to 53 years old

(2) Mention the last education of Mr. / Abang

[] Never graduated [] Did not finish elementary school [] Graduated from elementary school / equivalent [] Graduated from junior high school / equivalent [] graduated high school / equivalent [] Others (specify) ________________

(3) What is the main job of Mr. / Abang?

[] Full-Time Fisherman [] Fishermen Part Time [] Others (specify) ________________

(4) What kind of fish do you most often Father / Abang catch?

[Grouper Grouper] Grouper [Grouper Grouper] Grouper [Grouper Grouper] Pomegranate Grouper [] Grouper Gudang Garam [] Banana [] Spinach [] Cuttlefish [] Octopus [] Itam Kahap [] Snapper [] Bracung [] The beard [] Seedang [] Red eye [] Others (specify) ________________

(5) Using what fishing tools?

[] Basic Fishing [] Tonda Fishing [] Longline Fishing [] Spearguns [] Others (specify) ________________

(6) When is the time?

[] 08.00 - 14.00 Wib [] 14.00 - 20.00 Wib [] 20.00 - 08.00 Wib [] Others (specify) ________________

(7) The average costs incurred for fishing each time to go to sea are:

[] Below Rp 100.000 [] Same as Rp. 100.000 [] Above Rp. 100,000

(8) Within a month, do you usually catch fish how many times?

[] Less than 26 times in a month [] equal to 26 times in a month [] more than 26 times in a month

(9) the source of capital to go to sea is obtained from:

[] Own / family [] toke bench [] cooperative [] borrow friend [] government aid [] Others (specify) ________________

(10) Within a month, how did your catch / catch compared to the same month last year?

[] Same [] More [] More and more bigger [] Less [] Uncertain [] Do not remember / do not know [] More and smaller [] smaller [] Others (specify) ________________

(11) In this 1 month, to get the same amount of fish as last year, the distance to sea /

[] Same course [] Closer than last year [] Farther than last year [] Uncertain [] Do not remember [] Others (specify) ________________

(12) What is the number of family members of Father / Abang in one house (including yourself / Father / Self)?

[] 1 person (just yourself) [] 2 people [] 3 people [] 4 people [] equal or more than 5 people

(13) The average family expenditure of Mr. / Abang per month is:

[] Under Rp. 2.000.000, - [] Same as Rp. 2.000.000, - [] More than Rp. 2,000,000, -

FISHERY MANAGEMENT

Here are two questions about fisheries management. Please feel free to give the best answer according to your opinion.

(14) In your own words, please explain what is meant by Area Fisheries Access Management (PAAP). (If the respondent answers "Not Know", write "Do not Know")

________________

(15) State all existing rules for the Fishery Area Access Management (PAAP)

________________

DAILY HABITS IN SEARCHING AND MANAGING SEA MARKETS

Here are some questions about the habits of finding and managing seafood. Please kindly give your answer in accordance with the habits and beliefs of Mr / Ms.

(16) Other people in this gampong, who require you to fish according to the rules are:

[] Panglima Laot Lhok Ie Meulee (Yah Ngah) [] Toke Bangku / Mugee [] Fellow Fisherman [] Bang Abbas [] Obi [] Pak Budiono [] Bang Deh / Anis [] Bang Sani [] Untung Aaron [] Azhar [] Pak Sanusi [] Mr. Sunyoto [] Pak Farid Alatas [] Bang Suryansyah [] Others (specify) ________________

(17) Other people in this gampong, who serve as an example for Bapak / Abang to find fish according to the rules are:

[] Panglima Laot Lhok Ie Meulee (Yah Ngah) [] Toke Bangku / Mugee [] Fellow Fisherman [] Bang Abbas [] Obi [] Pak Budiono [] Bang Deh / Anis [] Bang Sani [] Untung Aaron [] Azhar [] Pak Sanusi [] Mr. Sunyoto [] Pak Farid Alatas [] Bang Suryansyah [] Others (specify) ________________

For the statement below, please state your answer, "Yes", "No" or 'Can not remember'

(18) In the past month, have you talked to other fishermen about:

(A) benefits gained from the management of the fishery area access

[] Yes [] No [] Do not remember

(B) compliance with applicable rules within the territory of the fishery access area

[] Yes [] No [] Do not remember

(C) ways of monitoring and reporting violations of rules in the area of ​​access of the fishing area

[] Yes [] No [] Do not remember

Here, please Bapak / Abang declare whether 'easy,' rather easy ',' hesitant ',' rather difficult ', difficult' to do things yourself in this statement.

(19) According to Mr. / Abang, how difficult is the level of things below to do?

(A) not looking for fish in the area of ​​Prohibited Area Take Home The Point Resort - Cassanemo

[] Easy [] Somewhat easy [] Hesitant [] Somewhat difficult [] Difficult

(B) complies with the rules of access management of the fishing area

[] Easy [] Somewhat easy [] Hesitant [] Somewhat difficult [] Difficult

(C) engage in processes and discussions for the management rules of the fishery area access

[] Easy [] Somewhat easy [] Hesitant [] Somewhat difficult [] Difficult

(D) report the catch

[] Easy [] Somewhat easy [] Hesitant [] Somewhat difficult [] Difficult

(E) report a violation of the rules in the area of ​​access of the fishery area

[] Easy [] Somewhat easy [] Hesitant [] Somewhat difficult [] Difficult

(F) invites fellow fishermen to comply with the management rules of fisheries area access

[] Easy [] Somewhat easy [] Hesitant [] Somewhat difficult [] Difficult

Here, please Bapak / Abang declare whether 'Agreed', 'Disagree', 'Do not know' to the statement below

(20) According to Mr. / Abang, adhere to the rules of access management of the fishery area

(A) is a form of responsibility as a fisherman in the region

[] Agree [] Disagree [] Do not know

(B) ensuring the continuation of my family's life in the future

[] Agree [] Disagree [] Do not know

(C) maintaining the availability of fish and other marine resources for a long time

[] Agree [] Disagree [] Do not know

(D) preserves the traditions of life as fishermen from generation to generation

[] Agree [] Disagree [] Do not know

(21) According to Mr. / Abang, adhere to the rules of access management of the fishery area

(A) will incur additional costs to replace fishing gear

[] Agree [] Disagree [] Do not know

(B) can not be done because there is no firmness against rule violators

[] Agree [] Disagree [] Do not know

(C) can not be executed because there is no visible border for the location of the fishery area access

[] Agree [] Disagree [] Do not know

(D) makes the time to go to sea longer due to reporting the catch

[] Agree [] Disagree [] Do not know

Here's what you want to do, 'Somewhat sure to be able to do', 'Doubtful', 'Somewhat unsure of being able to do', 'Unsure able to do' the following statements.

(22) I feel,

(A) do not catch fish in the Forbidden Area Take the front of The Point Resort-Cassanemo

[] Sure able to do [] Somewhat sure able to do [] Hesitant [] Somewhat unsure able to do [] Not sure able to do

(B) catch fish according to the rules in the area of ​​fishery access area

[] Sure able to do [] Somewhat sure able to do [] Hesitant [] Somewhat unsure able to do [] Not sure able to do

(C) using the type of fishing gear permitted in the area of ​​fishery access area

[] Sure able to do [] Somewhat sure able to do [] Hesitant [] Somewhat unsure able to do [] Not sure able to do

(D) reporting the catch

[] Sure able to do [] Somewhat sure able to do [] Hesitant [] Somewhat unsure able to do [] Not sure able to do

(E) supervise and report violations in the area of ​​access of the fishing area

[] Sure able to do [] Somewhat sure able to do [] Hesitant [] Somewhat unsure able to do [] Not sure able to do

(23) (Enumerator provides maps and explains how to read maps to respondents Enumerators then fill in answers according to the accuracy / inaccuracy of respondents).

The enumerator read this question to the respondent:

From this map, point to / mention all the locations you usually go looking for fish

(Enumerator: Writing all respondent's answer If not willing to answer write 'No answer')

________________

(A) Based on the location of the above mentioned fishing / abang fishery, please Mr. / Abang select the statement that best describes you right now

[] I do not know the rules for PAAP location and do not think to find out [] I do not know the rules for PAAP location and have thought to find out [] I do not know the rules for PAAP location and have thought to find out [] I have run Rules for PAAP locations within 6 months [] I have run the rules for PAAP locations in 6 months or more

For the following statement, please select the one that best describes you right now

(24) For the following statement, please select the one that best describes you right now

[] I do not know the rules about fishing gear allowed in PAAP area and do not think to find out [] I do not know the rules of fishing gear allowed in PAAP area but in the near future I think to find out [] I already know the fishing gear is allowed In the PAAP area and in the near future it is possible to do so [] I have been using the appropriate type of fishing gear in the PAAP area, for less than 6 months [] I have been using the appropriate type of fishing gear in PAAP, within 6 months or more

(25) For the following statement, please select the one that best describes you right now

[] I do not know the rules about the size of the fish catch in PAAP area and do not think to find out [] I do not know the fishing catch size rule in PAAP area but in the near future I think to find out [] I already know the size of the fish catch that is allowed in PAAP region and in the near future thought to do it [] I have caught fish with the size of the fish catch according to the rules in the PAAP region, and have been doing it for less than 6 months [] I have captured the appropriate fish size in the PAAP area and have done it in 6 Months or more

(26) For the following statement, please select the one that best describes you right now

[] I did not participate in the Fisheries Access Management (PAAP) and did not think to do it [] I did not participate in the Fisheries Area Access Management (PAAP) but have thought to find out [] I have thought about participating in the Area of ​​Fisheries Access Management PAAP) in the near future [] I have participated in the Fisheries Area Access Management (PAAP), but only implemented it for less than 6 months [] I have participated in the PAAP and have done so in 6 months or more

(27) For the following statement, please select the one that best describes you right now

[] I have never been involved in PAAP surveillance and have not thought of doing it [] I have never been involved in PAAP surveillance and thought to find out [] I was thinking about getting involved in PAAP territorial supervision in the near future [] I was already involved in territorial surveillance PAAP, less than 6 months [] I have been involved in PAAP area surveillance, within 6 months or more

MEDIA EFFECTIVENESS

(28) What activities do you think most effectively convey information about PAAP here?

[] Art festivals [] Fishermen's meetings [] Religious activities [] Nothing effective [] Others (specify) ________________

(29) What activities do you think most effectively convey information about fisheries management rules here?

[] Art festivals [] Fishing meetings [] Religious activities [] Nothing effective [] Others (specify) ________________

(30) What media do you think are most effective in conveying information about PAAP here?

[] Stall banner [] Poster [] Calendar [] Nothing effective [] Others (specify) ________________

(31) What media do you think are most effective in conveying information about fishery rules here?

[] Stall banner [] Poster [] Calendar [] Nothing effective [] Others (specify) ________________

*****

Thank you for your willingness to take the time to answer this census.
