# Supplementary material for: Catalyzing sustainable fisheries management through behavior change interventions
Source: Conserv Biol. 2020 Apr 15;34(5):1176–89. doi: 10.1111/cobi.13475 (PMC7540413; doi:10.1111/cobi.13475)
Supplement: Supplementary file 14 — Supplementary Material [file COBI-34-1176-s014.docx]

Preparation (completed by Enumerator / Committee)

No. Questionnaire

________________

Name of Enumerator

________________

Day / date of the interview

________________

Name of Respondent

________________

Name of interview location

[] Coconut Island [] Pulau Kelapa Dua [] Island of Hope

Survey Period:

[] Pre-Campaign - Intervention [] Post Campaign - Intervention [] Pre Campaign - Comparison [] Post Campaign - Comparison

The Papa Island Fishermen Polls, Kelapa Dua Island and Hope Island Concerning the Management of Fishery Area Access (PAAP) in TNKpS

Introduction

Good morning / afternoon / afternoon

I << name your self >> - intends to hold a poll on the Fishery Area Access Management program located in the waters of Pengagaran Island, Pulau Panjang Besar and Pulau Panjang Kecil. The purpose of this poll to know things related to fisheries in the area of ​​Thousand Islands National Park.

This poll consists of 23 questions / statements that I will read to you. Please kindly respond to this question / statement. This interview can be completed approximately 40 minutes. There is no wrong and correct answer. Honesty and openness are very important in this poll. Your answer will only be known to us, as a researcher.

Have you ever been interviewed before?

[] Already (end the interview and say thanks) [] Not yet (continue interview)

Are you willing to be interviewed?

[] No (end the interview and say thanks) [] Yes (continue the interview)

SELF INFORMATION

I will read some questions about you. Please give the answer that best suits you. There is only one answer for each question.

(1) What is your current age?

[] 18 - 24 years [] 25- 31 years [] 32 - 38 years [] 39 - 45 years [] 46 - 52 years [] 52 years of age

(2) Mention your last education level

[] Has never graduated [] Has not graduated SD / SR [] Graduated from Junior High / equivalent [] Has completed junior high school / equivalent [] High School graduation / equivalent [] Not Graduate High School / equivalent [] Others (specify) ________________

(3) Are you included?

[] Full-time fisherman [] Fishermen part-time

(A) The most common type of fish caught:

[] Coral squid [] Yellow tail [] Coral squid and yellow tail [] Others (specify) ________________

(B) The type of fishing equipment used

[] Fishing Rods [] Squid Fishing [] Nets and Fishing Rods [] Squid Nets and Fishing Rod [] Others (please specify) ________________

(C) Fishing time:

[] At 7 am - 4 pm [] 4 pm - 2 am [] 6 pm-1 am (night fishing) [] Others (specify at what time)

(D) The average costs incurred for fishing each time to go to sea are:

[] Rp 100 rb - Rp 500 rb - Rp 500 rb [] Rp 500 rb - Rp 1 jt [] Above Rp 1 million

(E) In a week, you usually go out to sea to fish as much as:

[] 1-2 days [] Everyday [] Everyday except Friday [] Not sure

(F) Your source of capital for fishing, usually obtained from:

[] Own / family [] Borrow to Pelele [] Borrow to fellow fishermen [] Others (specify) ________________

(G) How did you catch this month compared to the same month last year?

[] Same [] More [] More and more bigger [] Less [] Uncertain [] Do not remember / do not know

(H) How is your fishing distance in this month compared to last year to get the same result?

[] Same course [] Closer than last year [] Farther than last year [] Not sure [] Do not remember

(4) How many members of your family in one house (including yourself)

[] 1 person (only yourself) [] 2 people [] 3 people [] 4 people [] equal or more than 5 people

(5) The average monthly expenditure of your family is:

[] Rp. 500 rb - Rp. 1 jt [] Rp. 1 jt - Rp. 1.5 jt [] Rp. 1.5 jt - 2 jt [] Rp. 2M more

FISHERY MANAGEMENT

Here are two questions about fisheries management. Please kindly give the best answer according to your opinion.

(6) In your own words, please explain what is meant by Area Access Area Management (PAAP). (If the respondent answers "Do not Know", write "Do not Know")

________________

(7) Do you know or do not know about PAAP agreement in TNKpS? (If the respondent answers "do not know", go to question number 8. If you answered "know", go to question 7A)

[] Know [] Do not know

(A) What are the existing agreements for the management of fishery area access (PAAP) in KNPPS

________________

DAILY HABITS IN SEARCHING AND MANAGING SEA MARKETS

Here are some statements about the habits of finding and managing seafood. Please feel free to provide an answer that suits your habits and beliefs.

(8) Other people on this island, who set an example for you to fish in accordance with the agreement are:

[] Community Leader [] Rookie [] Pelele [] Fellow fisherman [] Religion [] Children / Brothers [] None [] Do not know [] TN officer [] Others (specify) ________________

(9) Other people on this island who require you to fish in accordance with the agreement are:

[] Community Leader [] Rookie [] Pelele [] Fellow fisherman [] Religion [] Children / Brothers [] None [] Do not know [] TN officer [] Others (specify) ________________

For the statement below, please state your answer, with "Yes", "No", or 'Can not remember'

(10) ÿÿ9 In the last 6 months, you talked to your fellow fishermen about: ÿÿ1

(A) Benefits derived from PAAP locations

[] Yes [] No [] Do not remember

(B) Obedience to the agreement applicable at the PAAP location

[] Yes [] No [] Do not remember

(C) Appliances are allowed to be used in PAAP locations

[] Yes [] No [] Do not remember

(D) Good fish size captured in PAAP location

[] Yes [] No [] Do not remember

(E) Types of fish managed / managed at PAAP locations

[] Yes [] No [] Do not remember

Here, please tell me whether 'easy,' rather easy ',' hesitant ',' rather difficult ', difficult' to do things yourself in this statement.

(11) For you,

(A) Not looking for fish in the core zone of Kepulauan Seribu National Park

[] Easy [] Somewhat easy [] Hesitant [] Somewhat difficult [] Difficult

(B) Obey the PAAP agreement

[] Easy [] Somewhat easy [] Hesitant [] Somewhat difficult [] Difficult

(C) Engage in proceedings and discussions for PAAP agreements

[] Easy [] Somewhat easy [] Hesitant [] Somewhat difficult [] Difficult

(D) Reporting the catch

[] Easy [] Somewhat easy [] Hesitant [] Somewhat difficult [] Difficult

(E) Reports a breach of agreement at the PAAP location

[] Easy [] Somewhat easy [] Hesitant [] Somewhat difficult [] Difficult

(F) Invite fellow fishermen to obey the PAAP agreement

[] Easy [] Somewhat easy [] Hesitant [] Somewhat difficult [] Difficult

Here, please tell me whether 'Agreed', 'Disagree', 'Do not know' to the statement below

(12) For you to obey the PAAP agreement

(A) Make the sea awake

[] Agree [] Disagree [] Do not know

(B) Then the catch is getting more, the income increases

[] Agree [] Disagree [] Do not know

(C) Will benefit fishing fishermen

[] Agree [] Disagree [] Do not know

(D) It is my responsibility to safeguard for the sake of the next generation / grandchildren

[] Agree [] Disagree [] Do not know

(E) The produce of the sea will be enjoyed until posterity

[] Agree [] Disagree [] Do not know

(13) For you, Obey the PAAP agreement

(A) Will incur additional costs to change fishing gear

[] Agree [] Disagree [] Do not know

(B) Making fishing is not free

[] Agree [] Disagree [] Do not know

(C) Causes reduced revenue

[] Agree [] Disagree [] Do not know

(D) Causing social conflict

[] Agree [] Disagree [] Do not know

(E) There will be no results because outside fishermen will stay in

[] Agree [] Disagree [] Do not know

Here, please tell me whether 'Sure able to do', 'Somewhat sure to do', 'Doubtful', 'Somewhat unsure of being able to do', 'Unsure able to do' the statements below.

(14) You feel,

(A) No fishing in the core zone of TNKpS

[] Sure able to do [] Somewhat sure able to do [] Hesitant [] Somewhat unsure able to do [] Not sure able to do

(B) Catch certain types of fish according to the agreement at the PAAP site

[] Sure able to do [] Somewhat sure able to do [] Hesitant [] Somewhat unsure able to do [] Not sure able to do

(C) Using certain types of fishing gear at the PAAP location

[] Sure able to do [] Somewhat sure able to do [] Hesitant [] Somewhat unsure able to do [] Not sure able to do

(D) Reporting the catch

[] Sure able to do [] Somewhat sure able to do [] Hesitant [] Somewhat unsure able to do [] Not sure able to do

(E) Supervise and report violations at PAAP locations

[] Sure able to do [] Somewhat sure able to do [] Hesitant [] Somewhat unsure able to do [] Not sure able to do

(15) (Enumerator provides maps and explains how to read maps to respondents Enumerators then fill in answers according to the accuracy / inaccuracy of respondents).

The enumerator read this question to the respondent:

From this map, point to / mention all the usual locations you go to fish

(Enumerator: Writing all respondent's answer If not willing to answer write 'No answer')

________________

(A) Based on the location of the fishing that you have mentioned, please select the statement that best describes you now

[] Father does not know the designation rules for this location and does not think to find out [] Father does not know the designation rules for this location but has thought to find out [] Father did not implement the designation rules for this location but in the near future it was thought to do so [] Father has followed the designation rules for this location, but only implemented it for less than 6 months [] Father has followed the rules of designation of this location and has done so in 6 months or more

For the following statement, please choose the one that best describes you right now

(16) For the following statement, please select the one that best describes you now

[] Father did not know the agreement about fishing gear that was allowed in PAAP location and did not think to find out [] Father did not know the agreement of fishing gear is allowed in PAAP location but in the near future thought to find out [] Father already know the permissible fishing gear At PAAP location and in the near future it is possible to do so [] Father has been using the type of fishing gear according to the agreement at PAAP location for less than 6 months [] Father has been using the appropriate type of fishing gear at PAAP location in 6 months or more

(17) For the following statement, please select the one that best describes you now

[] You do not know the agreement about the size of the fish that can be caught in PAAP location and do not think to find out [] You do not know the size of the fish deal that can be arrested in PAAP location but in the near future it is thought to find out [] You already know the size Fish that can be caught at PAAP location and in the near future it is thought to do it [] Father has caught fish with size in accordance with the agreement at PAAP location, and has been doing it less than 6 months [] Father has captured the size of fish according to the agreement at PAAP location and Have done it in 6 months or more

(18) For the following statement, please select the one that best describes you now

[] Father did not participate in PAAP location management and did not think to do it [] Father did not participate in PAAP location management but has thought to find out [] Father has thought to participate in PAAP location management in the near future [] Mr. has participated in the management Location of PAAP, but only implemented it for less than 6 months [] Mr. has participated in PAAP location management and has done it in 6 months or more

(19) For the following statement, please select the one that best describes you now

[] Father was never involved in PAAP site surveillance and did not think of doing it [] Father was never involved in PAAP location surveillance and thought to find out [] Father thinks to be involved in PAAP location surveillance in the near future [] Mr. is already involved in oversight PAAP location, less than 6 months [] Mr. has been involved in PAAP location surveillance, within 6 months or more

TEST OF EFFECTIVENESS OF PAID MEDIA

(20) What activities do you think most effectively convey information about PAAP here?

[] Art Festival [] Fishermen's Meeting [] Religious Activities [] Others (specify) [] Nothing is effective

(21) What activities do you think most effectively convey information about fisheries management rules here?

[] Art Festival [] Fishermen's Meeting [] Religious Activities [] Others (specify) [] Nothing is effective

(22) What media do you think will be most effective in conveying information about PAAP here?

[] Stall banner [] Poster [] Calendar [] Others (specify) [] Nothing is effective

(23) What media do you think are most effective in conveying information about fishery rules here?

[] Stall banner [] Poster [] Calendar [] Others (specify) [] Nothing is effective

*****

Thank you for your willingness to spend some time in this poll.
