# Supplementary material for: Catalyzing sustainable fisheries management through behavior change interventions
Source: Conserv Biol. 2020 Apr 15;34(5):1176–89. doi: 10.1111/cobi.13475 (PMC7540413; doi:10.1111/cobi.13475)
Supplement: Supplementary file 15 — Supplementary Material [file COBI-34-1176-s015.docx]

Preparation (completed by Enumerator / Committee)

No. Questionnaire

________________

Enumerator Code

________________

Day / date of the interview

________________

Name of interview location

[] Latondu Besar Island, Latondu Village [] Rajuni Besar Island, Rajuni Village [] Rajuni Kecil Island, Rajuni Village

Survey Period:

[] Pre-Campaign - Intervention [] Post Campaign - Intervention [] Pre Campaign - Comparison [] Post Campaign - Comparison

KAP SURVEY (KNOWLEDGE, ATTITUDE, PRACTICES)

TAKABONERATE FISHERIES AREA ACCESS MANAGEMENT

Introduction

Good morning / afternoon / afternoon

We intend to conduct research on the Management of Fishery Areas in Latondu Village and Rajuni Village. The purpose of this research is to know the practice of marine resource utilization in Latondu Village and Rajuni Village.

This survey consists of 20 statements, which I will read to you. Please be willing to Mr / Mrs to respond to this statement. This interview can be completed in approximately 40 minutes. Given the importance of this information, we hope that you are willing to answer the questions in this survey. There is no wrong and correct answer. Honesty and openness of Father / Mother is very important in providing this information. Answers Mr / Ms will only be known by us, as research material.

Have you ever been interviewed about this research?

[] Already (end the interview and say thanks) [] Not yet (continue interview)

Will you be interviewed?

[] No (end the interview and say thanks) [] Yes (continue the interview)

SELF INFORMATION

I will read some statements about you. Please kindly give us the answer that best suits you. There is only one answer for each question.

(1) Gender (filled directly by Enumerator)

[] Women [] Men

(2) What is your current age?

[] Under or equal to 17 years [] 18-24 years [] 25-31 years [] 32 - 38 years [] 39 - 45 years [] 46 - 52 years [] Above or equal to 53 years old

(3) Mention your last level of education

[] Never graduated [] Did not finish elementary school [] Graduated from elementary school / equivalent [] Junior High or equivalent [] High School graduate / equal [] Others (specify) ________________

(4) What is your main job?

[] Fishermen full-time (go to AG) [] Part-time fishermen (go to AG) [] Fishers / ponggawa (continued No. 5) [] Civil servants (continued No. 5) Continue to No.5) [] Not working anyway (go to No. 5) [] Others (specify) ________________

The type of fish you most want to catch

________________

The most commonly caught type of fish (one species)

________________

The type of fishing gear used

________________

Fishing time

________________

Location of Arrest

________________

(B) The average costs incurred for fishing each time to go to sea are:

[] Rp 100.000 [] Rp 100.000- Rp 250.000 [] Rp 250.001- Rp 500.000 [] Rp 500.001- Rp 1,000,000 [] Above Rp. 1,000,000

(C) During the west season (December - May), how many times do you usually go out to sea to fish in a month?

[] Less or equal to 4 times in a month [] 5-20 times per month [] 21 - 36 times per month [] more than 36 times per month [] uncertain

(D) During the east season (June - November), how many times do you usually go out to sea to fish in a month?

[] Less or equal to 4 times in a month [] 5-20 times per month [] 21 - 36 times per month [] more than 36 times per month [] uncertain

(E) Your source of capital for fishing, usually obtained from:

[] Own / family [] skipper / ponggawa [] Others (specify) ________________

(F) In this 1 month, how did your catch compare to the same month last year?

[] Same [] More [] More and more bigger [] Less [] Uncertain [] Do not remember / do not know

(G) In this 1 month, to get the same amount of fish as last year, your fishing distance:

[] Same course [] Closer than last year [] Farther than last year [] Not sure [] Do not remember

(5) What is the number of your family members in one house (including yourself)

[] 1 person (just yourself) [] 2 people [] 3 people [] 4 people [] equal or more than 5 people

(6) Average monthly expenditure of your family is:

[] Rp 500,000 [] Rp 500.001 - Rp 1,000,000 [] Rp 1,000.001 - Rp 2,000,000 [] Rp 2,000.001 - Rp 3,000,000 [] above Rp 3,000,000

FISHERY MANAGEMENT

Here are two questions about fisheries management. Please feel free to give the best answer according to your opinion.

(7) In your own words, please explain what is meant by Area Fisheries Access Management. (If the respondent answers "Not Know", write "Do not Know")

________________

(8) State all existing rules for management of fishery area access

________________

DAILY HABITS IN SEARCHING AND MANAGING SEA MARKETS

Here are some statements about the habits of finding and managing seafood. Please kindly give your answer in accordance with the habits and beliefs of Mr / Ms.

(9) Other people in this village, who set an example for me to find fish according to the rules are:

[] Head of village [] Head of hamlet / neighborhood [] Chairman of fishing group [] Skipper / Ponggawa [] Fellow fisherman [] Imam Masjid [] Family [] None [] Do not know [] Others (specify) ________________

(10) Other people in this village who require me to find fish according to the rules is

[] Head of village [] Head of hamlet / neighborhood [] Chairman of fishing group [] Skipper / Ponggawa [] Fellow fisherman [] Imam Masjid [] Family [] None [] Do not know [] Others (specify) ________________

For the statement below, please state your answer, with "Yes", "No", or 'Can not remember'

(11) In the last 6 months, I talked with fellow fishermen about:

(A) benefits gained from the management of the fishery area access

[] Yes [] No [] Do not remember

(B) compliance with applicable rules within the territory of the fishery access area

[] Yes [] No [] Do not remember

(C) ways of monitoring and reporting violations of rules in the area of ​​access of the fishing area

[] Yes [] No [] Do not remember

Here, please tell me whether 'easy,' rather easy ',' hesitant ',' rather difficult ', difficult' to do things yourself in this statement.

(12) For me,

(A) does not fish in the no-take / core zone

[] Easy [] Hesitant [] Difficult

(B) complies with the rules of access management of the fishing area

[] Easy [] Hesitant [] Difficult

(C) engage in processes and discussions for the management rules of the fishery area access

[] Easy [] Hesitant [] Difficult

(D) report the catch

[] Easy [] Hesitant [] Difficult

(E) report a violation of the rules in the area of ​​access of the fishery area

[] Easy [] Hesitant [] Difficult

(F) invites fellow fishermen to comply with the management rules of fisheries area access

[] Easy [] Hesitant [] Difficult

Here, please Mr / Mrs declare whether 'Agreed', 'Disagree', 'Do not know' to the statement below

(13) For me, obey the rules of fishery area access management

(A) is a form of responsibility as a fisherman in the region

[] Agree [] Disagree [] Do not know

(B) ensuring the continuation of my family's life in the future

[] Agree [] Disagree [] Do not know

(C) maintaining the availability of fish and other marine resources for a long time

[] Agree [] Disagree [] Do not know

(D) preserves the traditions of life as fishermen from generation to generation

[] Agree [] Disagree [] Do not know

(14) To me, adhere to the rules of access management of the fishing area

(A) will incur additional costs to replace fishing gear

[] Agree [] Disagree [] Do not know

(B) can not be done because there is no firmness against rule violators

[] Agree [] Disagree [] Do not know

(C) can not be executed because there is no visible border for the location of the fishery area access

[] Agree [] Disagree [] Do not know

(D) makes the time to go to sea longer due to reporting the catch

[] Agree [] Disagree [] Do not know

Here's what you want to do, 'Somewhat sure to be able to do', 'Doubtful', 'Somewhat unsure of being able to do', 'Unsure able to do' the following statements.

(15) I feel,

(A) does not catch fish in the no-take area

[] Sure able to do [] Hesitant [] Not sure able to do

(B) catch fish according to the rules in the area of ​​fishery access area

[] Sure able to do [] Hesitant [] Not sure able to do

(C) using the type of fishing gear permitted in the area of ​​fishery access area

[] Sure able to do [] Hesitant [] Not sure able to do

(D) reporting the catch

[] Sure able to do [] Hesitant [] Not sure able to do

(E) supervise and report violations in the area of ​​access of the fishing area

[] Sure able to do [] Hesitant [] Not sure able to do

(16) (Enumerator provides maps and explains how to read maps to respondents Enumerators then fill in answers according to the accuracy / inaccuracy of respondents).

The enumerator read this question to the respondent:

From this map, point to / mention all the locations you usually go looking for fish

(Enumerator: Writing all respondent's answer If not willing to answer write 'No answer')

________________

(A) Based on the location of the above mentioned fishing / fowl, please Mr / Mrs choose the statement that best describes you right now

[] I do not know the designation rules for this area and do not think to find out [] I do not know the designation rules for this area but have been thinking about finding out [] I am not implementing the designation rules for this area but in the near future I think to do it [] I have followed the designation rules for this area, but only implemented it for less than 6 months [] I have followed the rules of the designation of this area and have done so in 6 months or more

For the following statement, please choose the one that best describes you right now

(17) For the following statement, please choose the one that best describes you right now

[] I do not know the rules about fishing gear allowed in the area of ​​access area and do not think to find out [] I do not know the rules of fishing gear are allowed in the area of ​​access area but in the near future thought to find out [] I already know fishing gear Which is allowed in the area of ​​access area and in the near future it is thought to do it [] I have been using the type of fishing gear according to the rules in the area of ​​access area, for less than 6 months [] I have used the type of fishing gear that match the rules of area access area, 6 months or more

(18) For the following statement, please choose the one that best describes you right now

[] I do not know the rules about the size of the catch in the area of ​​access area and do not think to find out [] I do not know the catch size rules in the area of ​​access area but in the near future it is thought to find out [] I already know the size of the catch allowed in the region Access area and in the near future think to do it [] I have caught fish with the size of fish catch according to the rules in the area of ​​access area, and have been doing it for less than 6 months [] I have captured the size of the fish according to the rules of area access area and have done it in 6 Months or more

(19) For the following statement, please choose the one that best describes you right now

[] I did not participate in the management of area access areas and did not think to do it [] I did not participate in the management of area access areas but have thought to find out [] I have thought about participating in the management of area access areas in the near future [] I have Participate in the management of area access areas, but only implement them for less than 6 months [] I have participated in the management of area access areas and have done so in 6 months or more

(20) For the following statement, please choose the one that best describes you right now

[] I have never been involved in surveillance of the territory of the fisheries access area and have not thought of doing it [] I have never been involved in surveillance of area access areas and thought to find out [] I was thinking of being involved in the monitoring of area access areas, in the near future [] I Has been involved in the supervision of area access areas, less than 6 months [] I have been involved in the supervision of the area of ​​access to the fishing area, within 6 months or more

*****

Thank you for your willingness to take the time to answer this survey.
