# Supplementary material for: Catalyzing sustainable fisheries management through behavior change interventions
Source: Conserv Biol. 2020 Apr 15;34(5):1176–89. doi: 10.1111/cobi.13475 (PMC7540413; doi:10.1111/cobi.13475)
Supplement: Supplementary file 16 — Supplementary Material [file COBI-34-1176-s016.docx]

Preparation (completed by Enumerator / Committee)

No. Questionnaire

________________

Enumerator Code

________________

Day / date of the interview

________________

Name of interview location

[] Liya Togo Village [] Liya Bahari Beautiful Village [] Liya Mawi Village [] Liya Onemelangka Village [] Liya Wisata Wisata Kollo

Survey Period:

[] Pre-Campaign - Intervention [] Post Campaign - Intervention [] Pre Campaign - Comparison [] Post Campaign - Comparison

SURVEY KAP KADIE LIYA PASKA KAMPANYE

Introduction

Good morning / afternoon / afternoon

We are from pride rare Indonesia campaign team and DKP will conduct fishery research. The purpose of this research is to know how fishery management by fisherman in kyae liya region.

This survey consists of 24 statements, which I will read to you. Please be willing to Mr / Mrs to respond to this statement. This interview can be completed in approximately 30 minutes. Given the importance of this information, we hope that you are willing to answer the questions in this survey. There is no wrong and correct answer. Honesty and openness of Father / Mother is very important in providing this information. Answers Mr / Mrs will only be known by us, as researchers.

Have you ever been interviewed before about the campaign?

[] Already (end the interview and say thanks) [] Not yet (continue interview)

Will you be interviewed?

[] No (end the interview and say thanks) [] Yes (continue the interview)

SELF INFORMATION

I will read some statements about you. Please kindly give us the answer that best suits you. There is only one answer for each question.

(1) Gender (filled directly by Enumerator)

[] Women [] Men

(2) What is your current age?

[] Under or equal to 17 years [] 18-24 years [] 25-31 years [] 32 - 38 years [] 39 - 45 years [] 46 - 52 years [] Above or equal to 53 years old

(3) Mention your last level of education

[] Never graduated [] Did not finish elementary school [] Graduated from elementary school / equivalent [] Junior High or equivalent [] High School graduate / equal [] Others (specify) ________________

(4) What is your main job?

[] Fishermen full-time (go to AF) [] Part-time fishermen (go to AF) [] Civil servants (continued to No. 5) [] Not working anyway (go to No. 5) [] Others (specify) ________________

The most commonly caught type of fish (one species)

________________

The type of fishing gear used

________________

Fishing time

________________

(A) The average costs incurred for fishing each time to go to sea are:

[] Under Rp 500,000 [] Rp 500,000 - Rp 1,000,000 [] Rp 1.000.000- Rp 2,000,000 [] Above Rp 2,000,000

(B) Within a month, you usually do sea activities to find as many fish

[] Less than 5 times in a month [] 5-10 times per month [] 10 - 20 times per month [] more than 20 times per month [] uncertain

(C) Your source of capital for fishing, usually obtained from:

[] Self Money [] Collector [] cooperative [] borrow friend [] Others (specify) ________________

(D) In ​​1 month, how did your catch compare to the same month last year?

[] Same [] More [] More and more bigger [] Less [] Uncertain [] Do not remember / do not know

(E) In this 1 month, to get the same amount of fish as last year, your fishing distance:

[] Same course [] Closer than last year [] Farther than last year [] Not sure [] Do not remember

(5) What is the number of your family members in one house (including yourself)

[] 1 person (just yourself) [] 2 people [] 3 people [] 4 people [] equal or more than 5 people

(6) Average monthly expenditure of your family is:

[] Under Rp 1,000,000 [] Rp 1,000,000- Rp 2,000,000 [] Rp 2,000,000 - Rp 3,000,000 [] above Rp 3,000,000

FISHERY MANAGEMENT

Here are two questions about fisheries management. Please feel free to give the best answer according to your opinion.

(7) In your own words, please explain what is meant by Area Fisheries Access Management. (If the respondent answers "Not Know", write "Do not Know")

________________

(8) State all existing rules for management of fishery area access

________________

DAILY HABITS IN SEARCHING AND MANAGING SEA MARKETS

Here are some statements about the habits of finding and managing seafood. Please kindly give your answer in accordance with the habits and beliefs of Mr / Ms.

(9) Other people in this village, who set an example for me to find fish according to the rules are:

[] Chairman of the group [] Chairman of the fishing group [] Chairman of the cooperative [] Collector [] Fellow fisherman [] Chairman of the Mosque / Church [] Wife / husband [] Children [] Chairman of Customs [] Community / Religious Leader [] Others (specify) ________________

(10) Other people in this village who require me to find fish according to the rules is

[] Chairman of the group [] Chairman of the fishing group [] Chairman of the cooperative [] Collector [] Fellow fisherman [] Chairman of the Mosque / Church [] Wife / husband [] Children [] Chairman of Customs [] Community / Religious Leader [] Others (specify) ________________

For the statement below, please state your answer, with "Yes", "No", or 'Can not remember'

(11) In the last 6 months, I talked with fellow fishermen about:

(A) benefits gained from the management of the fishery area access

[] Yes [] No [] Do not remember

(B) compliance with applicable rules within the territory of the fishery access area

[] Yes [] No [] Do not remember

(C) ways of monitoring and reporting violations of rules in the area of ​​access of the fishing area

[] Yes [] No [] Do not remember

Here, please tell me whether 'easy,' rather easy ',' hesitant ',' rather difficult ', difficult' to do things yourself in this statement.

(12) For me,

(A) does not look for fish in the DPL area

[] Easy [] Somewhat easy [] Hesitant [] Somewhat difficult [] Difficult

(B) complies with the rules of access management of the fishing area

[] Easy [] Somewhat easy [] Hesitant [] Somewhat difficult [] Difficult

(C) engage in processes and discussions for the management rules of the fishery area access

[] Easy [] Somewhat easy [] Hesitant [] Somewhat difficult [] Difficult

(D) report the catch

[] Easy [] Somewhat easy [] Hesitant [] Somewhat difficult [] Difficult

(E) report a violation of the rules in the area of ​​access of the fishery area

[] Easy [] Somewhat easy [] Hesitant [] Somewhat difficult [] Difficult

(F) invites fellow fishermen to comply with the management rules of fisheries area access

[] Easy [] Somewhat easy [] Hesitant [] Somewhat difficult [] Difficult

Here, please Mr / Mrs declare whether 'Agreed', 'Disagree', 'Do not know' to the statement below

(13) For me, obey the rules of fishery area access management

(A) is a form of responsibility as a fisherman in the region

[] Agree [] Disagree [] Do not know

(B) ensuring the continuation of my family's life in the future

[] Agree [] Disagree [] Do not know

(C) maintaining the availability of fish and other marine resources for a long time

[] Agree [] Disagree [] Do not know

(D) preserves the traditions of life as fishermen from generation to generation

[] Agree [] Disagree [] Do not know

(14) To me, adhere to the rules of access management of the fishing area

(A) will incur additional costs to replace fishing gear

[] Agree [] Disagree [] Do not know

(B) can not be done because there is no firmness against rule violators

[] Agree [] Disagree [] Do not know

(C) can not be executed because there is no visible border for the location of the fishery area access

[] Agree [] Disagree [] Do not know

(D) makes the time to go to sea longer due to reporting the catch

[] Agree [] Disagree [] Do not know

Here's what you want to do, 'Somewhat sure to be able to do', 'Doubtful', 'Somewhat unsure of being able to do', 'Unsure able to do' the following statements.

(15) According to you,

(A) does not catch fish in the DPL area

[] Sure able to do [] Somewhat sure able to do [] Hesitant [] Somewhat unsure able to do [] Not sure able to do

(B) catch fish according to the rules in the area of ​​fishery access area

[] Sure able to do [] Somewhat sure able to do [] Hesitant [] Somewhat unsure able to do [] Not sure able to do

(C) using the type of fishing gear permitted in the area of ​​fishery access area

[] Sure able to do [] Somewhat sure able to do [] Hesitant [] Somewhat unsure able to do [] Not sure able to do

(D) reporting the catch

[] Sure able to do [] Somewhat sure able to do [] Hesitant [] Somewhat unsure able to do [] Not sure able to do

(E) supervise and report violations in the area of ​​access of the fishing area

[] Sure able to do [] Somewhat sure able to do [] Hesitant [] Somewhat unsure able to do [] Not sure able to do

(16) (Enumerator provides maps and explains how to read maps to respondents Enumerators then fill in answers according to the accuracy / inaccuracy of respondents).

The enumerator read this question to the respondent:

From this map, point to all the locations where you usually go fishing

(Enumerator: Writing all respondent's answer If not willing to answer write 'No answer')

________________

(A) Based on the location of the above mentioned fishing / mothership, please Mr. / Mrs. choose the statement that best describes you the current father

[] I do not know the rules of fisheries management for this area and do not think to find out [] I do not know the rules of fisheries management for this region but have thought to find out [] I am not implementing fisheries management rules for this area but in the near future Thought to do it [] I have followed the rules of fisheries management for this area, but just implemented it for less than 6 months [] I have followed the rules of fisheries management of this area and have done it in 6 months or more

For the following statement, please choose the one that best describes you right now

(17) For the following statement, please choose the one that best describes you right now

[] I do not know the rules about fishing gear allowed in the area of ​​access area and do not think to find out [] I do not know the rules of fishing gear are allowed in the area of ​​access area but in the near future thought to find out [] I already know fishing gear Which is allowed in the area of ​​access area and in the near future it is thought to do it [] I have been using the type of fishing gear according to the rules in the area of ​​access area, for less than 6 months [] I have used the type of fishing gear that match the rules of area access area, 6 months or more

(18) For the following statement, please choose the one that best describes you right now

[] I do not know the rules about the size of fish that can be caught in the area of ​​access area and do not think to find out [] I do not know the fish size rules that can be caught in the area of ​​access area but in the near future thought to find out [] I already know The size to be caught is allowed in the area of ​​access area and in the near future it is thought to do it [] I have caught fish with the size of the fish according to the rules in the area of ​​access area, and have done it less than 6 months [] I have caught with the size of the fish according to the rules Area access area and have been doing it in 6 months or more

(19) For the following statement, please choose the one that best describes you right now

[] I did not participate in the management of area access areas and did not think to do it [] I did not participate in the management of area access areas but had thought to find out [] I have been thinking about participating in the management of area access areas in the near future [] I have participated in the management of the area access area, but only implemented it for less than 6 months [] I have participated in the management of the area access area and have done so in 6 months or more

(20) For the following statement, please choose the one that best describes you right now

[] I have never been involved in surveillance of the territory of the fisheries access area and have not thought of doing it [] I have never been involved in surveillance of area access areas and thought to find out [] I was thinking of being involved in the monitoring of area access areas, in the near future [] I Has been involved in the supervision of area access areas, less than 6 months [] I have been involved in the supervision of the area of ​​access to the fishing area, within 6 months or more

MEDIA EFFECTIVENESS AND CAMPAIGN ACTIVITIES

(21) What activities do you think most effectively convey information about PAAP here?

[] Art Festival [] Fisherman's Meeting [] Activities of the Competition [] Nothing Effective [] Others (specify) ________________

(22) What activities do you think most effectively convey information about fisheries management rules here?

[] Art Festival [] Fisherman's Meeting [] Activities of the Competition [] Nothing Effective [] Others (specify) ________________

(23) What media do you think are most effective in conveying information about PAAP here?

[] Banners / Posters [] Poster [] Calendar [] Nothing effective [] Others (specify) ________________

(24) What media do you think are most effective in conveying information about fishery rules here?

[] Banners / Posters [] Poster [] Calendar [] Nothing effective [] Others (specify) ________________

*****

Thank you for your willingness to take the time to answer this survey.
