# Supplementary material for: Catalyzing sustainable fisheries management through behavior change interventions
Source: Conserv Biol. 2020 Apr 15;34(5):1176–89. doi: 10.1111/cobi.13475 (PMC7540413; doi:10.1111/cobi.13475)
Supplement: Supplementary file 18 — Supplementary Material [file COBI-34-1176-s018.docx]

**fassetsRARE FISH FOREVER PROGRAM**

**BASELINE DATA COLLECTION AND REPORTING**

**HOUSEHOLD SURVEY INTERVIEW SCHEDULE**

**INTRODUCTION**

***Maayong Adlaw, ako si ____. Nagatrabaho ako sa Capitol University Extension Office (CUREXO), nga gi-commissioned sa RARE Foundationpara sa usa ka pagtuon nga nagasusi sa panginabuhi sa mga mangingisda, kahimtang sa panagat ug pnginabuhian, kahimtang sa inyong kinaiyanhong bahandi sa dagat, pamaagi ug strateheya sa local nga pangagamhanan aron maprotektahan ug mabantayan ang kadagatan ug mangingisda sa ilang komunidad. Ania kami karon aron mangayo ug inpormasyon sa inyong katilingbanon (social) ug ekonomikanhong kahimtang alang sa kalamboan sa inyong kadagatan ug komunidad. Ikaw usa sa napili (nabunotan) alang niini nga pagtuon.***

***Kini nga interview molungtad sa usa ka oras ug kapin. Palihug tubaga tanang pangutana base sa imong katakos, kahibalo, ug abilidad. Among ipasalig kanimo nga ang tanang impormasyon nga imong ihatag kanamo among ampingan nga walay laing masayod ug nga gamiton lamang alang sa katuyuan niini nga pagtuon.***

***Kung aduna kay mga pangutana o problema mahitungod niini nga pagtuon/survey, mahimo ka makigkita o tawagan si Dr. Imelda G. Pagtolun-an sa Capitol University Extension Office (CUREXO) niining mosunod nga mga contact* number 088-858-3116 local 3022 and 08822 723228.**

Good Day. My name is __________I am working with the Capitol University Research and Extension Office (CUREXO) commission by the RARE Foundation to undertake this baseline study of the current condition of fisherfolks, marine resources and management, strategies and processes of local government in protecting marine resources and fisherfolk in their local and municipal fishing grounds.As part of this undertaking, we are here to gather data/information on your current socio-economic conditionsin relation to marine resource development. You have been randomly selected to participate in this study.

This interview will probably take about an hour. Please answer every question asked of you to the best of your knowledge and ability. Rest assured that all information you will share with us will be treated with utmost confidentiality and will be used only for the purpose of this study.

If you have any questions or problems pertaining to this study, you may see or call Dr. Imelda G. Pagtolun-anof the Capitol University Research and Extension Office (CUREXO) at the following numbers: 088-858-3116 local 3022; and 08822 723228.

| **Aduna bay sakop sa inyong panimalay nga nagapangisda o ang pnginabuhian anaa sa dagat o nagsideline sa pagpanagat?** (Do you or any member of your household rely either entirely or in part on fishing or on productive activities or employment related to fishery for your household’s livelihood ie, to provide for the family’s needs)? | 1. Yes 2. No. End Interview (Replace Sample) |
| --- | --- |

**Oral Consent Form**

*SHOW AND READ ORAL CONSENT FORM*

**(To be read to the respondent prior to asking questions.)**

**Akong subli-on nga ang tumong niini nga pagtu-on mao ang pagpakisayod kabahin sa inyong kahimtang ug kasinatian karon isip usa ka mangingisda dinhi sa inyong dapit alang sa kalamboan ug kaayuhan sa inyong pamilya ug sa inyong dapit. Ang mga impormasyon nga makuha gamiton lamang gayud niini nga pagtu-on ug dili mogawas ang imong ngalan sa resulta unya sa pagtu-on. Ang imong partisipasyon sa pagtubag sa mga pangutana boluntaryo. Among isubli nga ang imong mga tubag walay laing masayod ug palihug sa pag-pirma niining oral consent form.**

**Respondent’s Consent**

| **Ang tanang aspeto niini nga pagtuon gipasabot nako pag-ayo sa respondent. Mi-uyon siya sa pag-apil ug pagtubag sa akong mga pangutana.**  (I have fully explained all aspect of the study to the respondent. He/she consented to answer the interview questions.)  **_____________________________________**  **Interviewer’s Signature, over printed name** | ***Nasabtan ko pag-ayo and katuyuan niini nga pagtuon. Gihatag nako ang akog pag-uyon sa pag-salmot ug pagtubag niini nga pagtuon***. (I have fully understood the purpose of the study. I have given my consent to participate in the survey.)  **_______________________________________**  **Responent’s Signature over printed name** |
| --- | --- |

| **SECTION A: IDENTIFICATION AND BACKGROUND INFORMATION** | | |
| --- | --- | --- |
| Name of Respondent |  | Mobile No: |
| Name of interviewer |  | |
| Call Record | | |
| \| **Visit** \| **Date** \| **Time Start** \| **Time End** \| **Remarks** \| \| --- \| --- \| --- \| --- \| --- \| \| 1 \|  \|  \|  \|  \| \| 2 \|  \|  \|  \|  \| \| 3 \|  \|  \|  \|  \| | | |

**Location and Identification**

| Sitio/Purok |  | | | |
| --- | --- | --- | --- | --- |
| Barangay |  | | | |
| Municipality |  | | | |
| Province |  | | | |
| Region |  | | | |
| Stratum | □ | Program Area | □ | Control |
| Type of respondent (pls. check) | □ | Original | □ | Replacement |
| Reason for replacement (if replaced) |  | | | |

**Editing**

| **Field Edited By:** | **Office Edited By:** |
| --- | --- |
|  |  |

**SECTION B: HOUSEHOLD INFORMATION AND CHARACTERISTICS**

| B1. | **Lakip ang imong kaugalingon, pila ka tawo ang permaninting nagpuyo niini nga panimalay karon? Ilan ang permanenteng nakatira sa bahay na ito?** (Including yourself, how many persons are permanently residing in this household?) | | **_____** | **persons** | |
| --- | --- | --- | --- | --- | --- |
| B2 | **Pila na ka tuig nga nagpuyo ang imong panimalay/pamilya niining inyong dapit?**  **Gaano na kayo katagal nakatira dito?** How long has your household been residents in this community?) | | **_____** | **years** | |
| B3 | **Sugod sa pangulo sa panimalay, palihug nganli ang tanang myembro sa inyong panimaly nga nagpuyo dinhi sa pagkakaron. Maari po bang pangalanan ang lahat ng nakatira rito. Simulan po natin sa puno ng sambahayan.**(Starting with the household headplease name all the members presently residing in this household.)   \| B3a. Name \| \| B3b. Relation to HH Head \| Age \| Sex \| Civil Status (10 yrs. Old & over \| Education (grade/level completed) (3 yrs old & over \| Currently Attending School (asked of 6 to 17 yrs old) \| Main (Primary) Occupation  (FOR 10 YRS. OLD & OVER( \| Sideline/  Secondary Occupation \| Did any work for at least one hour in the past week? (check) \| Annual Income from all sources \| Amount of income contributed to HH income \| \| --- \| --- \| --- \| --- \| --- \| --- \| --- \| --- \| --- \| --- \| --- \| --- \| --- \| \| **01** \|  \|  \|  \|  \|  \|  \|  \|  \|  \|  \|  \|  \| \| **02** \|  \|  \|  \|  \|  \|  \|  \|  \|  \|  \|  \|  \| \| **03** \|  \|  \|  \|  \|  \|  \|  \|  \|  \|  \|  \|  \| \| **04** \|  \|  \|  \|  \|  \|  \|  \|  \|  \|  \|  \|  \| \| **05** \|  \|  \|  \|  \|  \|  \|  \|  \|  \|  \|  \|  \| \| **06** \|  \|  \|  \|  \|  \|  \|  \|  \|  \|  \|  \|  \| \| **07** \|  \|  \|  \|  \|  \|  \|  \|  \|  \|  \|  \|  \| \| **08** \|  \|  \|  \|  \|  \|  \|  \|  \|  \|  \|  \|  \|  \| ***CODES—CODES—CODES--CODES*** \| \| \| \| \| \| \| \| \| --- \| --- \| --- \| --- \| --- \| --- \| --- \| --- \| \| **Relationship to HH head** \| \| **SEX** \| **CIVIL STATUS** \| **EDUCATION** \| \| **MAIN OCCUPATION** \| **SIDELINE** \| \| 1. *Household head* 2. *Spouse of household head* 3. *Daughter* 4. *Son* 5. *Niece* 6. *Nephew* \| 1. *In-law* 2. *Sibling* 3. *Father* 4. *Mother* 5. *Others (specifY): ____________*   *___________* \| 1. *Male* 2. *Female* \| 1. *Single* 2. *Married (to include common law marriages)* 3. *Widowed* 4. *Separated* 5. *Others, specify________* \| 1. *No education* \| 1. *Vocational course* \| 1. *Employed (****Indicate actual work or position in an institution or office)*** \| 1. *Employed (****Indicate actual work or position in an institution or office)*** \| \| 1. *Elementary but did not graduate* \| 1. *College level but not graduate* \| 1. *Hired worker in non farming related activities (****specify type of work)*** \| 1. *Hired worker in non farming related activities (****specify type of work)*** \| \| 1. *Elementary graduate* \| 1. *College Graduate* \| 1. *Self Employed / Business / Trade (****specify type of business)*** \| 1. *Self Employed / Business / Trade (****specify type of business)*** \| \| 1. *Highschool but did not graduate* \| 1. *Others (specify) _____________* \| 1. *Crop Farming (* ***write types of crops, total hectares of farm and whether own, lease, or tenant)*** \| 1. *Crop Farming (* ***write types of crops, total hectares of farm and whether own, lease, or tenant)*** \| \| 1. *Highschool graduate* \| 1. *Livestock Raising (****heads of animals)*** \| 1. *Livestock Raising (****heads of animals)*** \| \| 1. *Fisherfolk (****type of catch)*** \| 1. *Fisherfolk (****type of catch)*** \| | | | | |
| B4 | **Nahisakop ka ba sa usa ka tribo o grupo sa tribal community? Kabilang po ba kayo sa anumang katutubong grupo o indigenous na pangkat?** (Do you belong to an indigenous group?) | 1 Yes  2 No (GO TO C1) | | | |
| B5 | **Unsa imong tribu? Ano pong katutubong grupo ang inyong kinabibilangan?** (What is this indigenous group?) | ***Luzon***   - 1. Bontoc   2. Gaddangs   3. Ibaloi   4. Ifugao   5. Ilongots   6. Isneg   7. Kalinga   8. Kankana-ey   9. Mangyan   10. Negritos   11. Tinguian | ***Visayas and Mindanao***   - 1. Ati   2. Badjao   3. Bagobo   4. Bila-an   5. Bukidnon   6. Higaonon   7. Maguindanao   8. Mamanua   9. Mandaya   10. Manobo   11. Mansaka | | - 1. Maranao   2. Samal   3. Subanon   4. Tausug   5. Tiboli   6. Tiruray   7. Agta   8. Kamayo   9. Dumagat   10. Others, (specify)   _________________________ |

**SECTION C: FISHING AND AQUACULTURE PRODUCTION AND OTHER FISHING RELATED ACTIVITIES**

| **C1** | **Palihug nganli ang miembro sa panimalay nga nagapangisda o adunay aquacultureproduction o ang panginabuhian dunay kalabutan sa dagat o pangisda? Pila ka miembro ___________?**   \| C1a. Name \| C1b. Gender \| C1c. Full-time or part-time fisher (or aquaculture production) \| C1d. Lugar kung asa nangisda (Location of fishing area/activities (Where do you usually fish?) \| C1e. How often do you fish? Number of hours per week \| C1f. Type of fishing gear usually used (describe each gear and write local names) \| \| --- \| --- \| --- \| --- \| --- \| --- \| \|  \|  \|  \|  \|  \|  \| \|  \|  \|  \|  \|  \|  \| \|  \|  \|  \|  \|  \|  \| \|  \|  \|  \|  \|  \|  \| \|  \|  \|  \|  \|  \|  \| \|  \|  \|  \|  \|  \|  \| \|  \|  \|  \|  \|  \|  \| \|  \|  \|  \|  \|  \|  \| \|  \|  \|  \|  \|  \|  \| \|  \|  \|  \|  \|  \|  \| \|  \|  \|  \|  \|  \|  \| \|  \|  \|  \|  \|  \|  \| \|  \|  \|  \|  \|  \|  \| \|  \|  \|  \|  \|  \|  \| \|  \|  \|  \|  \|  \|  \| \|  \|  \|  \|  \|  \|  \| \|  \|  \|  \|  \|  \|  \| \|  \|  \|  \|  \|  \|  \| \|  \|  \|  \|  \|  \|  \| \|  \|  \|  \|  \|  \|  \| \|  \|  \|  \|  \|  \|  \| \| Copy names from HH roster \| 1- male  2-female \| 1- full-time fisher, most of time is spent in fishing  2- part-time fisher – most of time is spent in another work or another activities \| 1- within municipal waters or fishing ground  2 – outside municipal waters or fishing grounds  3- others \| Write actual numbers and hours per week \| 1 – nets (include mesh size, length, height/depth)  2 – hook & lines (include hook size, number of hooks, bait used, target fish)  3 –bubo/fish pot/crab pot (include dimension, bait used, target fish)  4- spear fishing/pana (ask if mano-mano or using compressor)  5 – Others (describe the gear) \| |
| --- | --- | --- | --- | --- | --- | --- | --- | --- | --- | --- | --- | --- | --- | --- | --- | --- | --- | --- | --- | --- | --- | --- | --- | --- | --- | --- | --- | --- | --- | --- | --- | --- | --- | --- | --- | --- | --- | --- | --- | --- | --- | --- | --- | --- | --- | --- | --- | --- | --- | --- | --- | --- | --- | --- | --- | --- | --- | --- | --- | --- | --- | --- | --- | --- | --- | --- | --- | --- | --- | --- | --- | --- | --- | --- | --- | --- | --- | --- | --- | --- | --- | --- | --- | --- | --- | --- | --- | --- | --- | --- | --- | --- | --- | --- | --- | --- | --- | --- | --- | --- | --- | --- | --- | --- | --- | --- | --- | --- | --- | --- | --- | --- | --- | --- | --- | --- | --- | --- | --- | --- | --- | --- | --- | --- | --- | --- | --- | --- | --- | --- | --- | --- | --- | --- | --- | --- | --- | --- | --- |

| **C1a.** Please indicate the species of fish that each member caught, volume of fish caught and price per kilo per month**.** | | | | | | | | | | | | | | | | | | | | | | | | | | | | |
| --- | --- | --- | --- | --- | --- | --- | --- | --- | --- | --- | --- | --- | --- | --- | --- | --- | --- | --- | --- | --- | --- | --- | --- | --- | --- | --- | --- | --- |
|  | **November 2016** | | | | | | **December 2016** | | | | | | **January 2017** | | | | | | | | **February 2017** | | | | | | |  |
| **Classification** | **1-Lean 2-Peak** | | | | | | **1-Lean 2-Peak** | | | | | | **1-Lean 2-Peak** | | | | | | | | **1-Lean 2-Peak** | | | | | | |  |
| **C1aa. Name of member engaged in fishing** | **Species of fish caught** | **Volume of fish caught** | **Farm gate price per kilo** | | | **Market Price per kilo** | **Species of fish caught** | **Volume of fish caught** | | **Farm gate price per kilo** | | **Market Price per kilo** | **Species of fish caught** | **Volume of fish caught** | **Farm gate price per kilo** | | | | **Market Price per kilo** | | **Species of fish caught** | **Volume of fish caught** | **Farm gate price per kilo** | | | | **Market Price per kilo** |  |
|  |  |  |  | | |  |  |  | |  | |  |  |  |  | | | |  | |  |  |  | | | |  |  |
|  |  |  |  | | |  |  |  | |  | |  |  |  |  | | | |  | |  |  |  | | | |  |  |
|  |  |  |  | | |  |  |  | |  | |  |  |  |  | | | |  | |  |  |  | | | |  |  |
|  |  |  |  | | |  |  |  | |  | |  |  |  |  | | | |  | |  |  |  | | | |  |  |
|  |  |  |  | | |  |  |  | |  | |  |  |  |  | | | |  | |  |  |  | | | |  |  |
|  |  |  |  | | |  |  |  | |  | |  |  |  |  | | | |  | |  |  |  | | | |  |  |
|  |  |  |  | | |  |  |  | |  | |  |  |  |  | | | |  | |  |  |  | | | |  |  |
|  |  |  |  | | |  |  |  | |  | |  |  |  |  | | | |  | |  |  |  | | | |  |  |
|  |  |  |  | | |  |  |  | |  | |  |  |  |  | | | |  | |  |  |  | | | |  |  |
|  |  |  |  | | |  |  |  | |  | |  |  |  |  | | | |  | |  |  |  | | | |  |  |
|  |  |  |  | | |  |  |  | |  | |  |  |  |  | | | |  | |  |  |  | | | |  |  |
|  |  |  |  | | |  |  |  | |  | |  |  |  |  | | | |  | |  |  |  | | | |  |  |
|  |  |  |  | | |  |  |  | |  | |  |  |  |  | | | |  | |  |  |  | | | |  |  |
|  |  |  |  | | |  |  |  | |  | |  |  |  |  | | | |  | |  |  |  | | | |  |  |
|  |  |  |  | | |  |  |  | |  | |  |  |  |  | | | |  | |  |  |  | | | |  |  |
|  |  |  |  | | |  |  |  | |  | |  |  |  |  | | | |  | |  |  |  | | | |  |  |
|  |  |  |  | | |  |  |  | |  | |  |  |  |  | | | |  | |  |  |  | | | |  |  |
|  |  |  |  | | |  |  |  | |  | |  |  |  |  | | | |  | |  |  |  | | | |  |  |
| **CODES: Species of fish** | | | | | | | | | | | | | | | | | | | | | | | | | | | | |
| **C1ab.** Total monthly expense in fishing (material input, labor, transport cost, water & electricity, space rental, other expense | 1- grouper (lapu-lapu)  2- parrot fish  3- emperor  4- snapper  5- sweetlip  6- goatfish  7-surgeonfish  8- squid  9-shrimps/prawns   1. –crabs | | | 1. Milkfish (bangus) 2. Prawn (sugpo) 3. Shrimp (hipon)   14Tilapia  15Crabs (alimango)  16Lobster  17 Threadfin bream (bisugo) | | | | | 1. Blue Marlin (swordfish)   19Moonfish (hiwas)  20Mud fish (dalag)  21Yellow tail fusilier (dalagang bukid)  22Rabbitfish with whitedots (danggit) | | | | | | | 23Anchovy (Dilis)  24Dolphin fish (mahi-mahi)  25Catfish (hito)  26Round scad (galunggong)  27Mackerel  28Grouper (lapulapu) | | | | | | | | 29Red snapper (maya-maya)  30Eel (palos)  31Spanish mackerel (tanigue)  32Mussel (tahong)  33Oyster (talaba)  34Seaweed  35 Tuna  36 Salmon | | | | |
| **C1a.** Please indicate the species of fish that each member caught, volume of fish caught and price per kilo per month**.** | | | | | | | | | | | | | | | | | | | | | | | | | | | | |
|  | **March 2017** | | | | | | **April 2017** | | | | | | **May 2017** | | | | | | | | **June 2017** | | | | | | |  |
| **Classification** | **1-Lean 2-Peak** | | | | | | **1-Lean 2-Peak** | | | | | | **1-Lean 2-Peak** | | | | | | | | **1-Lean 2-Peak** | | | | | | |  |
| **C1aa. Name of member engaged in fishing** | **Species of fish caught** | **Volume of fish caught** | **Farm gate price per kilo** | | | **Market Price per kilo** | **Species of fish caught** | **Volume of fish caught** | | **Farm gate price per kilo** | | **Market Price per kilo** | **Species of fish caught** | **Volume of fish caught** | **Farm gate price per kilo** | | | | **Market Price per kilo** | | **Species of fish caught** | **Volume of fish caught** | **Farm gate price per kilo** | | | | **Market Price per kilo** |  |
|  |  |  |  | | |  |  |  | |  | |  |  |  |  | | | |  | |  |  |  | | | |  |  |
|  |  |  |  | | |  |  |  | |  | |  |  |  |  | | | |  | |  |  |  | | | |  |  |
|  |  |  |  | | |  |  |  | |  | |  |  |  |  | | | |  | |  |  |  | | | |  |  |
|  |  |  |  | | |  |  |  | |  | |  |  |  |  | | | |  | |  |  |  | | | |  |  |
|  |  |  |  | | |  |  |  | |  | |  |  |  |  | | | |  | |  |  |  | | | |  |  |
|  |  |  |  | | |  |  |  | |  | |  |  |  |  | | | |  | |  |  |  | | | |  |  |
|  |  |  |  | | |  |  |  | |  | |  |  |  |  | | | |  | |  |  |  | | | |  |  |
|  |  |  |  | | |  |  |  | |  | |  |  |  |  | | | |  | |  |  |  | | | |  |  |
|  |  |  |  | | |  |  |  | |  | |  |  |  |  | | | |  | |  |  |  | | | |  |  |
|  |  |  |  | | |  |  |  | |  | |  |  |  |  | | | |  | |  |  |  | | | |  |  |
|  |  |  |  | | |  |  |  | |  | |  |  |  |  | | | |  | |  |  |  | | | |  |  |
|  |  |  |  | | |  |  |  | |  | |  |  |  |  | | | |  | |  |  |  | | | |  |  |
|  |  |  |  | | |  |  |  | |  | |  |  |  |  | | | |  | |  |  |  | | | |  |  |
|  |  |  |  | | |  |  |  | |  | |  |  |  |  | | | |  | |  |  |  | | | |  |  |
|  |  |  |  | | |  |  |  | |  | |  |  |  |  | | | |  | |  |  |  | | | |  |  |
|  |  |  |  | | |  |  |  | |  | |  |  |  |  | | | |  | |  |  |  | | | |  |  |
|  |  |  |  | | |  |  |  | |  | |  |  |  |  | | | |  | |  |  |  | | | |  |  |
|  |  |  |  | | |  |  |  | |  | |  |  |  |  | | | |  | |  |  |  | | | |  |  |
|  |  |  |  | | |  |  |  | |  | |  |  |  |  | | | |  | |  |  |  | | | |  |  |
|  |  |  |  | | |  |  |  | |  | |  |  |  |  | | | |  | |  |  |  | | | |  |  |
|  |  |  |  | | |  |  |  | |  | |  |  |  |  | | | |  | |  |  |  | | | |  |  |
|  |  |  |  | | |  |  |  | |  | |  |  |  |  | | | |  | |  |  |  | | | |  |  |
|  |  |  |  | | |  |  |  | |  | |  |  |  |  | | | |  | |  |  |  | | | |  |  |
|  |  |  |  | | |  |  |  | |  | |  |  |  |  | | | |  | |  |  |  | | | |  |  |
|  |  |  |  | | |  |  |  | |  | |  |  |  |  | | | |  | |  |  |  | | | |  |  |
| **CODES: Species of fish** | | | | | | | | | | | | | | | | | | | | | | | | | | | | |
| **C1ab.** Total monthly expense in fishing (material input, labor, transport cost, water & electricity, space rental, other expense | 1- grouper (lapu-lapu)  2- parrot fish  3- emperor  4- snapper  5- sweetlip  6- goatfish  7-surgeonfish  8- squid  9-shrimps/prawns  10–crabs | | | 11Milkfish (bangus)  12 Prawn (sugpo)  13Shrimp (hipon)  14Tilapia  15Crabs (alimango)  16Lobster  17Threadfin bream (bisugo) | | | | | | | 18Blue Marlin (swordfish)  19Moonfish (hiwas)  20Mud fish (dalag)  21Yellow tail fusilier (dalagang bukid)  22Rabbitfish with whitedots (danggit) | | | | | |  | | | 23Anchovy (Dilis)  24Dolphin fish (mahi-mahi)  25Catfish (hito)  26Round scad (galunggong)  27Mackerel  28Grouper (lapulapu) | | | | | | 29Red snapper (maya-maya)  30Eel (palos)  31Spanish mackerel (tanigue)  32Mussel (tahong)  33Oyster (talaba)  34Seaweed  35 Tuna  36 Salmon | | |
| **C1a.** Please indicate the species of fish that each member caught, volume of fish caught and price per kilo per month**.** | | | | | | | | | | | | | | | | | | | | | | | | | | | | |
|  | **July 2017** | | | | | | **August 2017** | | | | | | **September 2017** | | | | | | | | **October 2017** | | | | | | |  |
| **Classification** | **1-Lean 2-Peak** | | | | | | **1-Lean 2-Peak** | | | | | | **1-Lean 2-Peak** | | | | | | | | **1-Lean 2-Peak** | | | | | | |  |
| **C1aa. Name of member engaged in fishing** | **Species of fish caught** | **Volume of fish caught** | **Farm gate price per kilo** | | | **Market Price per kilo** | **Species of fish caught** | **Volume of fish caught** | | **Farm gate price per kilo** | | **Market Price per kilo** | **Species of fish caught** | **Volume of fish caught** | **Farm gate price per kilo** | | | | **Market Price per kilo** | | **Species of fish caught** | **Volume of fish caught** | **Farm gate price per kilo** | | | | **Market Price per kilo** |  |
|  |  |  |  | | |  |  |  | |  | |  |  |  |  | | | |  | |  |  |  | | | |  |  |
|  |  |  |  | | |  |  |  | |  | |  |  |  |  | | | |  | |  |  |  | | | |  |  |
|  |  |  |  | | |  |  |  | |  | |  |  |  |  | | | |  | |  |  |  | | | |  |  |
|  |  |  |  | | |  |  |  | |  | |  |  |  |  | | | |  | |  |  |  | | | |  |  |
|  |  |  |  | | |  |  |  | |  | |  |  |  |  | | | |  | |  |  |  | | | |  |  |
|  |  |  |  | | |  |  |  | |  | |  |  |  |  | | | |  | |  |  |  | | | |  |  |
|  |  |  |  | | |  |  |  | |  | |  |  |  |  | | | |  | |  |  |  | | | |  |  |
|  |  |  |  | | |  |  |  | |  | |  |  |  |  | | | |  | |  |  |  | | | |  |  |
|  |  |  |  | | |  |  |  | |  | |  |  |  |  | | | |  | |  |  |  | | | |  |  |
|  |  |  |  | | |  |  |  | |  | |  |  |  |  | | | |  | |  |  |  | | | |  |  |
|  |  |  |  | | |  |  |  | |  | |  |  |  |  | | | |  | |  |  |  | | | |  |  |
|  |  |  |  | | |  |  |  | |  | |  |  |  |  | | | |  | |  |  |  | | | |  |  |
|  |  |  |  | | |  |  |  | |  | |  |  |  |  | | | |  | |  |  |  | | | |  |  |
|  |  |  |  | | |  |  |  | |  | |  |  |  |  | | | |  | |  |  |  | | | |  |  |
|  |  |  |  | | |  |  |  | |  | |  |  |  |  | | | |  | |  |  |  | | | |  |  |
|  |  |  |  | | |  |  |  | |  | |  |  |  |  | | | |  | |  |  |  | | | |  |  |
|  |  |  |  | | |  |  |  | |  | |  |  |  |  | | | |  | |  |  |  | | | |  |  |
|  |  |  |  | | |  |  |  | |  | |  |  |  |  | | | |  | |  |  |  | | | |  |  |
| **CODES: Species of fish** | | | | | | | | | | | | | | | | | | | | | | | | | | | | |
| **C1ab.** Total monthly expense in fishing (material input, labor, transport cost, water & electricity, space rental, other expense | 1- grouper (lapu-lapu)  2- parrot fish  3- emperor  4- snapper  5- sweetlip  6- goatfish  7-surgeonfish  8- squid  9-shrimps/prawns  10–crabs | | | | 11Milkfish (bangus)  12 Prawn (sugpo)  13Shrimp (hipon)  14Tilapia  15Crabs (alimango)  16Lobster   1. Threadfin bream (bisugo) | | | | | | 18Blue Marlin (swordfish)  19Moonfish (hiwas)  20Mud fish (dalag)  21Yellow tail fusilier (dalagang bukid)  22Rabbitfish with whitedots (danggit) | | | | | |  | 23Anchovy (Dilis)  24Dolphin fish (mahi-mahi)  25Catfish (hito)  26Round scad (galunggong)  27Mackerel  28Grouper (lapulapu) | | | | | | | 29Red snapper (maya-maya)  30Eel (palos)  31Spanish mackerel (tanigue)  32Mussel (tahong)  33Oyster (talaba)  34Seaweed  35 Tuna  36 Salmon | | | |

| **C2** | **Ikaw ba o bisan kinsang sakop sa inyong panimalay nagatampo o nagapartisipar o nahisakop o miembro sa grupo o asosasyon nga dunay kalabutan sa panagat o pag-protektar sa bahandi sa dagat, o sa pag-pangisda sa atong kadagatan?** (Are you or someone in your household is involved/participate or a member of a group or association related to marine or fisheries management?) (**If yes, how many hh member are involved? __Yes. Please name them.**  **__No. ASK C2h.**   \| **C2a. Name of HH member** \| **C2b. Name of Fishery/marine resource management Association** \| **C2c. Purpose of the association/primary activity of the association** \| **C2d. Position or Role or function/tasks in the association** \| **C2e. Number of years as member of the association** \| **C2f. How active is your (his/her) participation in the association?** \| **Time spent in activities of the association** \| **C2g. Reason for being inactive** \| \| --- \| --- \| --- \| --- \| --- \| --- \| --- \| --- \| \|  \|  \|  \|  \|  \|  \|  \|  \| \|  \|  \|  \|  \|  \|  \|  \|  \| \|  \|  \|  \|  \|  \|  \|  \|  \| \|  \|  \|  \|  \|  \|  \|  \|  \| \|  \|  \|  \|  \|  \|  \|  \|  \| \|  \|  \|  \|  \|  \|  \|  \|  \| \|  \|  \|  \|  \|  \|  \|  \|  \| \|  \| **1- TURF-reserve management body**  **2- Fisher’s association**  **3-MPA management Committees**  **4 – Bantay Dagat**  **5-BFARMC**  **6-MFARMC**  **7- Others (specify and describe)** \|  \|  \|  \| **1-always actively involve in all activities of the association**  **2- More or less 75% actively involved in all activities of the association**  **3- More or less 50% actively involved in all activities of the association**  **4 – More or less 25% actively involved**  **5 – Not actively involved at all** \| \|  \|   **C2h. If NOT A MEMBER OF FISHING-RELATED ORGANIZATIONS, ASK: Why no one is a member of fishery or fishery related association? ____________________________________________________________________________________________________________________________________)** | |
| --- | --- | --- | --- | --- | --- | --- | --- | --- | --- | --- | --- | --- | --- | --- | --- | --- | --- | --- | --- | --- | --- | --- | --- | --- | --- | --- | --- | --- | --- | --- | --- | --- | --- | --- | --- | --- | --- | --- | --- | --- | --- | --- | --- | --- | --- | --- | --- | --- | --- | --- | --- | --- | --- | --- | --- | --- | --- | --- | --- | --- | --- | --- | --- | --- | --- | --- | --- | --- | --- | --- | --- | --- | --- | --- |
| **C3** | **Gawas sa mga kapunungan nga dunay kalabutan sa panagat o bahandi sa Dagat, aduna ka ba o bisan kinsa nga miembro sa panimalay nga nahilambigit sa uban pang asosasyon o kalihukan sa Komunidad? Beside organizations/associations that are fishery or marine resource-related,** do you or any member of your household belong to any organization or group?   1. YES.  **Palihug nganli ang miembro ug ang klasi sa kapunungan** 2. NO. SKIP TO C4.  \| **C3a. Name of HH member** \| **C3b. Name of Organization** \| **C3c. Type of Organization (Church, Farmers, Womens, PTCA, Cooperative, etc)** \| **C3d. Is your organization registered with SEC/CDA?** \| **C3e. Do you attend meetings regularly?*** \| \| --- \| --- \| --- \| --- \| --- \| \|  \|  \|  \|  \|  \| \|  \|  \|  \|  \|  \| \|  \|  \|  \|  \|  \| \|  \|  \|  \|  \|  \| \|  \|  \|  \|  \|  \|   Codes: Attend meetings regularly?  1 regularly attend  2 attend sometimes  3 not attending any organizational meetings | |
| **C4** | Did you or any of your household member(s) participate or attend any activity to acquire a particular skill or knowledge in the last 3 years? | Yes-----------------------1 (CONTINUE)  No-------------------------2 (PROCEED TO C5aa) |
| **C5** | **Palihug nganli kung kinsa sila.**Please name those who attended in the training   \| **C5a. Name** \| **C5b. Title/Area of Training** \| **C5c. Sponsor or group managing training** \| **C5d. Inclusive dates** \| **C5e. What did you learn (skills and knowledge)** \| **C5f. Did you apply knowledge and skills learned? How?** \| **C5g. If not applied, why not?** \| \| --- \| --- \| --- \| --- \| --- \| --- \| --- \| \|  \|  \|  \|  \|  \|  \|  \| \|  \|  \|  \|  \|  \|  \|  \| \|  \|  \|  \|  \|  \|  \|  \| \|  \|  \|  \|  \|  \|  \|  \| \|  \|  \|  \|  \|  \|  \|  \| \|  \|  \|  \|  \|  \|  \|  \| | |
| **C5aa** | **Unsa nga klase sa skills training ang imong gusto nga pagahimu-on dinhi sa inyong lugar?** What type of skills training do you want to be conducted in your area?   1. _________________________________________, 2. ______________________________________,3. _______________________________________ | |
| **C6** | **Fishery Management/Resource Management**   \| **Sa imong pagtu-o…………(Do you believed that ….)** \| **Yes** \| **No** \| \| --- \| --- \| --- \| \| 1**. Ang pagpangisda angayan nga ipadagan sa among panimalay nga morag negosyo?** [fishing activities must be run by household like a business.] \|  \|  \| \| 2. **Ang pagpangisda ug isda nga among madakpan nagsubay sa among panginahanglanon sa pagkaon sa panimalay?** [Our fishing activities and the fish we catch is dependent on our food needs in the household.] \|  \|  \| \| 3. **Ang pagpangisda angayan magasubay sa panginahanglanon sa industriya ug merkado?** [Our fishing activities follow the demands of the industry and market] \|  \|  \| \| 4. **Sa miaging mga tuig, nagaanam kadaghan ang isda nga among madakpan?**[During the previouse years, our fish catch has been increasing.] \|  \|  \| \| 5. **Sa miaging mga tuig naga-anam kagamay ang isda nga among madakpan?** [During the previouse years, our fish catch has been declining] \|  \|  \| \| 5. **Angayan gayod nga ang mga mangingisda magasunod sa mga ordinansa ug balaod sa pagpanagat dinhi sa inyong dapit.** [We follow the ordinances and laws on fishing in our area.] \|  \|  \| \| 6. **Nagapananom ba kamo ug kahoy aron dili makalbo ang kabukiran?** [We plant trees in order to prevent forest denudation.] \|  \|  \| \| 7. **Angayan ba nga mogamit ug bag-o apan insakto nga mga teknolohiya sa produksyon sa mga isda**.(Adopted new but appropriate fishing production technologies) \|  \|  \| \| 8. **Nagasunod ang mga mangingisda sa mga pama-agi sa pagpangisda nga maka konserbar ug makapalungtad o sustain sa atong coastal ug marine resources.** (Adopted fishing practices that conserved and sustained the coastal and marine resource) \|  \|  \| | |

**SECTIOND: KNOWLEDGE, ATTITUDE, AND PRACTICES IN NRM**

| **D1.01** | | **D1.02** | **D1.03** | | D1.03a | | | **D1.04** | **D1.05** | |
| --- | --- | --- | --- | --- | --- | --- | --- | --- | --- | --- |
| **Teknolohiyang climate-smart/Praktis**  Climate-Smart Technology/Practice  **Ang mga mosunod nga mga pangutana may kalabutan sa climate-smart technologies o praktis na makakunhod sa dautang epekto sa mga gapangbuhaton sa pang-pangisda sa atong palibot ug sa pag bag-o bag-o sa klima. Akong isulti ang pila ka mga pananglitan ug palihug tubaga ang mga mosunod nga mga pangutana Nakahibalo ka ba mahitungod sa ……..** | | **1-Yes 2-No** | **Unsa ang imong nahibalo-an?**  What do you know […]? | | **Kang kinsa ikaw nakahibalo bahin niini?**  From whom did you learn about this? | | | **Imo ba kini ginapraktis?**  Are you practicing this […]?  **1-Yes 2-No** | **Hinungdan nganong wala kini nimo gi-practice.**  Reason for not practicing? | |
| **A** | **Aquaculture zoning and monitoring for efficient and resilient aquaculture…** kung diin gina paniguro ang insakto nga pagpili sa lugar para mapadaghan ang mga isda sigun sa kondisyon ug kapilgro sa lugar. Lakip na niini ang pagbulag-bulag sa mga fish pens sa insakto nga distansya aron malikayan ang pagtakod-takod sa mga sakit ug mabantayan pag-ayo ang kahimtang sa tubig ug mga isda aron sayon mahibaw-an kung nay mga peste, sakit o lumot. | **1→ 2↓** | |  | | **1-DA**  **2-Province LGU**  **3-Municipal LGU**  **4-Barangay LGU**  **5-Others (specify)**  **_________________** | **1 ↓ 2→** | | |  |
| **B** | **Low energy, fuel-efficient fishing practices…**kung diin gina pagamay ang konsumo sa gasolina pinaagi sa paggamit ug mga insakto nga mga gamit sama sa mga lambat nga dili demakina ug may insakto nga gidak-on(aron dili ma-apil ug kuha ang mga gagmay nga ) , episyente ug “safe” nga disenyo sa mga salakyan pangdagat ug mga kagamitan, apil ang paggamit sa mga materyales nga dali malusaw sa tubig aron malikayan ang ang aksidente sa pagkaligpit sa mga lamang dagat kung mahitabo nga mabilin ang lambat sa tubig. | **1→ 2↓** | |  | | **1-DA**  **2-Province LGU**  **3-Municipal LGU**  **4-Barangay LGU**  **5-Others (specify)**  **_________________** | **1 ↓ 2→** | | |  |
| **C** | **Ecosystem Approach to Fishery Management (EAFM)…** kung diin ang pag-atiman sa kadagatan ug sa mga bahandi niini gipaagi sa usa ka malungtarong sistema o pamaagi. **__________________________________________________________________________________________** | **1→ 2↓** | |  | | **1-DA**  **2-Province LGU**  **3-Municipal LGU**  **4-Barangay LGU**  **5-Others (specify)**  **_________________** | **1 ↓ 2→** | | |  |
| **D** | Adunay mga importanting puluy-anan ang nagkalain-laing klase sa isda ilabi na sa ilang itloganan ug kini angayan nga protektahan (**There are important habitats for species for nursery grounds and spawning sites that need protecting and preserving)** | **1→ 2↓** | |  | | **1-DA**  **2-Province LGU**  **3-Municipal LGU**  **4-Barangay LGU**  **5-Others (specify)**  **_________________** | **1 ↓ 2→** | | |  |
| **E** | Awareness of the effects of the destructive exploitation of coastal resources (Pagkahibalo sa mga epekto sa mga makadaut nga pagpanguha sa mga coastal resources) | **1→ 2↓** | |  | | **1-DA**  **2-Province LGU**  **3-Municipal LGU**  **4-Barangay LGU**  **5-Others (specify)**  **_________________** | **1 ↓ 2→** | | |  |
| **F** | TURF-Territorial use rights for fishing …kung diin ang grupo sa mga mangingisda gihatagan ug prebelihiyo nga makapangisda sa usa ka lugar ug sila nagkahiusa sa pag-atiman sa kadagatan aron kini magmalungtaron | **1→ 2↓** | |  | | **1-DA**  **2-Province LGU**  **3-Municipal LGU**  **4-Barangay LGU**  **5-Others (specify)**  **_________________** | **1 ↓ 2→** | | |  |
| **G** | Sa imaging dose ka bulan (sugod niadtong Nov 2014), aduna bay ubang mga buluhaton nga imong nahibalo-an nga may kalabutan sa pag-uma/pag atiman sa mga hayop/pagpangisda kung diin gi-konsiderar ang proteksyon sa kina-iyahan ug ang pag-andam sa mga dautang epekto sa pagbag-o bag-o sa klima o climate change sa inyong produksyon ug panginabuhi-an? | **1→ 2↓** | |  | | **1-DA**  **2-Province LGU**  **3-Municipal LGU**  **4-Barangay LGU**  **5-Others (specify)**  **_________________** | **1 ↓ 2→↓** | | |  |
|  |  |  |  |  |  |  |  |  |  |  |
|  |  |  |  |  |  |  |  |  |  |  |

| D.2 | Nakahibalo ka ba kung adunay aktibo Marine Protected Area o MPA councils ug komitiba nga maoy responsable sa paggama o pag-gambalay sa mga kalihukan sa pagplano kung unsaon pagpadagan ug pag-atiman sa atong kadagatan? Are you aware of a functioning local marine protected area (MPA) councils and committeesresponsible for the formulation and implementation of Barangay/Municipal Marine Fishing Ground Management Development Plans/enforcement plans. | 1 Yes  2 No. SKIP TO D9.  3 Not sure |
| --- | --- | --- |
| **D.3** | **IF THE ANSWER IS YES**:*Aduna bay mga nakasulat nga mga citeria sa pagpili sa mga sakop sa council/ committee?(Is there an existing written document that describes the composition or terms of reference of the council/committee?)* | 1 Yes  2 No. SKIP TO D5. |
| **D.4** | **IF THE ANSWER IS YES:***Nakahibalo kaba kung adunay nakasulat nga dokumento nga naga saysay o nagapakita sa mga katungdanan /buluhaton sa council/komitiba? (Do you know whether there is a written document that describes the composition orterms of reference of the council/committee?* | 1 Yes  2 No |
| **D.5** | ***Ikaw ba myembro sa local nga marine protected area (MPA) councils ug mga comitiba?***Are you a member of the local marine protected area (MPA) councils and committees? | 1 Yes  2 No. SKIP TO D9 |
| **D.6** | **Sa unsa nga kapasidad?** (In what capacity?) |  |
| **D.7** | **IF THE ANSWER IS YES:*Sa imong hunahuna, ang komposisyon ba sa inyong local marine protected area (MPA) councils ug mga komitibanag representar sa tanang key stakeholders labi na ang mga gagmay o small-scale resource-poor fisherfolks, indigenous peoples o lumad-ug kababayen-an?*** *(In your opinion does the composition of MPA councils and committees represent all key stakeholders especially the small-scale resource-poor fisherfolks, indigenous peoples and women?)* | 1 Yes. Skip to D9  2 No |
| **D.8** | ***Kung wala, unsa ang hinungdan? Unsay angayan buhaton aron kini ma-usab?*** *(*If no, why not? And how to correct the situation?) |  |
| **D.9** | *Aduna ka bay nahibalo-an nga ordinanses ug resolusyon mahitungod sa pagpanagat ug coastal marine management? (*Are you aware of any local legislation/regulations on fishing and coastal marine management?) | 1 Yes  2 No. SKIP TO E1. |
| **D.10** | **IF THE ANSWER IS YES:***Unsa nga mga resolusyon ug ordinansa ang imong nahibalo-an? (*what are these laws and ordinances that you are aware of?) |  |
| **D.11** | ***Sa imong pagtuo mas daghan sa mga lumulupyo niining lugara ang nagasunod o wala nagasupak sa mga bala-od o mga polisiya sa pagpangisda o sa coastal marine management?*** *(Do you believe that there are more residents in this community that adhere and do not* violate the fishing laws and ordinances/coastal marine management policies?) |  |
| **D.12** | *Ngano man? (*Why?) |  |

**SECTION E: ECONOMIC CHARACTERISTICS, HOUSEHOLD INCOME, AND QUALITY OF LIFE**

**I TYPE OF HOUSING MATERIALS (PROXY VARIABLES FOR HOUSEHOLD INCOME)**

|  |  |  | | | |  |
| --- | --- | --- | --- | --- | --- | --- |
| E1 | **Unsay pinakadaghan nga materyales ang gigamit sa salog sa balay?**(Main flooring materials) | Materials pedomonantly used: | | | |  |
|  |  | Earth | | | |  |
|  |  | Makeshift/scrap materials | | | |  |
|  |  | Mixed but predominantly salvaged/scrap materials | | | |  |
|  |  | Light materials (bamboo, sawali, cogon, nipa) | | | |  |
|  |  | Mixed but predominantly light materials | | | |  |
|  |  | Mixed but predominantly strong materials | | | |  |
|  |  | Strong materials (concrete, galvanized iron, brick,  stone, wood, asbestos) | | | |  |
|  |  | Others | | | |  |
| E2 | **Unsay pinakadaghan nga materyales ang gigamit sa bongbong/dingding sa inyong balay sa wala pa gi-ayo ang karsada? Ug Karon?**  (Main materials of wall) | Materials pedomonantly used | | | |  |
|  |  | Earth | | | |  |
|  |  | Makeshift/scrap materials | | | |  |
|  |  | Mixed but predominantly salvaged/scrap materials | | | |  |
|  |  | Light materials (bamboo, sawali, cogon, nipa) | | | |  |
|  |  | Mixed but predominantly light materials | | | |  |
|  |  | Mixed but predominantly strong materials | | | |  |
|  |  | Strong materials (concrete, galvanized iron, brick,  stone, wood, asbestos) | | | |  |
|  |  | Others | | | |  |
| E3 | **Unsay pinakadaghan nga materyales ang gigamit sa atop sa inyong balay?**(Main materials of roof) | Materials pedomonantly used | | | |  |
|  |  | Earth | | | |  |
|  |  | Makeshift/scrap materials | | | |  |
|  |  | Mixed but predominantly salvaged/scrap materials | | | |  |
|  |  | Light materials (bamboo, sawali, cogon, nipa) | | | |  |
|  |  | Mixed but predominantly light materials | | | |  |
|  |  | Mixed but predominantly strong materials | | | |  |
|  |  | Strong materials (concrete, galvanized iron, brick,  stone, wood, asbestos) | | | |  |
|  |  | Others | | | |  |
| **II HOUSEHOLD ASSETS AND AMENITIES (PROXY VARIABLES FOR HOUSEHOLD INCOME)** | | | | | | |
| E4 | **Unsa nga mga butang o kabtangan (appliances) ang inyong gipanag-iya o’ napalit?Unsay imong gipanag-iya sapagkakaron? Unsa ang gidaghanon niini?** (What are the hh appliances that you owned? How many do you own now?)  **Aduna ka bay _____________?** (Do you have __________?) **Pila ka** _________**__ ang inyong gipanag-iya?**(How many of ________ do you own?)  **(Appliances may be for hh purposes or business)** | | | **Kindly encircle and write the quantity of appliances or size of property in the space provided**   \| Appliances \| Number Owned \| \| --- \| --- \| \|  \| \| Electric fan \|  \| \| Cellphone \|  \| \| Electric/gas stove \|  \| \| Karaoke/cassete recorder \|  \| \| Bicycle/sikad \|  \| \| Sala set \|  \| \| Tv/betamax/VHS \|  \| \| Refrigerator \|  \| \| Washing machine/laundry dryer \|  \| \| Sewing machine \|  \| \| Aircon \|  \| \| Appliances \|  \| \|  \| \| Gas or electric range/oven \|  \| \| Computer \|  \| \| Electric organ/piano \|  \| \| Motorcycle \|  \| \| Car/motor vehicle \|  \| \| Motorized boat \|  \| \| Non-motorized boat \|  \| \| Others (specify) \|  \| \| Livestock & Poultry \|  \| \| Carabao \|  \| \| Cow \|  \| \| Horse \|  \| \| Pig \|  \| \| Chicken \|  \| \| Others (specify) \|  \| | | |
| E5 | **OWNERSHIP STATUS OF HOUSE** | | 1. Owned **(ASK E6 & then E7)** 2. Rented. **GO TO E6then to E8** 3. Rent to own. **GO TO E6then to E8** 4. Others Arrangement (specify) _____ **GO TO E6 then to E8** | | | |
| E6 | ***Kung inyo pa kining girentahan ang balay, pila kaha kini?*** | | **________Rent per month** | | | |
| E7 | **Kinsa sa inyong sakop sa panimalay ang nanag-iya sa balay nga inyong gipuy-an?** (Who among your household member own the house you were residing?)   \| Name of HH member who owns the house \| **Kanus-a niya napalit o naangkon kini nga balay nga gipuy-an?**(When did you/he/she acquire this house? \| \| --- \| --- \| \|  \|  \| \|  \|  \| \|  \|  \| \|  \|  \| | | | | | |
| E8 | **OWNERSHIP STATUS OF LOT** | | | 1. Owned. **ASK E9 & then E10** 2. Rented. **GO TO E9 then to E11** 3. Rent to own. **GO TO E9then to E10** 4. Others Arrangement (specify) _____**GO TO E9 then to E10** | | |
| E9 | **Kung kining yuta inyo pang girentahan, pila kaha kini?** | | | **________Rent per month** | | |
| E10 | **Kinsa sa inyong sakop sa panimalay ang nanag-iya niining yuta nga gitarokan sa inyong pinuy-anan?**(Who among your household member own the land where the house you were residing was built?)   \| Name of HH member who owns the lot where house is built \| **Kanus-a niya napalit o naangkon kini nga yuta?** (When did he/she acquire this house? \| \| --- \| --- \| \|  \| \|  \|  \| \|  \|  \| \|  \|  \| | | | | | |
| E11 | **Unsa nga klase sa kasilyas ang inyong gigamit sa panimalay?***(*What kind of toilet facility does your household use?) | | | | [1] Own flush toilet  [2] Shared flush toilet  [3] Water Sealed  [4] Close pit toilet  [5] Open pit toilet  [6] No toilet/River/Field/Bush  [7] Others. Specify _____________________ | |

| E12 | **Unsay inyong gagamiton nga sugnod sa pagluto?** (What is your fuel for cooking?) | 1. Firewood/wood/charcoal   2 Electricity  3 Acyteline Gas (e.g, Shellane, Pryce Gas, Gasul)  4 Kerosine  5 Others (Specify) ____________________ |
| --- | --- | --- |
| E13 | **Gakaon ba kamo katulo sa usa ka adlaw sa imaging 7 ka adlaw?(pamahaw, pani-udto ug panihapon)?(**Do you eat 3 full meals a day during the last 7 days?) | 1 Yes  2 No. Why not? _______________________________ |
| E14 | **Aduna bay higayon nga kamo nakulangan/nagproblema kabahin sa pagkaon sa panimalay sa miaging 12 ka bulan?** (Was there an instance that you did not have enough food/had problems meeting your food requirements during the last 12 months?) | 1. YES 2. No **(GO TO E18)** |
| E15 | **Pila ka adlaw sa usa ka tuig ang inyong panimalay nagkulang/naglisod sa pagkaon?**(How many days in one year did you have problems meeting your food requirements/did not have enough food?) | ________Days |
| E16 | **Giunsa ninyo pagsulbad ang kakulangon sa pagkaon?** (How did you cope with the problem of food insufficiency?) | 1– Nanghulam/nangutang  2– Gikulangan ang gidaghanon sa gipreparar nga pagkaon  3 -Others (specify) __________________ |
| E17 | **Kung nagkulang kamo sa pagkaon, kinsa sa mga myembro sa panimalay ang mas naapiktuhan (kulang ang bahin sa pagkaon)?** (If food is insufficient, who are the HH members most affected?)   \| Name of HH Member or ID number of member \| **Nganong si ________ man ang mas naapiktuhan (kulang ang bahin sa pagkaon o’ wala gyud mokaon) sa kakulangon sa pagkaon?** (Why was __________ most affected by insufficiency of food in the household?) \| \| --- \| --- \| \|  \|  \| \|  \|  \| \|  \|  \| \|  \|  \| \|  \|  \| | |

| E18 | *Karon, gusto akong mangutana mahitungod sa mga pagka-on nga gika-on sa mga myembro sa imong panimalay. Asa sa mga mosunod ang nagahulagway sa pagka-on nga gika-on sa mga myembro sa panimalay sa miaging 12 ka bulan?*  (Now I would like to ask a couple of questions about the food eaten in your household. Which of these  statements best describes the food eaten in your household in the last 12 months?) | [1] We always have enough food to feed everyone in the household.  [2] We sometimes do not have enough food to feed everyone in the household.  [3] We often do not have enough food to feed everyone in the household.  [4] We never have enough food to feed everyone in the household  **[*If ‘always had enough,’ skip to question E20. If ‘sometimes did not have, often did not have or never had***  ***enough,’ proceed to question E19.*]** | |
| --- | --- | --- | --- |
| E19 | Ang mga mosunod mga rason kung ngano ang ubang tawo dili kanunay nga adunay igo nga katakos sa pagpakaon sa mga sakop sa panimalay. Asa sa mga mosunod ang imong masulti ang mga rason kung nganong ang imong pamilya dili kanunay adunay igo nga pagka-on? Here are some reasons why people do not always have enough to feed everyone in their household. Foreach one, please tell me if that is a reason why *your household* does not always have enough to eat.  **[*Respondents can select as many as apply.*]** | [1] We do not always have enough money / our earnings are not always enough  [2] There are too many people to feed.  [3] It is sometimes too hard to get to the market/store.  [4] There is not always enough food available at the market/store.  [5] There is not enough food during certain seasons/times of year.  [6] Other, Ask: please specify the reason why your household did not alwayshave enough to eat ____________________ | |
| E20 | *Kompara sa miaging 5 ka tuig, ang kalidad sa pagpanarbaho sa mga myembro sa panimalay na…..*  Compared to 5 years ago, the quality and stability of employment for my household has: | | (1) Worsened substantially  [2] Worsened somewhat  [3] Stayed the same  [4] Improved somewhat  [5] Improved substantially  [6] Don’t know |
| E21 | *Palihug ug tubag sa mga mosunod gamit ang ang-angan sa hagdanan nga adunay ang-ang nga 1 paingon sa 10, kung diin ang 1 nagpasabot nga dili gyud ka kontento ug and 10, kontento ka kaayo. Sa tanang aspeto, unsa ikaw ka kontento sa imong kinabuhi karon?*  (Please answer the following question on a scale of 1 to 10, where 1=completely dissatisfied, and 10=completely satisfied.“All things considered, how satisfied are you with your life these days?”) | | **Completely satisfied**  10  9  8  7  6  5  4  **3**  **1- completely dissatisfied**  **2** |

| E22 | Palihug ako sultihi sa imong opinyon. Ikaw ba uyon o supak sa mga mosunod?  Please tell me your opinion, do you agree or disagree to the following statement. CHECK APPROPRIATE BOX.   \| Items \| Strongly Agree \| Agree \| Neither Agree nor Disagree \| Disagree \| Strongly Disagree \| \| --- \| --- \| --- \| --- \| --- \| --- \| \| 1. ***Sayon para kanako ug sa ubang sakop sa panimalay nga makakita sa gikinahanglang trabaho para matubag ang panginahanglanon sa panimalay.***It is easy for myself and members of my household to find as much employment as is needed to provide for the household. \|  \|  \|  \|  \|  \| \| 2. **Ang local nga pangagamhanan masaligan sa paghimo ug mga desisyon alang sa kaayohan sa komunidad.**Generally speaking, the [local government] can be trusted to make decisions in the best interest ofour community. \|  \|  \|  \|  \|  \| \| 3. **Ang kadaghanan sa mga tawo dinhi masaligan.**Generally speaking, most people in [my community] can be trusted. \|  \|  \|  \|  \|  \| \| 4. **Patas o walay pagpihig ang balaod sa mga tawo dinhi sa barangay o komunidad.** (People in this barangay/community are treated fairly by the law) \|  \|  \|  \|  \|  \| \| 5. ***Ang akong panimalay nakapahimulos sa mga abot sa among dagat parehas sa ubang mga myembro sa komunidad.***  My household is able to benefit from [our community’s fishery] as much as any other member of the community. \|  \|  \|  \|  \|  \| \| 6. **Kining komunidad daghang krimen batok sa batang babae ug kababayen-an sulod sa panimalay.** (In this barangay/community, there is much violence against girls and women within the households) \|  \|  \|  \|  \|  \| \| 7. **Kining komunidad/barangay adunay daghan krimen o pagsupak sa pamalaod o ordinansa kabahin sa pagpanagat o pagpangisda?** (In this community, there is much violation in fishing ordinances) \|  \|  \|  \|  \|  \| \| 8. **Ang kadaghanan sa katawhan sa among komunidad motabang sa pag implementar sa pama-agi ug regulasyon sa pagpangisda lakip na ang pag sumbong sa mga bayulasyon nga ilang makit-an. (**Most people in [my community] will actively enforce the rules and regulations of [our fishery],including reporting violations when witnessed.) \|  \|  \|  \|  \|  \| \| 9. **Ang among komunidad adunay katakos sa pag-atiman sa among kadagatan/pangisdaanaron kami makapahimulos niini sa uma-abot.**  .[My community] has the ability to sustainably manage [our fishery] so that we can benefit from it long into the future. \|  \|  \|  \|  \|  \| \| **10. Ang mga komitiba nga gisaligan para magpadagan sa among MPA o coastal resource management masaligan nga mohimo sa mga angayan buhaton alang sa malungtaron nga pag-atiman sa atong kadagatan ug lugar pangisdaan?**  (The committees or body that manage the MPA or our coastal resource management will make right decisions about managing our fishery and fishing grounds) \|  \|  \|  \|  \|  \| |
| --- | --- | --- | --- | --- | --- | --- | --- | --- | --- | --- | --- | --- | --- | --- | --- | --- | --- | --- | --- | --- | --- | --- | --- | --- | --- | --- | --- | --- | --- | --- | --- | --- | --- | --- | --- | --- | --- | --- | --- | --- | --- | --- | --- | --- | --- | --- | --- | --- | --- | --- | --- | --- | --- | --- | --- | --- | --- | --- | --- | --- | --- | --- | --- | --- | --- | --- | --- |

| **E23** | Aduna ka bay tinigom o bisan kinsa nga miembro sa panimalay and adunay tinigom?   \| E23a. Name \| E23b. Regular nga gatigom \| E23c. Pila man and kantidad kada nimo tigom? \| E23d. Asa nimo gibutang and imong tinigom? \| E23e. Total amount of savings as of Oct 30, 2017 \| E23f. Asa gikan ang imong tinigom \| E23g. Unsay imong gamitan sa imong tinigom \| \| --- \| --- \| --- \| --- \| --- \| --- \| --- \| \|  \|  \|  \|  \|  \|  \|  \| \|  \|  \|  \|  \|  \|  \|  \| \|  \|  \|  \|  \|  \|  \|  \| \|  \|  \|  \|  \|  \|  \|  \| \|  \|  \|  \|  \|  \|  \|  \| \|  \|  \|  \|  \|  \|  \|  \| \|  \|  \|  \|  \|  \|  \|  \| | |
| --- | --- | --- | --- | --- | --- | --- | --- | --- | --- | --- | --- | --- | --- | --- | --- | --- | --- | --- | --- | --- | --- | --- | --- | --- | --- | --- | --- | --- | --- | --- | --- | --- | --- | --- | --- | --- | --- | --- | --- | --- | --- | --- | --- | --- | --- | --- | --- | --- | --- | --- | --- | --- | --- | --- | --- | --- | --- | --- |
| **E24** | Aduna ka bay utang/loan/balayronon o bisan kinsa nga miembro sa imong panimalay ang dunay utang o balayronon   \| E24a. Name \| E24b. Regular nga ga loan o gapangutang \| E24c. Asa ka man ga loan/gapangutang? \| E24d. Pila man ang kantidad kada nimo loan/pangutang? \| E24e. Total amount of loan or credit as of Oct 30, 2017 \| E24f. Unsay sistema o paagi sa pagbayad niini \| E24g. Unsay gigamitan sa loan o giutang \| \| --- \| --- \| --- \| --- \| --- \| --- \| --- \| \|  \|  \|  \|  \|  \|  \|  \| \|  \|  \|  \|  \|  \|  \|  \| \|  \|  \|  \|  \|  \|  \|  \| \|  \|  \|  \|  \|  \|  \|  \| \|  \|  \|  \|  \|  \|  \|  \| \|  \|  \|  \|  \|  \|  \|  \| | |
| **E25** | Ako o bisan kinsa nga miembro sa akong panimalay adunay kakuhaan sa loan o kautangan kung kami nangihanglan? | 1 Yes  2 No |
| **E26** | Asa man gikan o kinsa man ang magpaloan o mag-pautang | Source:  1 Bank/lending institution/Coops  2 Office/place of work  3 Relatives/friends/neighbors  4 usurers/Bombay  5 |

***Section F. Access to Relevant Services***

| **F1** | What services do you know and have accessed to from the provincial, municipal, barangay government and private institutions?   \| **Type of Services** \| **Know that Services are available (yes; no)** \| **Source of services** \| **Have you access these services in 2016? (yes; no)** \| **Satisfied with the quality of services** \| \| --- \| --- \| --- \| --- \| --- \| \| **1.** Trading post/center,-Food Terminal, -Auction market \|  \|  \|  \|  \| \| **2. Marketing support** \|  \|  \|  \|  \| \| **3. Fish docking Area** \|  \|  \|  \|  \| \| **4. Cold storage** \|  \|  \|  \|  \| \| **5 Fingerlings** \|  \|  \|  \|  \| \| **6 fish cages** \|  \|  \|  \|  \| \| **7 credit facillities** \|  \|  \|  \|  \| \| **8 Cooperative credit support** \|  \|  \|  \|  \| \| **9 Potable Water Supply** \|  \|  \|  \|  \| \|  \|  \|  \|  \| 1. Very satisfied 2. Satisfied 3. Somewhat satisfied 4. Not satisfied \| | | | |
| --- | --- | --- | --- | --- | --- | --- | --- | --- | --- | --- | --- | --- | --- | --- | --- | --- | --- | --- | --- | --- | --- | --- | --- | --- | --- | --- | --- | --- | --- | --- | --- | --- | --- | --- | --- | --- | --- | --- | --- | --- | --- | --- | --- | --- | --- | --- | --- | --- | --- | --- | --- | --- | --- | --- | --- | --- | --- | --- | --- |
| **F2** | **Narinig na po ba ninyo ang tungkol sa mga programang ito?**  Have you heard of the following program? | **Oo →**  Yes → | **Hindi ↓**  No↓ | **Kayo po ba o isa man sa miyembro ng sambahayang ito ay benipisyaryo noong 2014 ng alinmang sa mga sumusunod na programang aking babanggitin?**  Is anyone in this household a beneficiary of [PROGRAM]?  1 **Oo, nakatatanggap ng mga benepisyo noong 2014**YES, RECEIVED BENEFITS IN 2014  2. **wala** NO |
|  | 1. PantawidPamilyang Pilipino Program (4Ps) |  |  |  |
|  | 1. Self-Employment Assistance Kaunlaran (SEA-K) |  |  |  |
|  | 1. Healthy Start Feeding Project (HSFP) ( ages 3 to 5 yrs old) |  |  |  |
|  | 1. PhilHealth Indigent Program or any other Health Insurance plan from PhilHealth |  |  |  |
|  | 1. Food for School Project/ Other feeding Project in the Barangay ( Grades 1 to 6 ) |  |  |  |
|  | 1. Food for Work Project |  |  |  |
|  | 1. Cash for Work Project |  |  |  |
|  | 1. Kahit anong Scholarship Program |  |  |  |
|  | 1. Kahit anong programa ng Congressmen/Governor/Mayor: _________________ |  |  |  |
|  | 1. May alam po ba kayong mga programa ng ahensya ng gobyerno tulad ng DAR, DTI, DENR, DOST atbp. o kahit anong pribadong grupona may kinalaman sa pangngisda na isinasagawa noong 2014 |  |  |  |
|  | Name of program:____________________________ Agency:______________ |  |  |  |
|  | Name of program:____________________________ Agency:______________ |  |  |  |
|  | 1. Others, specify: ________________ |  |  |  |

**SECTION G. GENDER EQUALITY AND SOCIAL INCLUSION**

| G1. | **Pila ka babaye nga miembro sa panimalay ang adunay trabaho o pangita** (How many women members of the household wereworking?) | **__________Females** |
| --- | --- | --- |
| G2. | **Palihug nganli ang mga babae sa panimalay ug unsa ang ilang trabaho sa tibuook adlaw sulod sa 24 ka oras?**(Please name the women in the Households and their activities and work in 24 hours in a day?) FOR WOMEN 4 YEARS OLD AND OVER ONLY.   \| **G2a. Name** \| **G2b. Number of hours per day spent in work or livelihood activities** \| **G2c. Number of hours per day spent in household chores/**  **Child care** \| **G2d. Number of hours spent in helping spouse in his or her economic activities** \| **G2e. Number of hours spent in other activities** \| **Specify type of other activities** \| \| --- \| --- \| --- \| --- \| --- \| --- \| \|  \|  \|  \|  \|  \|  \| \|  \|  \|  \|  \|  \|  \| \|  \|  \|  \|  \|  \|  \| \|  \|  \|  \|  \|  \|  \| | |

| G3 | **Gender and Development**  **Palihug sultii ako kung uyon ka ba niining mosunod nga mga panghunahuna**  (Please tell me your opinion: whether you agree or disagree to the following statements :)   \| **Items** \| **Strongly Agree** \| **Agree** \| **Niether Agree nor disagree** \| **Disagree** \| **Strongly Disagree** \| **Don’t Know** \| \| --- \| --- \| --- \| --- \| --- \| --- \| --- \| \| 1.**Ang mga kababayen-an lang ang responsible sa mga trabaho sa balay ug sa pag-atiman niini (**The women are the ones responsible for the household chores and management) \|  \|  \|  \|  \|  \|  \| \| 2. **Ang mga kalalakin-an lang ang responsible sa mga trabaho sa balay ug sa pag-atiman niini.** (The husbands are the ones responsible for the household chores and management) \|  \|  \|  \|  \|  \|  \| \| 3. **Ang mga kababayen-an ang gaatiman ug asikaso sa mga anak** (The wife is the one taking care of the children) \|  \|  \|  \|  \|  \|  \| \| 4. **Ang mga kalalakin-an ang gaatiman ug asikaso sa mga anak** (Ang kalalakihan ang nangangalaga sa mga anak. (The husbands take care of the children) \|  \|  \|  \|  \|  \|  \| \| 5. **Ang asawa adunay proyektong panginabuhian** (The wife has livelihood projects) \|  \|  \|  \|  \|  \|  \| \| 6.**Ang bana lang ang gatrabaho o nagapangisda**(The husband is the only one working and fishing) \|  \|  \|  \|  \|  \|  \| \| 7. **Ang asawa lang ang miyembro sa organisasyon sa komunidad** (The wife is a member of community organizations) \|  \|  \|  \|  \|  \|  \| \| 8. **Ang bana lang ang miembro sa organisasyon sa komunidad** (The husband is the only member in community organizations in the household) \|  \|  \|  \|  \|  \|  \| | |
| --- | --- | --- | --- | --- | --- | --- | --- | --- | --- | --- | --- | --- | --- | --- | --- | --- | --- | --- | --- | --- | --- | --- | --- | --- | --- | --- | --- | --- | --- | --- | --- | --- | --- | --- | --- | --- | --- | --- | --- | --- | --- | --- | --- | --- | --- | --- | --- | --- | --- | --- | --- | --- | --- | --- | --- | --- | --- | --- | --- | --- | --- | --- | --- | --- | --- |
| G4 | **Unsay kaakuhan/responsibilidad sa mga babaye nga miembro sa panimalay sa pagpanagat o mga kalihukan nga dunay kalabutan sa pagpanagat? (What is the role of women household members in fishing or in fishery?)** |  |

**END OF INTERVIEW**

**THANK RESPONDENT**
