# Supplementary material for: Catalyzing sustainable fisheries management through behavior change interventions
Source: Conserv Biol. 2020 Apr 15;34(5):1176–89. doi: 10.1111/cobi.13475 (PMC7540413; doi:10.1111/cobi.13475)
Supplement: Supplementary file 19 — Supplementary Material [file COBI-34-1176-s019.docx]

Pride Campaign

Survey 2016

Form No: ______________________

Name of Interviewer:_____________

Time Started:___________________

Time Ended: ___________________

Encoding No:___________________

INTRODUCTION (to be read by Enumerator)

Hello, my name is _______________________, and I am working with our local government. We are conducting a survey of residents about our naturalk environment. We would very much appreciate your participation in this survey by answering a few questions about the coastal resources and fisheries.

Whatever information you provide will be kept strictly confidential and your name and answers will not be shown to or shared with any other person except for those people who are working on the survey. Your answers will help us to plan and impleent conservation programs in our community.

Participation in this survey is VOLUNTARY and you can choose not to answer any individual question or all of the questions. However, your views are important to us and I hope you participate.

May I begin the interview now?

Respondent agrees to be interviewed (If NO. keep tally on separate sheet of paper)

[ ] Yes [ ] No

Do you fish or have done gleaning activities? (If NO, DO NOT PROCEED)

[ ] Yes [ ] No

Are you 16 years old or older? (If NO, DO NOT PROCEED)

[ ] Yes [ ] No

(Pre-filled prior to Interview)

BACKGROUND INFORMATION

ENUMERATION AREA:

[ ] Masinloc [ ] Palauig [ ] Mercedes [ ] Gubat [ ] Looc [ ] Lubang [ ] Libertad [ ] Pandan [ ] Culasi [ ] Tayasan [ ] Manjuyod [ ] San Carlos [ ] Dapa [ ] General Luna [ ] Del Carmen

Survey Period:

[ ] FF Baseline 2016 [ ] FF Post KAP 2017

Section 1

Socio-economic and Demographic Questions

"To begin, I would like to ask you some questions about yourself"

(1). How old are you?

[ ] 15 - 19 [ ] 20 - 24 [ ] 25 - 29 [ ] 30 - 34 [ ] 35 - 39 [ ] 40 - 44 [ ] 45 - 49 [ ] 50 - 54 [ ] 55 or older

(2). What is your highest level of education completed (CHOOSE ONE ONLY)

[ ] No School Completed [ ] Pre-School [ ] Elementary [ ] High School [ ] Trade/Vocational [ ] College [ ] Post Graduate (Masteral, PhD) [ ] Refuse to answer [ ] Other ________________

(3) How would you consider yourself?

[ ] Full-time Fisher [ ] Part-time Fisher

(3A) For part-time fishers, what is your main occupation or sector in which you work? (CHOOSE ONE ONLY)

[ ] Agriculture [ ] Office Work [ ] Professional (example: Teacher [ ] Transportation (shipping, trucking, rail) [ ] Student [ ] Small business (shopkeeper or salesperson) [ ] Factory or Manufacturing [ ] Carpentry or daily wage earner [ ] Housework [ ] Self-employed [ ] Refuse to answer [ ] Other ________________

(4). What type of gears do you use? (CHECK ALL THAT APPLY)

[ ] Nets [ ] Lines [ ] Traps [ ] Hand Instruments [ ] Mixed gears [ ] Gleaning [ ] Other ________________

(5). What kind of fishing vessel do you use? (CHECK ALL THAT APPLY)

[ ] Motorized: ________ Horsepower (HP) [ ] Non-motorized [ ] Refused to answer [ ] Other ________________

(6). Do you own the fishing vessel you are using?

[ ] Yes [ ] No

(7). Which month of the year do you fish? (CHECK ALL THAT APPLY)

[ ] January [ ] February [ ] March [ ] April [ ] May [ ] June [ ] July [ ] August [ ] September [ ] October [ ] November [ ] December

(8). On average, what is your best estimate of your household monthly income earned by all family memebers that currently live in the same home with you? DO NOT INCLUDE REMITTANCES

[ ] Below 1,500 Pesos [ ] Between 1,501 to 5,000 [ ] Between 5,001 to 10,000 [ ] Between 10,001 and 15,000 [ ] Greater than 15,001 [ ] Refused to answer [ ] Not sure

(9). What percentage of this monthly income comes from fishing/gleaning? (READ ALL OPTIONS, CHOOSE ONLY ONE OPTION).

[ ] All [ ] More than a half but not all [ ] Half [ ] One fourth [ ] None [ ] Refused to answer [ ] Other ________________

(10). Approximately how much of your catch each day do you keep/consume for your family? READ ALL OPTIONS, CHOOSE ONLY ONE OPTION.

[ ] All [ ] More than a half but not all [ ] Half [ ] One fourth [ ] None [ ] Refuse to answer [ ] Other ________________

(11). Are you a member of any fisher association?

[ ] Yes [ ] No [ ] Do not know/Unsure [ ] Refused to answer

(12). Are you a member of or participate in a savings clubs club or business association group?

[ ] Yes [ ] No [ ] Do not know/Unsure [ ] Refused to answer

Section 2

Establish Baselines and Measure Change in Knowledge SMART Objectives

Now, I would like to ask you some questions about the local environment and marine resources in this area.

(13). I am going to ask you about the marine sanctuary that is going to be or has been created in your local area. A marine sanctuary or no-take fishing area is a clearly identified area where fishing is not allowed at any time by anyone.

(13A). Are there any benefits to the local community from having a marine sanctuary nearby? (IF respondents answers YES then please also NEXT QUESTION)

[ ] Yes [ ] No

(13B) If YES, can you tell me up to 3 benefits that you know. (DO NOT read out answers but tick as appropriate or add to OTHERS).

[ ] more fish [ ] area for fish to reproduce [ ] bigger fish [ ] better coral health/habitat for fish [ ] food security [ ] eco-tourism [ ] Other ________________ [ ] N/A

(14). Please state below whether you beleive each regulation is either TRUE, FALSE or UNSURE about marine sanctuary rules and regulations and fisheries management in your municipality.

(A). No-one can fish or collect shells inside a marine sanctuary

[ ] True [ ] False [ ] Unsure

(B) Fishers are allowed to fish outside the smarine sanctuary

[ ] True [ ] False [ ] Unsure

(C) After 5 years of no fishing being allowed, the marine sanctuary can be harvested again

[ ] True [ ] False [ ] Unsure

(D) Are there benefits of a registered fisher?

[ ] True [ ] False [ ] Unsure

(E) Are there benefits of having a fishing gear license?

[ ] True [ ] False [ ] Unsure

(F) Are there benefits of having yur boat registered?

[ ] True [ ] False [ ] Unsure

(G) Are there benefits of reporting your catch?

[ ] True [ ] False [ ] Unsure

(H) Have you heard the term "Territorial User rights for fishing (TURF) or Managed Access Area plus Sanctuary (MAA+S)?

[ ] True [ ] False [ ] Unsure

Section 3

Establish Baselines and Measure Change in Attitudes SMART Objectives

(15) Please state below whether you STONGLY AGREE, AGREE, DISAGREE and STRONGLY DISAGREE with the following statements on the impacts of having a marine sanctuary on the community.

(A) Fish species will continue to decline in number or disappear if the marine sanctuary rules/policies are not strictly observed by fishers.

[ ] Strongly Agree [ ] Agree [ ] Disagree [ ] Strongly Disagree [ ] Refused to Answer

(B) Protecting the marine sanctuary is beneficial for increasing fish abundance in the municipality

[ ] Strongly Agree [ ] Agree [ ] Disagree [ ] Strongly Disagree [ ] Refused to Answer

(C) Hook and line fishing is the only allowed gear to use outside/bufferzone in marine sanctuaries

[ ] Strongly Agree [ ] Agree [ ] Disagree [ ] Strongly Disagree [ ] Refused to Answer

Section 4

Establish Baselines and Measure Change in Interpersonal Communication SMART Objectives

(16). In the past 6 months, have you talked to anyone about the benefits to the community of a well-enforced sanctuary? If YES, please tell me if you talked to the following people. If NO, check "have not talked to anyone."

[ ] Have not talked to anyone [ ] Talked to a family member [ ] Talked to neighbors and fellow fishers [ ] Talked to local barangay and municipal leaders [ ] Refused to answer [ ] Other ________________

(16A). If you talked about this, an you tell me what the main benefit that was discussed? (DO NOT READ ANSERS BUT TICK OFF CLOSEST ANSWER OR ADD IN "OTHERS")

[ ] more fish [ ] area for fish to produce [ ] bigger fish [ ] better coral heslth/habitat for fish [ ] food security [ ] ecotourism [ ] Not applicable (TICK this when respondent answered NO in Question a) [ ] Other ________________

(17) In the past 6 months, have you talked to anyone about the importance of not fishing inside the marine sanctuary?

[ ] Yes [ ] No [ ] Unsure

(18) In the last 6 months, have you talked to anyone about the importance of fisher registation?

[ ] Yes [ ] No [ ] Unsure

(19) In the last 6 months, have you ever talked to anyone about the importance of attending meetings about fisheries management?

[ ] Yes [ ] No [ ] Unsure

(20). In the last 6 months, have you ever talked about the importance of reporting fish catch?

[ ] Yes [ ] No [ ] Unsure

Section 5

Establish Baselines and Measure Change in Behavior SMART Objectives

(21). Over the past 6 months, which of the following options best describe you about fisherfolk registration (Fish-R)?

[ ] I do not know about fisher registration [ ] I am considering applying for fisherfolk registration [ ] I intend to apply for a fisherfolk registration [ ] I have applied for a fisherfolk registration [ ] I have renewed my fisherfolk registration

(22). Over the past 6 months, which of the following options best describes you about boat registration (Boat-R)?

[ ] I do not know about boat registration [ ] I am considering applying for boat registration [ ] I intend to apply for boat registration [ ] I have applied for boat registration [ ] I have renewed my boat registration

(23). Over the past 6 motnhs, which of the following options best describes you about fishing gear licensing?

[ ] I do not know about fishing gear license [ ] I am considering applying for fishing gear license [ ] I intend to apply for fishing gear license [ ] I have applied for fishing gear license [ ] I have renewed my fishing gear license

(24). Over the past 6 months, which of the following options best describe you about measuring your catch and reporrting it?

[ ] I have never considered measuring my catch and reporting it [ ] I am considering measuring my catch and reporting it [ ] I intend to measure my catch and reporting it [ ] I have currently oarticipated in catch reporting and reporting

(25). Over the past 6 months, which of the following best describes you about attending discussing and meetings on fisheries management?

[ ] I do not know about the discussions and meeting on fisheries management [ ] I am considering n participating discussions and meetings on fisheries management [ ] I intend to participate in discussion and meetings on fisheries management [ ] I have already participating in discussions and meetings on fisheries management

(26). Over the past 6 months, which of the following options best describes you about following/complying to fishing rules and regulations of the management access + sanctuary?

[ ] I do know about the fishing policies of MAA+S [ ] I am considering in complying with the fishing policies of MAA+S [ ] I intend in complying with the fishing policies of MAA+S [ ] I am complying with the fishing policies of MAA+S

(27). In the past 6 months, please tell me if you have done the following actions:

A. Registered my fishing gears in the municipal fisheries office

[ ] Yes [ ] No [ ] No Answer

B. Registered my boat

[ ] Yes [ ] No [ ] No Answer

C. Reporting my catch

[ ] Yes [ ] No [ ] No Answer

D. Attending meetings and consultations about fisheries

[ ] Yes [ ] No [ ] No Answer

E. Use of allowed fishing gear in designated area outside the marine sanctuaries

[ ] Yes [ ] No [ ] No Answer

Section 6

Climate Change

I will be asking you questions related to climate change. Climate change is defined as abrupt changes in our weather patterns that makes typhoons stronger or prolonging droughts.

(28) I will read a series of statements related your belief related to climate change. Please tell me if you strongly agree (SA), Agree (A), Disagree (D) Strongly Disagree (SD), Don't Know (DK).

(A). Climate change is not going to cause any problems in my community

[ ] Strongly Agree [ ] Agree [ ] Disagree [ ] Strongly Disagree [ ] Do not Know

(B). Climate change is already a problem in my community.

[ ] Strongly Agree [ ] Agree [ ] Disagree [ ] Strongly Disagree [ ] Do not Know

(C). Climate change is likely to become a real problem for my community in the coming 5-10 years.

[ ] Strongly Agree [ ] Agree [ ] Disagree [ ] Strongly Disagree [ ] Do not Know

(D). If marine sanctuaries are well-managed, it will help buffer some of the effects of climate change in the future.

[ ] Strongly Agree [ ] Agree [ ] Disagree [ ] Strongly Disagree [ ] Do not Know

(29). Name up to 3 ways which you beleive cliamte change could affect your community in the next 5-10 years (DO NOT READ OUT OPTIONS - SELECT UP TO 3).

[ ] sea level rise [ ] increase water temperature leading to death of corals and less fish [ ] increase of typhoon frequency [ ] increase of typhoon strength, less reliable work and crops [ ] more people from uplad will move to the coastal areas in case of crop failure [ ] change in weather patterns [ ] less rain [ ] No answer [ ] Other ________________ [ ] N/A

(30). Name up to 3 ways in which you believe which you can do to address/mitigate/adopt to the effects of climate change (RED OUT OPTIONS-SELECT UP TO 3).

[ ] Mangrove planting [ ] Establishment of additional marine sanctuaries or increasing existing marine sanctuary size [ ] Use of climate-resilient crops [ ] Adopting other livelihoods [ ] Setting up community savings in time sof calamities [ ] Rehabilitation of degraded coral reefs [ ] No answer [ ] Other ________________

Section 6

Climate Change

Now I am to ask you a few general questions about your household and your community. Please answer them as honestly and accurately as possible. Your answer will remain confidential.

(31). Since the implementation of coastal and fisheries program in the municipality, please describe the state in terms of food availability in your household?

[ ] We always had enough food to feed everyone in the household [ ] We sometimes did not have enough food to feed everyone in the household [ ] We often did not have enough food to feed everyone in the household [ ] Refuse to answer

(32). Please answer the following question on a scale of 1 to 10, where 1 = completely dissatisfied and 10=completely satisfied.

A. All things considered, how satisfied are you with your life these days (REMIND THAT THIS IS A SCALE OF 1 to 10).

[ ] 1 [ ] 2 [ ] 3 [ ] 4 [ ] 5 [ ] 6 [ ] 7 [ ] 8 [ ] 9 [ ] 10 [ ] Refuse to answer

(33) Please answer the following question on a scale of 1 to 10, where 1 = completely dissatisfied and 10=completely satisfied.

A. The fishers in my community have the ability to sustainably manage our fishery so that we can benefit from it long into the future. (REMIND THAT THIS IS A SCALE OF 1 to 10).

[ ] 1 [ ] 2 [ ] 3 [ ] 4 [ ] 5 [ ] 6 [ ] 7 [ ] 8 [ ] 9 [ ] 10 [ ] Refuse to answer

B. Most fishers in my community will follow the rules and regulations set forth for our fisheries (REMIND THAT THIS IS A SCALE OF 1 to 10).

[ ] 1 [ ] 2 [ ] 3 [ ] 4 [ ] 5 [ ] 6 [ ] 7 [ ] 8 [ ] 9 [ ] 10 [ ] Refuse to answer

(34). Please rate you agreement with the following statements on a scale from STRONGLY AGREE, AGREE, to DISAGREE and STRONGLY DISAGREE.

A. Generally speaking, most fishers in my community can be trusted.

[ ] Strongly Agree [ ] Agree [ ] Disagree [ ] Strongly Disagree [ ] Refused to Answer

B. It is not difficult for myself and members of my family to maintain fishing practices that is needed to provide for the home

[ ] Strongly Agree [ ] Agree [ ] Disagree [ ] Strongly Disagree [ ] Refused to Answer

C. My family is able to benefit from our fishery as much as any other members of the community.

[ ] Strongly Agree [ ] Agree [ ] Disagree [ ] Strongly Disagree [ ] Refused to Answer

Thank you for all your help in responding to this survey. Your time and effort is greatly appreciated.
