# Supplementary material for: Catalyzing sustainable fisheries management through behavior change interventions
Source: Conserv Biol. 2020 Apr 15;34(5):1176–89. doi: 10.1111/cobi.13475 (PMC7540413; doi:10.1111/cobi.13475)
Supplement: Supplementary file 20 — Supplementary Material [file COBI-34-1176-s020.docx]

**Model KAP Survey Brazil 1**

***Section 1: Enumerator and Survey General Information***

1. Enumerator name
2. Date
3. Interview number
4. Community name

***Section 2: Interviewee General Information***

1. Name
2. Sex
3. Age range
4. Marital status
5. Education level
6. Religion
7. Consider if it would be necessary to include questions about fishing activities depending on each site, for example:
   1. In Prainha: type of species fished
   2. In Delta: types of fishing gear and fishing areas used
   3. In sites where target species are fish: how many food sources (types of fish, plants, seabed material, etc.) have you observed the target species eat?

***Section 3: KAP + IC Questions***

| ToC Category | Cohort Generic | Pirajubae | Canavieiras | Baia do Iguape | Delta do Parnaiba | Prainha do Canto Verde | Cururupu |
| --- | --- | --- | --- | --- | --- | --- | --- |
| **K1** | What RESEX (insert RESEX name) activities can you be involved?  None (if this is the answer go to A questions)  Management body meetings  Fishers associations meetings  Community vigilance  Filling out logbooks – diários de bordo  Providing landing site information - dados de desmbarque de pesca | Respecting bank rotation  Respecting weekly harvesting schedule | Not fishing in the reserves | Implementing family scale farming  Adopting a rotating schedule to harvest in the mangrove | Not fishing in the reserves  Changing fishing gears | Changing fishing gears | Changing fishing gears |
| **K2** | What are the direct effect/impact/ consequence of these activities on your life?    None  They are a waste of my time  They can increase target species stock  Me and my family can/will be able to fish more  They can decrease overfishing  They can decrease my fishing effort  They will preserve the environment | Can change depending on qualitative research | Can change depending on qualitative research | Can change depending on qualitative research | Can change depending on qualitative research | Can change depending on qualitative research | Can change depending on qualitative research |
| **A1** | Do you believe that your participation in the RESEX (insert RESEX name) activities can have an effect/impact/ consequences on your life?  No (if this is the answer go to IC questions)  Yes, they can increase target species stock  Yes, they can decrease overfishing  Yes, they secure my fish catch  Yes, they secure fishing for my children  Yes, they can decrease my fishing effort  Yes, they will preserve the environment | Can change depending on qualitative research | Can change depending on qualitative research | Can change depending on qualitative research | Can change depending on qualitative research | Can change depending on qualitative research | Can change depending on qualitative research |
| **IC1** | In the past six months have you talked to other fishers about participating in the RESEX activities (insert RESEX name)?  No (if this is the answer go to BR or BC questions)  If yes, go to IC2 and IC3 questions |  |  |  |  |  |  |
| **IC2** | Which activities have you talked about?  Management body meetings  Fishers associations meetings  Community vigilance  Filling out logbooks  Providing landing site information  Respect new fishing rules` | Respecting bank rotation  Respecting weekly harvesting schedule | Not fishing in the reserves | Implementing family scale farming  Adopting a rotating schedule to harvest in the mangrove | Not fishing in the reserves  Changing fishing gears | Changing fishing gears | Changing fishing gears  Respect close season |
| **IC3** | Why have you talked about it with them?  Because they are good/important (if this is the answer, ask why they think that and mark the answers in this same question)  Because they are bad/useless  Because they can help improve the target species stock  Because they can help decrease overfishing  Because it’s our obligation  Because they can help secure my fish catch  Because they can help secure my children’s future  Because they can help preserve the environment  Because they can help decrease my fishing effort | Can change depending on qualitative research | Can change depending on qualitative research | Can change depending on qualitative research | Can change depending on qualitative research | Can change depending on qualitative research | Can change depending on qualitative research |
| **BR** | Depends on each site |  |  |  |  |  | Analisar com Mocinha qual a RB necessária para a ameaça escolhida – assitencia técnica? Construção no WS |
| **BC1** | In the past six months have you participated in RESEX activities (insert specific RESEX name)?  If no, go to next section  If yes, go to BC2 and BC3 |  |  |  |  |  |  |
| **BC2** | What activities did you participate in?  Management body meetings  Fishers associations meetings  Community vigilance  Filling out logbooks  Providing landing site information | Respecting bank rotation  Respecting weekly harvesting schedule | Not fishing in the reserves | Implementing family scale farming  Adopting a rotating schedule to harvest in the mangrove | Not fishing in the reserves  Changing fishing gears | Changing fishing gears | Changing fishing gears |
| **BC3** | Approximately how many times per month?  Daily  1 – 5 times  6 – 10 times  11 – 15 times |  |  |  |  |  |  |

***Section 4: Exposure to Campaign Questions***

In the past 6 months have you heard about, participated in activities or seen materials about a Pride Campaign?

- - If no, go to social impact questions.
  - If yes, which ones?
    - Campaign song or radio spots
    - Printed material: posters, flyers, etc.
    - Give-aways: t-shirts, hats, bags, etc.
    - Murals
    - Community mobilization events: festivals, school activities, etc.
    - Heard about it from family
    - Heard about it from friends
    - Heard about it from the management body
    - Heard about it from ICMBio
    - Heard about it from local leaders: mayor, fishermen’s association president

***Section 5: Sustainable Livelihood Questions***

***Fishery Governance Participation***

1. Are you or someone in your household an active member of the management body?

[1] Yes [2] No [3] Not sure

***Household Assets***

*2. [Interviewer: without asking, record the main materials of the walls and roof of the house.]*

Walls *[Select only one]* Roof *[Select only one]*

[1] Bamboo [1] Thatch/leaves

[2] Wood [2] Tile

[3] Corrugated iron [3] Corrugated iron

[4] Brick/cement [4] Concrete

[5] Other ____________ [5] Other _____________

*3. [For the following question, read each item one at a time and ask the respondent to tell you whether they have at least one in their household. Record 0 for No and 1 for Yes.]*

For each of the following items, can you tell me whether your household has at least one?

| Item | Present in household?  [0=No, 1=Yes] |
| --- | --- |
| Running water (inside house) |  |
| Electricity |  |
| Refrigerator |  |
| Electric stove/oven |  |
| Radio |  |
| TV set |  |
| Satellite dish |  |
| Watch or clock |  |
| Bicycle |  |
| Canoe or boat without motor |  |
| Motorized boat |  |
| Motorcycle or scooter |  |
| Car or truck |  |

***Household Food Security***

4. Now I would like to ask a couple of questions about the food eaten in your household. Which of these statements best describes the food eaten in your household in the last 12 months?

[1] We always had enough food to feed everyone in the household.

[2] We sometimes did not have enough food to feed everyone in the household.

[3] We often did not have enough food to feed everyone in the household.

[4] We never had enough food to feed everyone in the household.

[*If ‘always had enough,’ skip to question 10. If ‘sometimes did not have, often did not have or never had enough,’ proceed to question 8.*]

5. Here are some reasons why people do not always have enough to feed everyone in their household. For each one, please tell me if that is a reason why *your household* does not always have enough to eat. [*Respondents can select as many as apply.*]

[1] We did not always have enough money.

[2] There were too many people to feed.

[3] It was sometimes too hard to get to the market/store.

[4] There was not always enough food available at the market/store.

[5] There was not enough food during certain seasons/times of year.

[6] Other

1. If you responded ‘Other’ to question 4, please specify the reason why your household did not always have enough to eat.

_________________________________________________________________

***Employment Stability***

1. Which forms of employment or productive activities related to [the fishery] are you or someone in your household involved in on either a full-time or part-time basis? *[Select as many as apply] [These response choices must be customized according to local relevance, based on preliminary qualitative research at site]*

[1] Fishing [5] A

[2] X [6] B

[3] Y [7] C

[4] Z [8] Other

If you responded ‘Other,’ please specify:

__________________________________________________________

1. On average, how many days per month are you (or the other person in your household) spending at this form of employment or productive activity related to [the fishery]?

[1] Less than 5 [4] 16 to 20

[2] 6 to 10 [5] 21 to 25

[3] 11 to 15 [6] More than 25

1. What are your household’s other sources of income that are not directly related to [the fishery] as described above? *[These response choices must be customized according to local relevance, based on preliminary qualitative research at site]*

[1] X [4] A

[2] Y [5] B

[3] Z [6] Other

If you responded ‘Other,’ please specify:

_______________________________________________________________

10. Compared to 5 years ago, the quality and stability of employment for my household has:

[1] Worsened substantially [4] Improved somewhat

[2] Worsened somewhat [5] Improved substantially

[3] Stayed the same [6] Don’t know

1. Based on all forms of employment above which you indicated are important to your household, please rate your agreement with the following statement on a scale from strongly disagree to strongly agree.

It is easy for myself and members of my household to find as much employment as is needed to provide for the household.

[1] Strongly disagree

[2] Disagree

[3] Neither agree nor disagree

[4] Agree

[5] Strongly agree

***Subjective Well-being***

1. Please answer the following question on a scale of 1 to 10, where 1=completely dissatisfied, and 10=completely satisfied.

All things considered, how satisfied are you with your life these days?

[1] [2] [3] [4] [5] [6] [7] [8] [9] [10]

***Collective Efficacy***

1. Please rate your agreement with the following statement on a scale of 1 to 10, where 1=to no extent, 5=to some extent, and 10=to a great extent.

[My community] has the ability to sustainably manage [our fishery] so that we can benefit from it long into the future.

[1] [2] [3] [4] [5] [6] [7] [8] [9] [10]

***Political Trust***

1. Please rate your agreement with the following statement on a scale from strongly disagree to strongly agree.

Generally speaking, the [local government] can be trusted to make decisions in the best interest of [our community].

[1] Strongly disagree

[2] Disagree

[3] Neither agree nor disagree

[4] Agree

[5] Strongly agree

15. Please rate your agreement with the following statement on a scale of 1 to 10, where 1=to no extent, 5=to some extent, and 10=to a great extent.

I am confident that the [TURF-Reserve Management Body] will make the right decisions about how to manage [our fishery].

[1] [2] [3] [4] [5] [6] [7] [8] [9] [10]

***Social Trust***

1. Please rate your agreement with the following statement on a scale of 1 to 10, where 1=to no extent, 5=to some extent, and 10=to a great extent.

Most people in [my community] will follow the rules and regulations set forth for [our community’s fishery].

[1] [2] [3] [4] [5] [6] [7] [8] [9] [10]

17. Please rate your agreement with the following statement on a scale of 1 to 10, where 1=to no extent, 5=to some extent, and 10=to a great extent.

Most people in [my community] will actively enforce the rules and regulations of [our fishery], including reporting violations when witnessed.

[1] [2] [3] [4] [5] [6] [7] [8] [9] [10]

1. Please rate your agreement with the following statement on a scale from strongly disagree to strongly agree.

Generally speaking, most people in [my community] can be trusted.

[1] Strongly disagree

[2] Disagree

[3] Neither agree nor disagree

[4] Agree

[5] Strongly agree

***Social Equity***

1. Please rate your agreement with the following statement on a scale from strongly disagree to strongly agree.

My household is able to benefit from [our community’s fishery] as much as any other members of the community.

[1] Strongly disagree

[2] Disagree

[3] Neither agree nor disagree

[4] Agree

[5] Strongly agree

1. Please rate your agreement with the following statement on a scale from strongly disagree to strongly agree. *[This question will only be relevant on surveys conducted after implementation of TURF and allocation of access rights.]*

Access rights to [the TURF] have been distributed fairly to fishers.

[1] Strongly disagree

[2] Disagree

[3] Neither agree nor disagree

[4] Agree

[5] Strongly agree
